# MCP-1/CCR2 axis inhibition sensitizes the brain microenvironment against melanoma brain metastasis progression

**DOI:** 10.1172/jci.insight.154804

**Published:** 2022-09-08

**Authors:** Sabina Pozzi, Anna Scomparin, Dikla Ben-Shushan, Eilam Yeini, Paula Ofek, Alessio D. Nahmad, Shelly Soffer, Ariel Ionescu, Antonella Ruggiero, Adi Barzel, Henry Brem, Thomas M. Hyde, Iris Barshack, Sanju Sinha, Eytan Ruppin, Tomer Weiss, Asaf Madi, Eran Perlson, Inna Slutsky, Helena F. Florindo, Ronit Satchi-Fainaro

**Affiliations:** 1 Department of Physiology and Pharmacology, Sackler Faculty of Medicine, Tel Aviv University, Tel Aviv, Israel.; 2 Department of Drug Science and Technology, University of Turin, Turin, Italy.; 3 The School of Neurobiology, Biochemistry and Biophysics, The George S. Wise Faculty of Life Sciences, and; 4 Dotan Center for Advanced Therapies, Tel Aviv Sourasky Medical Center (TASMC), Tel Aviv University, Tel Aviv, Israel.; 5 Department of Neurosurgery, Johns Hopkins University School of Medicine, Baltimore, Maryland, USA.; 6 Lieber Institute for Brain Development, Johns Hopkins Medical Campus, Baltimore, Maryland, USA.; 7 Department of Pathology, Sheba Medical Center, Tel Hashomer, Israel.; 8 Department of Pathology, Sackler Faculty of Medicine, Tel Aviv University, Tel Aviv, Israel.; 9 Cancer Data Science Laboratory, Center for Cancer Research, National Cancer Institute, NIH, Bethesda, Maryland, USA.; 10 Department of Pathology, Sackler Faculty of Medicine, and; 11 Sagol School of Neurosciences, Tel Aviv University, Tel Aviv, Israel.; 12 Research Institute for Medicines (iMed.ULisboa), Faculty of Pharmacy, University of Lisboa, Lisbon, Portugal.

**Keywords:** Oncology, Therapeutics, Cancer immunotherapy, Skin cancer

## Abstract

Development of resistance to chemo- and immunotherapies often occurs following treatment of melanoma brain metastasis (MBM). The brain microenvironment (BME), particularly astrocytes, cooperate toward MBM progression by upregulating secreted factors, among which we found that monocyte chemoattractant protein-1 (MCP-1) and its receptors, CCR2 and CCR4, were overexpressed in MBM compared with primary lesions. Among other sources of MCP-1 in the brain, we show that melanoma cells altered astrocyte secretome and evoked MCP-1 expression and secretion, which in turn induced CCR2 expression in melanoma cells, enhancing in vitro tumorigenic properties, such as proliferation, migration, and invasion of melanoma cells. In vivo pharmacological blockade of MCP-1 or molecular knockout of CCR2/CCR4 increased the infiltration of cytotoxic CD8^+^ T cells and attenuated the immunosuppressive phenotype of the BME as shown by decreased infiltration of Tregs and tumor-associated macrophages/microglia in several models of intracranially injected MBM. These in vivo strategies led to decreased MBM outgrowth and prolonged the overall survival of the mice. Our findings highlight the therapeutic potential of inhibiting interactions between BME and melanoma cells for the treatment of this disease.

## Introduction

Cutaneous melanoma is the deadliest of all skin cancers, especially due to its tendency to invade and develop metastases at distant sites. It is the third primary malignancy after lung and breast cancers that preferentially colonizes the brain, with an incidence of brain metastasis development of 40% to 50% in patients with melanoma stage IV (although the incidence postmortem is 70%–90%) (1, 2). Without any therapeutic intervention, metastatic lesions lead to a median survival of less than 7 months. The current standard treatments, including radiation, chemotherapy, and targeted therapies, raised the overall survival of almost 85% of patients diagnosed with melanoma brain metastasis (MBM) from 12 to 14 months (3). Recent advances in immunotherapy raised the overall survival to 2 years; however, heterogeneity in immune responses and resistance to treatments are frequently observed due to distinct adaptive and innate immune cells’ infiltration into the tumor (3–6).

The brain microenvironment (BME) represents the first line of reaction in favor or against the tumor due to its ability to generate an immune-stimulatory or immunosuppressive niche. This will ultimately determine the establishment and growth of MBM (5, 7). Among the BME-resident cells, astrocytes are responsible for the maintenance of brain homeostasis (8). Subsequent to melanoma brain colonization, alterations in astrocyte morphology and protein expression are generally observed and considered hallmarks of neuroinflammation (9, 10). Indeed, the activation of astrocytes induces the upregulation of glial fibrillary acid protein (GFAP) and secretion of growth factors, fatty acids, and brain damage-related factors. Together, these changes allow the astrocytes to sustain and foster the growth and development of primary and secondary brain lesions (11, 12).

Here, we investigated the role of a proinflammatory chemokine, monocyte chemoattractant protein-1 (MCP-1, also known as CCL2) in MBM progression. MCP-1 is physiologically expressed by a variety of brain cells, such as neurons, microglia, endothelial cells, and astrocytes (13). However, an increase in astrocyte-secreted MCP-1 was reported in several inflammatory processes that occur in the CNS, following brain damage or diseases, including cancer and brain metastasis (14–16). In previous cancer-related studies, the loss of phosphatase and tensin homolog (PTEN) in melanoma cells induced by astrocytes resulted in MCP-1 oversecretion by melanoma cells, and in the recruitment of Iba1 and macrophages that reciprocally improved the outgrowth of brain metastatic tumor cells via enhanced proliferation and reduced apoptosis (17). Moreover, the oversecretion of MCP-1 from astrocytes enhanced angiogenesis and contributed to the loss of BBB integrity, by disassembling tight junction proteins and altering microvessel permeability (18–20). MCP-1 overexpression by astrocytes was shown to play a major role in mediating CCR2-expressing breast and lung cancer cell extravasation into the brain in 3D and in vivo models (16). Therefore, an aberrant expression of MCP-1 may sustain tissue inflammation and exacerbate the progression of the disease (21). Here, we demonstrated that astrocyte-secreted MCP-1 mediated the interaction of these brain cells with melanoma, and reshaped the local immune microenvironment, which is pivotal for the progression of MBM.

## Results

### Activated astrocytes sustain melanoma cell growth, invasion, and migration.

Our first aim was to elucidate the mechanism by which activated astrocytes contribute to MBM progression. Although several studies pointed out potential factors involved in promoting the interaction between astrocytes and melanoma cells, the benefit of their inhibition in terms of clinical relevance is yet to be fully elucidated (10, 22). Previous publications have shown the contribution of astrocyte-secreted cytokines to melanoma cell proliferation and migration in the Mel-ret murine melanoma model; WM983BA, 375P, DM-4 (from lymph node metastasis specimens), YDFR.CB3, and TXM-13 human melanoma; and the WM4265-2 human melanoma brain metastatic variant (10, 23–25). Here, we strengthened this observation by using alternative murine (D4M.3A, B2905, B16F10) and human (131/4-5B1, A375, WM115) melanoma models and clinical samples that present different origins, mutations (BRAF, PTEN, etc.), and responsiveness to immunotherapy. We showed positive staining for GFAP-activated astrocytes surrounding melanoma metastases in a cohort of 40 patients diagnosed with MBM bearing WT BRAF (patient-derived 1–5, PD1–PD5, Supplemental Figure 1A; supplemental material available online with this article; https://doi.org/10.1172/jci.insight.154804DS1). This activation was also confirmed by histological analysis of our MBM mouse models of human and murine melanoma bearing different BRAF mutations (intracardially inoculated mCherry-labeled WM115 [BRAF V600D], D4M.3A [BRAF V600E], or 131/4-5B1 [BRAF V600D] melanoma cells) (Supplemental Figure 1A). GFAP-activated astrocytes were also observed in healthy human and murine brain samples but to a much lower extent under physiological conditions (Supplemental Figure 1A) (26). Following intracardiac cell inoculation of melanoma cells (B2905, D4M.3A, WM115 and 131/4-5B1), different models showed variability in MBM formation and location, as well as in the incidence of metastasis to other organs (Tables 1 and 2 and Supplemental Figures 2–4).

Thus, to study melanoma-astrocyte interactions in a more reproducible model of brain metastasis only, intracranial cell inoculation following the excision of the primary melanoma lesion was included in the study. The intracranial inoculation per se induces local neuroinflammation (27), mostly affecting resident cells (e.g., astrocytes and microglia), and could interfere with their activation and interaction with the cancer cells in the initial steps of brain colonization (28). To subtract the secondary effects associated with the intracranial injection, we also stained for GFAP activation in brain cryosections following sham injury or PBS administration. Then, we compared the morphology and GFAP expression of astrocytes of these control samples to those of astrocytes in B16-F10 and D4M.3A MBM samples (Supplemental Figure 1B). Only basal activation was detected in the samples with the sham injury after 3 days. Intermediate GFAP activation and presence of isomorphic astrocytes were found at the injection site of the PBS group (Supplemental Figure 1B). Indeed, the short-term (day 3) expression indicated physiological brain functionality as it decreased by day 10 (Supplemental Figure 1C) (29). Nonetheless, a high level of reactive astrocytes was observed only in tumor-bearing brain tissue from mice, 10 days following melanoma cell inoculation (Supplemental Figure 1, B and C). These findings support the feasibility of using an intracranially injected model of MBM for investigations involving the late contribution of GFAP-expressing astrocytes in MBM progression.

**Figure 1:**
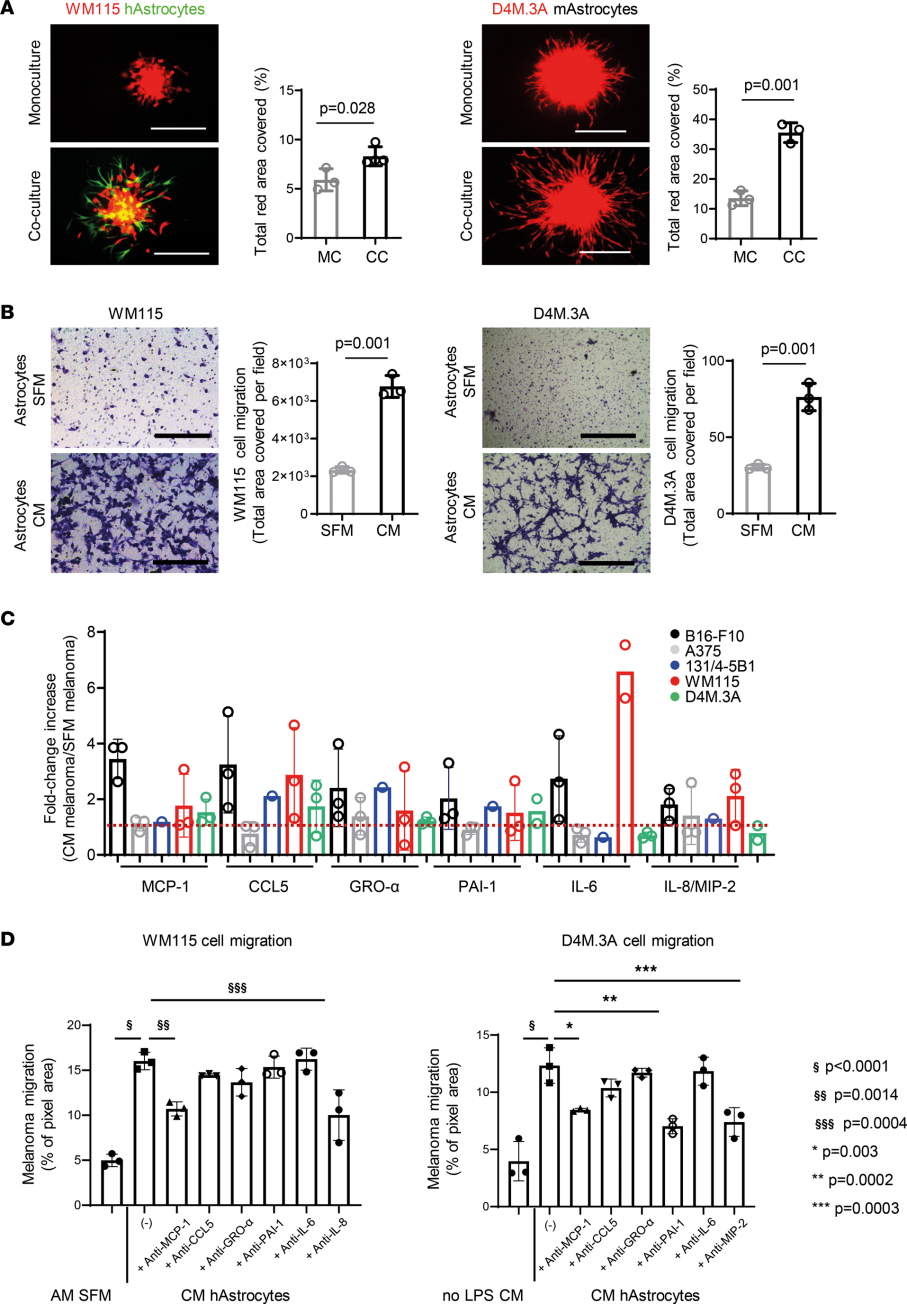
MCP-1 is a major astrocyte-secreted factor that influences melanoma migration. (**A**) Coculture with astrocytes and melanoma 3D multicellular spheroid (WM115 and D4M.3A mCherry-labeled melanoma cells). Mouse astrocytes unlabeled. Human astrocyte GFP-labeled in green. Dots represent melanoma cell invasion as fluorescence signal of mean ± SD of *n* = 3 spheroids/well of *n* = 3 wells of 3 independent experiments (representative image of 1 independent experiment). Nonparametric Student’s 2-sided *t* test. Scale bar — 400 μm. (**B**) Astrocyte conditioned media (CM — black) enhance melanoma Transwell migration (WM115 and D4M.3A cells). Pixel density (total area covered per field) of the migrated cancer cells. Representative cell-migrating fields are shown (*n* = 3). Scale bar — 400 μm. Mean ± SD of *n* = 3 fields/well of 3 wells of 3 independent experiments. Nonparametric Student’s *t* test. (**C**) Levels of 6 proinflammatory astrocyte-secreted cytokines in CM of melanoma cells (B16-F10 — black, A375 — gray, 131/4-5B1 — blue, WM115 — red, and D4M.3A — green) or alternatively in SFM of melanoma cells for 24 hours. Fold change ± SD of 3 independent biological repeats. (**D**) Melanoma cells (D4M.3A and WM115) were allowed to migrate in the presence of SFM or astrocyte CM (AS CM) in the presence or absence of neutralizing antibodies. AS CM without LPS and AS CM were used as negative controls (NCs) for cell migration in D4M.3A and WM115, respectively. *n* = 3, mean ± SD of replicates of 3 independent biological repeats. One-way ANOVA. ^§^*P* < 0.0001, ^§§^*P* = 0.0014, ^§§§^*P* = 0.0004, *P = 0.003, **P = 0.0002, ***P = 0.0003.

We further investigated the direct effect of GFAP-activated astrocytes in promoting melanoma cell proliferation and invasion in coculture. To study these effects, astrocytes were cocultured with mCherry-labeled human or murine melanoma cells (WM115, A375, D4M.3A, B16-F10, B2905 cells). Our results show that astrocytes facilitated melanoma cell proliferation by 2- to 4-fold compared with melanoma cells grown in monoculture (Supplemental Figure 5A). Melanoma cell invasion capacity was evaluated in 3D multicellular spheroids composed of melanoma cells grown in monoculture or coculture with astrocytes. We found the sprouting capability of mCherry-labeled melanoma cells was significantly enhanced (25% in WM115, 75% in D4M.3A, and 55% in B2905 cells) following coculture with astrocytes (Figure 1A and Supplemental Figure 6B). In some cases (A375 and B16-F10 cells), the addition of astrocytes to the monocultures of melanoma cells was essential for the formation of 3D multicellular spheroids (Supplemental Figure 6B).

As we speculate that the first step of melanoma brain colonization and spreading within the brain parenchyma is mediated by activated astrocytes and followed by cancer cell-astrocyte interactions, we evaluated the effect of stress-induced activated astrocytes (30) and collected the conditioned media (CM) containing growth factors and cytokines secreted by astrocytes. We observed that the melanoma cells migrated faster toward the astrocyte CM compared with serum-free medium (SFM) in a Transwell migration assay (3-, 1.2-, 4-, 1.6-, 1.8-, and 2-fold increase in WM115, A375, D4M.3A, B16-F10, B2905, and 131/4-5B1 cells, respectively, Figure 1B and Supplemental Figure 6A). These results suggest that cytokines or growth factors secreted by astrocytes can interact with and promote melanoma cell motility prior to their direct interactions.

### MCP-1 has a key role in melanoma chemoattraction toward astrocyte CM.

**Figure 2:**
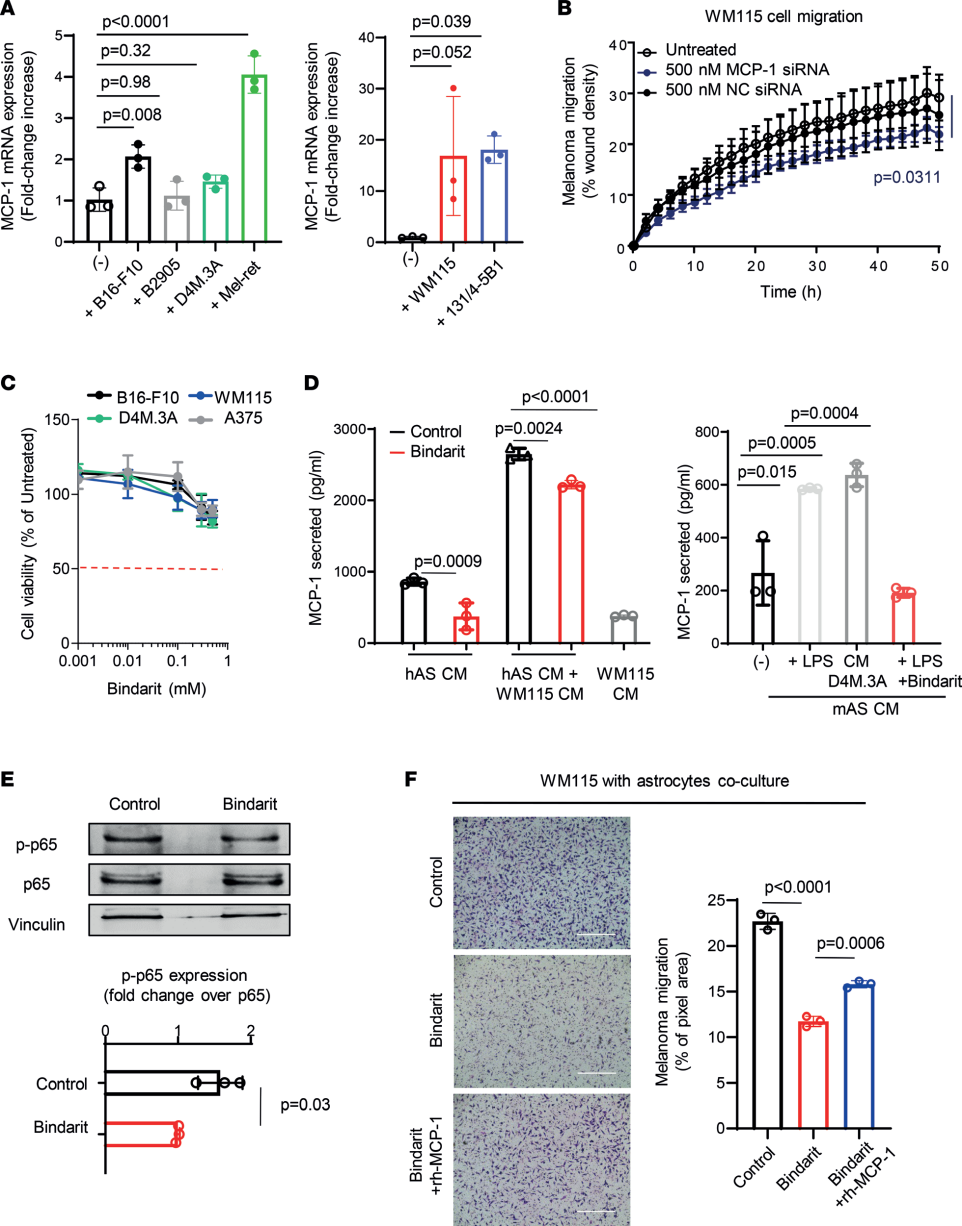
Inhibition of astrocyte-secreted MCP-1 decreases melanoma migration. (**A**) *MCP-1* mRNA in murine astrocytes cocultured with melanoma cells (B16-F10 — black, B2905 — gray, D4M.3A — dark green, Mel-ret — green) or human astrocytes cocultured in SFM with melanoma cells (WM115 — red, 131/4-5B1 — blue). Mean ± SD of 3 independent biological repeats. Ordinary 1-way ANOVA. (**B**) Wound healing migration assay of WM115 cells grown in AS CM (untreated — black empty dot), siRNA:PEI targeting MCP-1 (blue), or NC (black full dot). Mean ± SD of *n* = 2 fields per well biological replicates (*n* = 3). Two-way ANOVA. (**C**) B16-F10 — in black, D4M.3A — in green, WM115 — in blue, A375 — in gray exposed to bindarit for 72 hours. Tumor cell viability assessed by metabolic activity MTT assay. Mean ± SD of triplicates of 3 independent biological repeats. Nonparametric Student’s *t* test. (**D**) Secretion of MCP-1 in the CM of untreated, bindarit-treated astrocytes, melanoma CM-treated astrocytes, or melanoma CM-treated astrocytes treated with bindarit. Basal secretion of MCP-1 in WM115 melanoma cells (gray). Human astrocyte CM (hAS CM) (black); bindarit-treated astrocyte CM (red). Unactivated astrocytes (black), LPS-activated (light gray), melanoma CM-treated astrocytes (gray), bindarit-treated astrocytes (red). Mean ± SD of triplicates of 3 independent biological repeats. One-way ANOVA. (**E**) Western blot of phosphorylated p65 (p-p65) in bindarit-treated astrocytes. Density bands of 1 representative experiment. p-p65 signal is presented as fold change expression relative to p65 expression. Density bands of p-p65 and p65 were normalized by vinculin (loading control). Mean ± SD of triplicates of 3 independent biological repeats. (**F**) Transwell migration of melanoma WM115 cells toward untreated AS (black), bindarit-treated AS (red), or bindarit-treated AS supplemented with rh-MCP-1 (blue). Representative fields of WM115 migrated cells (*n* = 3). Scale bar — 400 μm. Mean ± SD of biological replicates (*n* = 3). One-way ANOVA.

To identify changes in the astrocyte secretome upon paracrine interactions with melanoma cells, we performed a cytokine array using melanoma-activated astrocyte CM. The secretion levels of 6 out of 100 cytokines involved in tissue damage response and cell chemotaxis (i.e., MCP-1/CCL2, RANTES/CCL5, GRO-α/CXCL1, SERPIN E1/plasminogen activator inhibitor-1 [PAI-1], IL-6, and IL-8/macrophage inflammatory protein 2 [MIP-2]), were upregulated (2, 2.1, 1.7, 1.1, 1.4, and 1.6 average fold increase, respectively) in astrocytes grown in CM of several melanoma cell lines (B16-F10, A375, 131/4-5B1, WM115, and D4M.3A) (Figure 1C, representative array membranes shown in Supplemental Figure 7A). Following the inhibition of the activity of the selected cytokines by adding neutralizing antibodies to the astrocyte CM, both murine D4M.3A and human WM115 melanoma cell Transwell migration were reduced compared with cells grown in control astrocyte CM (Figure 1D). The strongest and most statistically significant migration inhibition was achieved with anti–MCP-1 (33% in D4M.3A and 40% in WM115 cells) and anti–IL-8/MIP2 (45% in D4M.3A and 35% in WM115 cells) antibodies. Indeed, MCP-1 was found to be a potent factor for melanoma migration in all the cell lines tested, including human primary and metastatic melanoma (WM115 and 151/4-5B1) and murine primary melanoma cell lines (D4M.3A and B16-F10) — 40% and 50% migration inhibition (Figure 1D and Supplemental Figure 7B). Recombinant human MCP-1 (rh-MCP-1) enhanced melanoma cell migration in a wound healing assay (5% increase in WM115, 27% increase in 131/4-5B1), whereas MCP-1 neutralizing antibody reduced wound closure (7% reduction in WM115, 66% reduction in 131/5B1) compared with cells that migrated in astrocyte SFM or astrocytes CM (Supplemental Figure 7C). Nevertheless, coculturing astrocytes with B16-F10, B2905, D4M.3A, and Mel-ret murine melanoma cells or WM115 and 131/4-5B1 human melanoma cells led to 2.1-, 1.1-, 1.5- and 4-fold increase or 15-fold and 15-fold increase, respectively, in their *MCP-1* mRNA expression compared with astrocytes grown as monoculture (Figure 2A).

To strictly address the importance of astrocyte-secreted MCP-1 in attracting melanoma cells and promoting their growth, we compared the ability of melanoma cells to migrate in coculture toward astrocytes, microglia, or endothelial cells (known to be the alternative sources of MCP-1 in the brain), following neutralization of MCP-1 secretion. We observed that the neutralization of MCP-1 reduced melanoma cell migration toward the relevant brain-resident cells with a statistically significant difference toward microglia (*P* = 0.024) and astrocytes (*P* = 0.052) but not toward endothelial cells (Supplemental Figure 8A). MCP-1 neutralization did not affect the proliferation of melanoma cells when cocultured with microglia, but it did inhibit their proliferation when cocultured with astrocytes or endothelial cells (Supplemental Figure 8B). We can conclude that the neutralization of MCP-1 in CM of astrocyte-melanoma cell coculture inhibited melanoma cell migration and proliferation, and it did not affect the viability of the brain-resident cells (Supplemental Figure 9A). Astrocytes, microglia, and endothelial cells express and secrete MCP-1, as demonstrated by its expression and secretion in their intracellular compartments and in their CM when stimulated in SFM or when cocultured with WM115 melanoma cells (Supplemental Figure 9, B and C). However, when in coculture with melanoma cells, astrocytes expressed and secreted MCP-1 to a higher extent compared with microglia and endothelial cells (Supplemental Figure 9, B and C).

To inhibit *MCP-1* mRNA, we exploited siRNA to knock down its expression in human astrocytes. Astrocytes treated with siRNA:polyethylenimine (PEI) polyplex expressed less than 20% of the total *MCP-1* mRNA compared with untreated or NC siRNA-treated cells (Supplemental Figure 10A). The siRNA:PEI polyplexes did not cause any off-target effects on astrocyte viability (Supplemental Figure 10A). Moreover, ELISA quantification of astrocyte CM following treatment with siRNA:PEI polyplex showed a remarkable reduction in MCP-1–secreted chemokine (80% of the untreated) (Supplemental Figure 10A). The siRNA-treated astrocyte-derived CM added to WM115 or 131/4-5B1 melanoma cells led to 20% to 38% decrease of the migration ability of melanoma cells compared with the same cells grown in untreated astrocyte CM or astrocytes treated with negative control siRNA (NC:PEI) (Figure 2B and Supplemental Figure 10B). The neutralization or silencing of MCP-1 resulted in a decrease of 131/4-5B1 melanoma cell migration (Supplemental Figure 7C and Supplemental Figure 10B). This effect was more pronounced in the 131/4-5B1 brain metastasis cell line than in the WM115 melanoma cell line (Figure 2B and Supplemental Figure 10B).

### The MCP-1 inhibitor bindarit inhibits melanoma cell migration and invasion in astrocyte-melanoma cells coculture.

The establishment of astrocytes-melanoma cells interactions in which MCP-1, overexpressed by astrocytes, enhanced in vitro melanoma tumorigenic properties (proliferation, migration, and invasion) drove us to further evaluate the effect of inhibiting MCP-1 via bindarit. Bindarit did not affect the viability of human or murine melanoma cells, at all concentrations used when grown as monoculture (Figure 2C). As resident brain cells secrete basal levels of MCP-1, we set out to verify that bindarit will not be toxic to these cells. To that end, we showed that treatment with bindarit did not affect the viability of human brain-resident cells (astrocytes, microglia, endothelial cells, and human hippocampal neurons) (Supplemental Figure 10, C and D). Moreover, the ability of these brain-resident cells to migrate was not inhibited by the inhibition of MCP-1 (neutralizing antibody or bindarit at 0.3 mM, Supplemental Figure 10E), meaning that MCP-1 inhibition does not affect the viability or the motility of normal brain-resident cells. Bindarit efficiently inhibited MCP-1 secretion from melanoma- or SFM-activated human astrocytes and in LPS-stimulated murine astrocytes by 0.3- or 3- and 4-fold, respectively, whereas CM of melanoma cells (WM115 and D4M.3A) enhanced the basal secretion of MCP-1 by 3-fold (Figure 2D). MCP-1 secreted into the CM of bindarit-treated astrocytes was 50% of that of untreated astrocytes, whereas few or no effects were observed for the other targets previously found upregulated in the cytokine arrays of activated astrocytes (Supplemental Figure 11A). Indeed, bindarit, a selective regulator of the classical NF-κB inflammatory pathway, acts as an inhibitor of MCP-1 promoter activation by blocking the phosphorylation of IκBα and p65, a downstream effector of the NF-κB signaling pathway, and halting the production of MCP-1 (31). To that end, we evaluated the selective inhibition of MCP-1 via the downregulation of p65 phosphorylation in astrocytes activated by melanoma CM supplemented with bindarit. Phosphorylation of p65 was induced by exposing astrocytes to WM115 CM, while its inhibition was observed in these stimulated astrocytes when exposed to melanoma WM115 CM followed by the treatment with bindarit (Figure 2E; see complete unedited blots in the supplemental material).

The inhibition of MCP-1 production resulted in decreased melanoma cell migration (D4M.3A, B16-F10, A375, 131/4-5B1, and WM115) toward astrocytes, when the latter were exposed to bindarit (Supplemental Figure 11B). Moreover, the addition of rh-MCP-1 rescued the inhibition of melanoma cell migration as opposed to those cells that migrated in the presence of bindarit-treated astrocytes (*P* = 0.006) or toward the CM of bindarit-treated astrocytes (*P* = 0.115) (Figure 2F and Supplemental Figure 11C). The CM of bindarit-treated astrocytes decreased melanoma cell motility, whereas minor effects on melanoma cell migration were observed when melanoma cells were migrating toward astrocyte CM of pretreated astrocytes (Supplemental Figure 11D). When we challenged the migration/invasion of melanoma cells by seeding murine brain endothelial cells (mBECs) on the Transwell upper chamber, the neutralization of MCP-1 in astrocytes pretreated with bindarit inhibited melanoma transendothelial cell migration (nearly 15% in D4M.3A and 50% in B16-F10) (Supplemental Figure 12A). When astrocytes were directly treated while in coculture with melanoma cells, 50% inhibition of D4M.3A and B16-F10 transendothelial cell migration was achieved (Supplemental Figure 12B). Moreover, the invasion ability of bindarit-treated mCherry-labeled melanoma multicellular 3D spheroids in Matrigel (WM115 cells or D4M.3A) was significantly inhibited when cocultured with astrocytes compared with untreated 3D coculture (Supplemental Figure 13, A and B). In the absence of astrocytes, the sprouting ability of melanoma cells was not affected by bindarit, when melanoma multicellular 3D spheroids were grown in astrocyte SFM or in astrocytes CM (Supplemental Figure 13, A and B). Thus, even though the migration and invasion abilities of melanoma cells are enhanced in the presence of astrocytes or of alternative brain-resident cells, this phenotype can be altered by blocking MCP-1 secretion from astrocytes.

### Treatment with bindarit delays tumor progression in mice bearing B16-F10 intracranial MBM.

**Figure 3:**
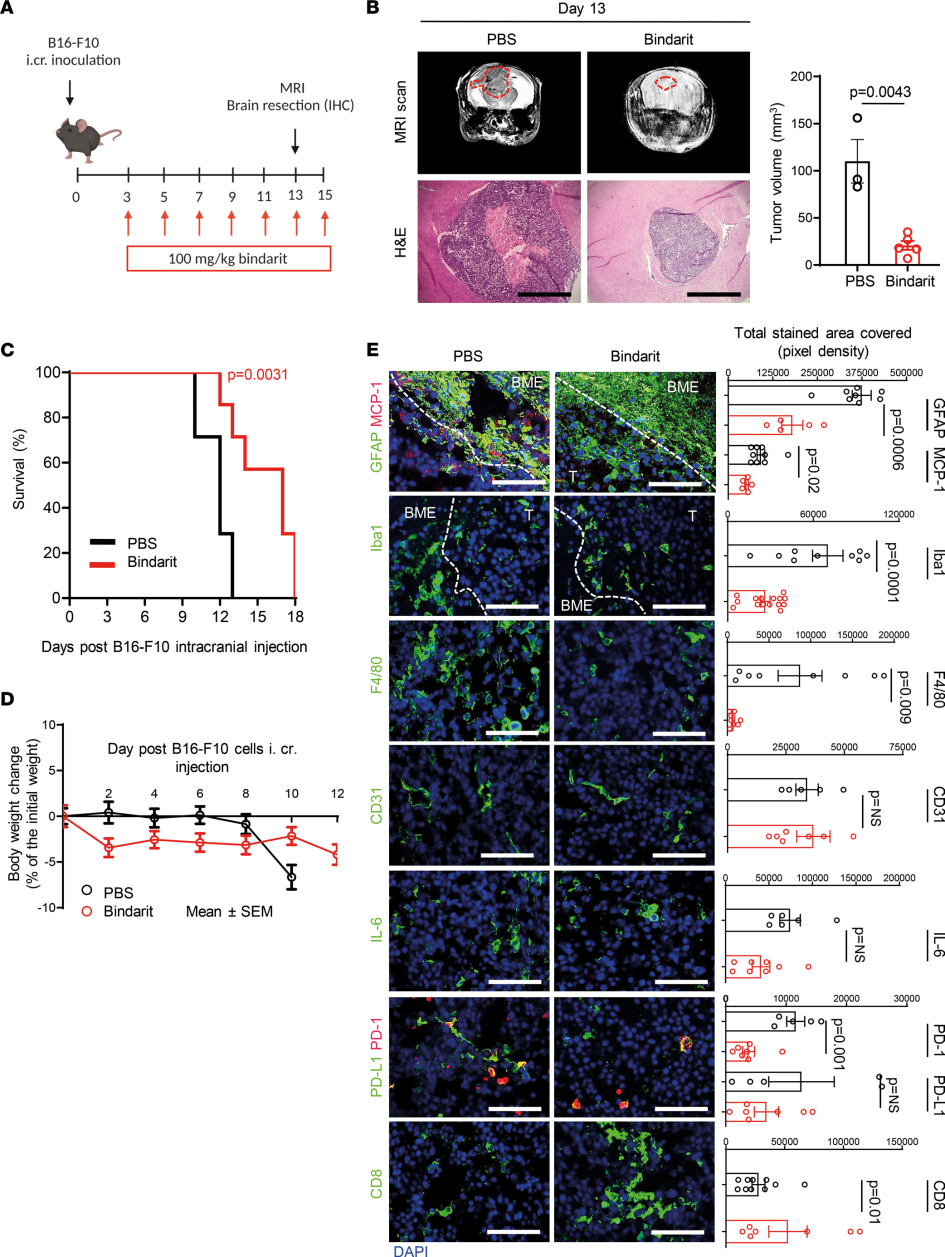
MCP-1 inhibition delays B16-F10 MBM progression. (**A**) B16-F10 melanoma cells were intracranially (i.cr.) inoculated in immunocompetent C57BL/6 mice to generate brain metastasis (*n* = 20). Three days after tumor cell inoculation into the brain, mice were treated with 100 mg/kg i.v. bindarit (*n* = 10) or PBS (*n* = 10) QOD until day 15. Image created with BioRender.com. (**B**) Tumor size in MRI scans at day 13. Representative images of mice bearing brain tumors in PBS- or bindarit-treated groups (*n* = 10); H&E staining for tumor morphology and size. Scale bar — 400 μm. Quantification of tumor size in B16-F10 brain tumor. Mean ± SD (*n* = 3 PBS; *n* = 5 bindarit). Nonparametric Student’s *t* test. (**C**) Kaplan-Meier survival curve (*n* = 7). Two-tailed *P* values from log-rank (Mantel–Cox). (**D**) Mouse body weight change was monitored twice a week upon injection of B16-F10 melanoma cells. Mean ± SEM of *n* = 10 per group. (**E**) Brain cryosections at day 13 (*n* = 3) were stained for MCP-1 (red), GFAP (activated astrocytes — green), Iba1 (activated microglia/macrophages — green), IL-6 (green), F4/80 (macrophages — green), CD31 (blood vasculature — green), PD-1/PD-L1 (exhausted T cells/inhibitory molecule — red/green), and CD8^+^ (cytotoxic T cells — green) markers. Nuclei are shown by DAPI staining (blue). Scale bar — 100 μm. Mean ± SEM of *n* = 7–10 fields per marker in *n* = 3 mice per group. Nonparametric Student’s *t* test. PD-1/PD-L1, programmed cell death 1/programmed cell death ligand 1; T, tumor.

The use of spontaneous (syngeneic) mouse models is limited by highly variable incidence of brain metastasis (50% from direct systemic inoculation of nontropic melanoma cells, less than 20% from orthotopic inoculation following primary tumor resection; ref. 28) and may be affected by the simultaneous development of metastasis to other distant organs (Table 2). Therefore, we used intracranial models for interventional study as a strategy to reduce variability in number, location, and yield of brain metastases among the treated and control groups. To interfere with melanoma-astrocyte and BME interactions via MCP-1 inhibition, in vivo therapeutic studies were carried out by i.v. administration of bindarit (100 mg/kg in PBS) every other day (QOD) in mice bearing the highly aggressive intracranially injected B16-F10 melanoma (Figure 3A). At the dose and under the treatment regimen selected for the in vivo studies, bindarit did not cause RBC lysis (Supplemental Figure 14A). On day 13, MRI analysis showed a remarkable inhibition of tumor growth following treatment with bindarit (Figure 3B). Inhibition of MCP-1 resulted in 5-fold reduction in tumor size compared with the PBS-treated group (Figure 3B), and a discrete, although significant, prolongation of mouse survival was obtained (Figure 3C). In addition, no notable body weight change was observed (Figure 3D). Cryosections of brain samples from day 13, analyzed for tumor morphology (H&E), emphasized the efficacy of the treatment in delaying melanoma progression (Figure 3B). Further histological analysis showed lower levels of MCP-1 secretion from activated astrocytes, moderate reduction in F4/80^+^ and Iba1^+^ microglia/macrophages, and enhanced infiltration of CD8^+^ T cells into tumors resected from mice treated with bindarit (Figure 3E). As MCP-1 is a potent inhibitor of T cell trafficking, we evaluated the expression of immune inhibitory molecules, such as PD-1/PD-L1, in the BME of bindarit-treated MBM. Reduced expression of the coinhibitory PD-1 molecule was observed in the BME, whereas PD-L1 expression was not altered, and no correlation with CD8^+^ T cell infiltration was confirmed (32). Moreover, quantification of microvessel density (CD31 marker) did not point out any significant changes between PBS- and bindarit-treated groups, even though reduction in lumen size of blood vessels was qualitatively seen (Figure 3E). To evaluate if the reduction in lumen size of blood vessels found in bindarit-treated MBM may be less permeable due to the inhibition of MCP-1, we compared vessel permeability within the tumor using a modified Miles assay (33). Mice bearing intracranially inoculated B16-F10 melanoma were treated according to the scheme of treatment presented in Figure 3A. On day 11, besides achieving inhibition of tumor growth (Supplemental Figure 14B), mice treated with bindarit treatment had 50% reduction of Evans blue dye extravasation and extraction from the brain parenchyma (Supplemental Figure 14C). Moreover, early time point (day 7 and 8) treated tumors of matched size showed reduction of dye extravasation into the brain tissue at 45 minutes and 3 hours following bindarit administration (Supplemental Figure 14, D and E).

### MCP-1 inhibition in MBM impairs myeloid-derived suppressor cell infiltration and increases antitumor T cell recruitment.

**Figure 4:**
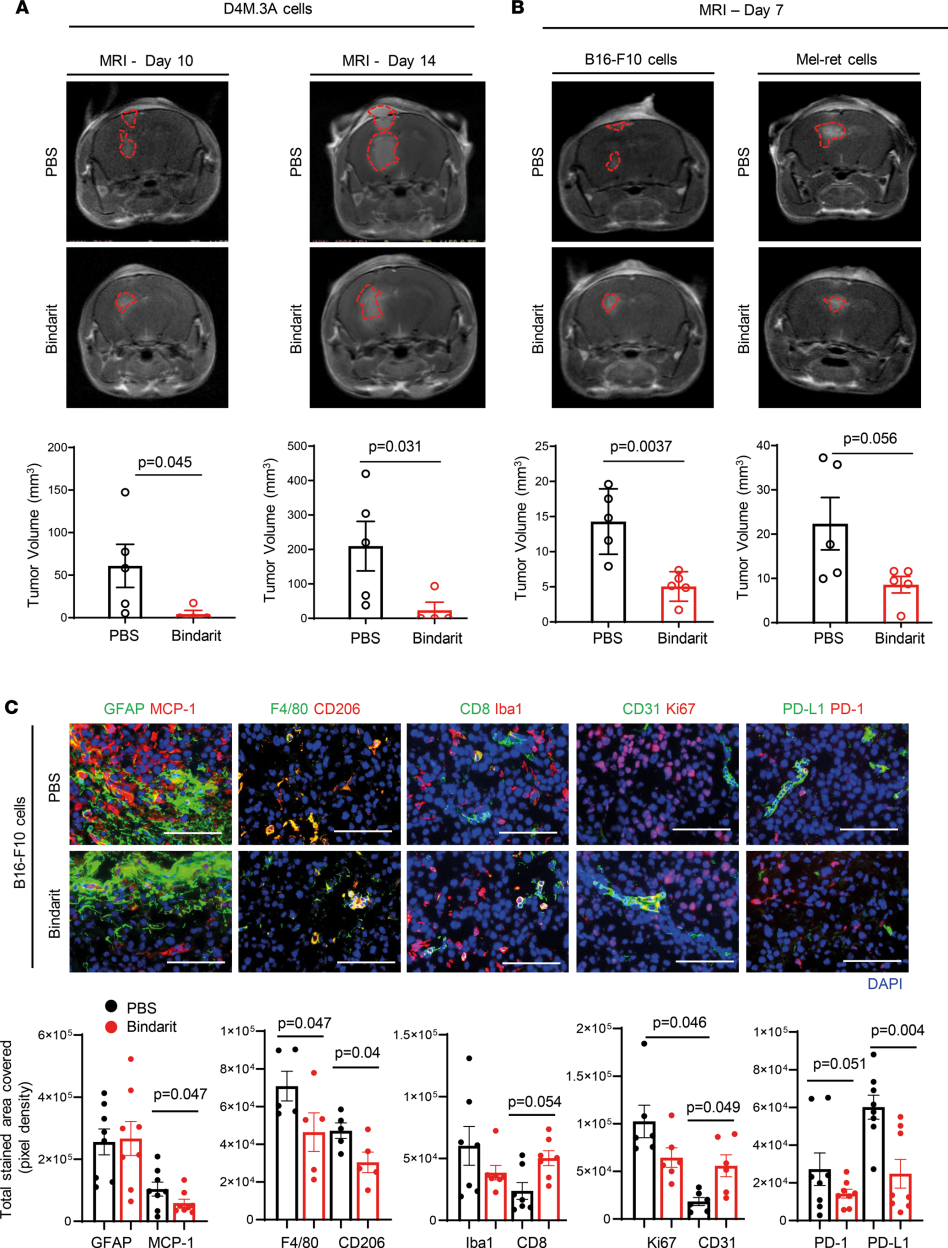
Bindarit treatment improves CD8^+^ T cell infiltration and decreases immune coinhibitory molecules. (**A**) Tumor size and quantification of D4M.3A MBM–bearing mice in MRI scans at day 10 and 14. Representative images of mice bearing brain tumors in PBS- and bindarit-treated group. Mean ± SEM of *n* = 4 in bindarit-treated group, *n* = 5 in PBS-treated group. Nonparametric Student’s *t* test. (**B**) Tumor size and quantification of B16-F10 or Mel-ret MBM–bearing mice in MRI scans at day 8. Representative images of mice bearing brain tumors in PBS- and bindarit-treated group. Mean ± SEM of *n* = 5 in bindarit- and PBS-treated group. (**C**) Histological analysis at day 8 (representative fields of *n* = 5–7 fields per marker in *n* = 3 mice per group) of MCP-1/GFAP (in red and in green, respectively), CD206 staining associated with F4/80^+^ macrophages (in red and in green, respectively), infiltration of CD8^+^ T cells/Iba1^+^ microglia/microphages (red/green), tumor proliferation (Ki67 — in red) and blood vasculature (CD31 — in green), and PD-1/PD-L1 exhausted T cells/inhibitory molecules (red/green) in B16-F10 MBM. Nuclei are shown by DAPI staining (blue). Scale bar — 1,000 μm (H&E) and 100 μm (immunofluorescence). Mean ± SEM. Nonparametric Student’s *t* test.

**Figure 5:**
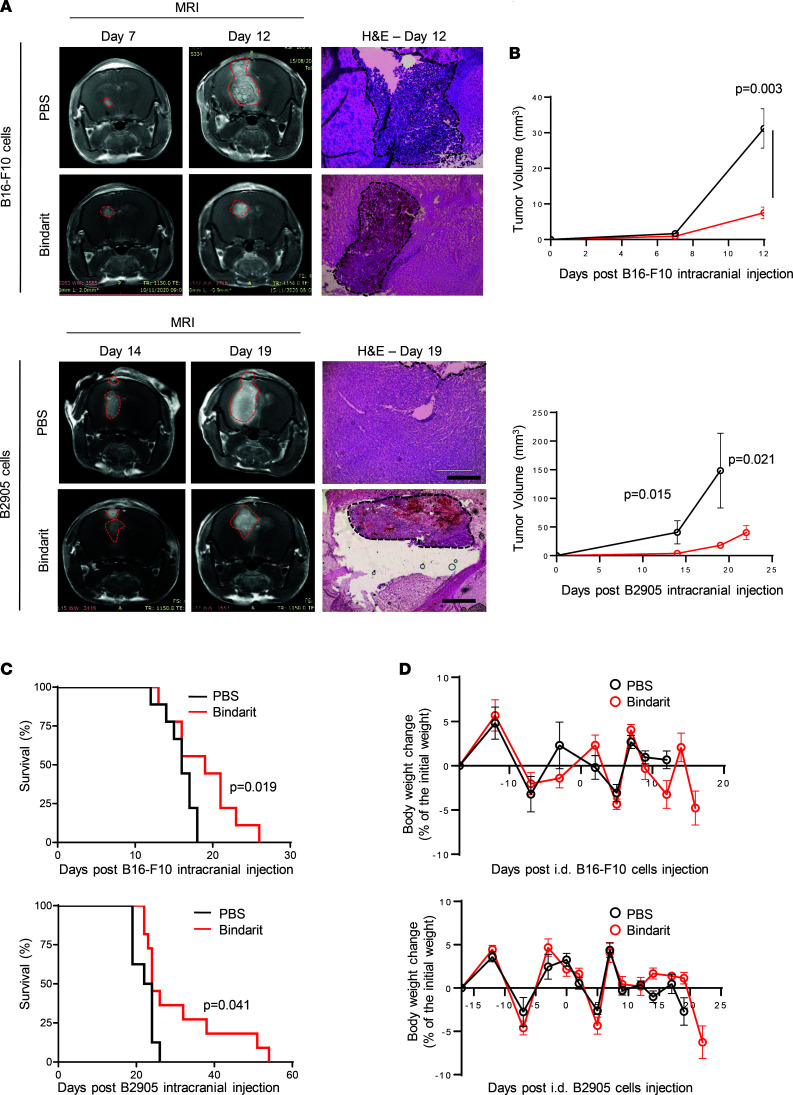
Bindarit-treated MBM results in a discrete although significant prolongation of the overall mouse survival. (**A**) Representative MRI scans and H&E staining, and (**B**) tumor size quantification of B16-F10 and B2905 MBM–bearing mice in PBS- and bindarit-treated group. B16-F10 MBM — mean ± SEM of *n* = 7 in bindarit-treated group and *n* = 8 in PBS-treated group. B2905 MBM — mean ± SEM of *n* = 7 in PBS- and bindarit-treated groups. Nonparametric Student’s *t* test. (**C**) Kaplan-Meier survival curve of B16-F10 MBM–bearing mice showed that bindarit prolonged survival. B16-F10 MBM — *n* = 8 in PBS-treated group, *n* = 7 in bindarit. B2905 MBM — *n* = 8 in PBS- and bindarit-treated group. Two-tailed *P* values from log-rank (Mantel-Cox). (**D**) Body weight change was monitored twice a week upon B16-F10 or B2905 tumor resection. Mean ± SEM of *n* = 8 in PBS-treated group, *n* = 7 in bindarit in B16-F10 MBM, and *n* = 8 B2905 MBM in PBS- and bindarit-treated groups.

To activate the host immune system prior to direct inoculation of melanoma cells in the brain, while mimicking as faithful as possible a more realistic condition found in human patient, MBM was generated following primary melanoma (PM) establishment and resection. Although limited by the fact that not all human patients will ultimately develop brain metastasis, the design of our in vivo models was carried out with the interventional purpose of evaluating the therapeutic efficacy of MCP-1 inhibition in MBM developing in the same location in the brain (Supplemental Figure 15A and Supplemental Figure 16A). The MBM models we generated differ in their (i) tumor growth kinetics, (ii) oncogenic driver mutation, and (iii) immunogenicity and therefore response to immunotherapy. The models selected bear either BRAF-V600E oncogene mutation (D4M.3A cells) (34) or PTEN-T131P onco-suppressor mutation (B16-F10 cells) (35), the RET oncogene mutation (Mel-ret cells) (36), or HGF/SF oncogene fusion + UVB radiation (B2905 cells) (37). MBM progression was monitored and MRI scan analysis demonstrated that treatment with bindarit resulted in 60%–80% inhibition of tumor growth in all 4 MBM models examined (Figure 4, A and B; Figure 5, A and B; Supplemental Figure 15D; and Supplemental Figure 16B), without causing any body weight loss (Supplemental Figure 15, B and C; Figure 5D; and Supplemental Figure 16C). Bindarit-treated MBM resulted in a discrete, although significant, prolongation of the overall mouse survival (Figure 5C). The infiltration of immune cells into MBM lesions was analyzed by flow cytometry (FACS) for the presence of T cell populations and myeloid-derived infiltrating cells (e.g., myeloid-derived suppressor cells, MDSCs). Enhanced presence of CD8^+^ and CD4^+^ T cells was found in bindarit-treated D4M.3A, B16-F10, or Mel-ret MBM lesions (Supplemental Figures 17 and 18). Moreover, low proliferative tumors (low Ki67 staining) and high infiltration of CD8^+^ T cells that were activated (high CD107 staining) and yet not exhausted (low PD-1 expression) were observed in the bindarit-treated group (Figure 4C, Supplemental Figure 15E, and Supplemental Figure 18). Bindarit inhibited the infiltration of suppressive CD4^+^ Tregs (Supplemental Figure 18), CD11b^+^ myeloid cells with inhibitory phenotype (MDSCs) (CD11b^+^Gr1^+^), and macrophages (CD11b^+^F4/80^+^) (Supplemental Figure 17), as well as Iba1^+^ microglia/macrophages’ recruitment, in treated MBM BME compared with those in the PBS-treated group (Figure 4C and Supplemental Figure 15E).

### Neutralization of the MCP-1/CCR2/CCR4 axis by knocking out CCR2/CCR4 recapitulates tumor growth and tumor landscape, as observed following pharmacological inhibition of MCP-1.

**Figure 6:**
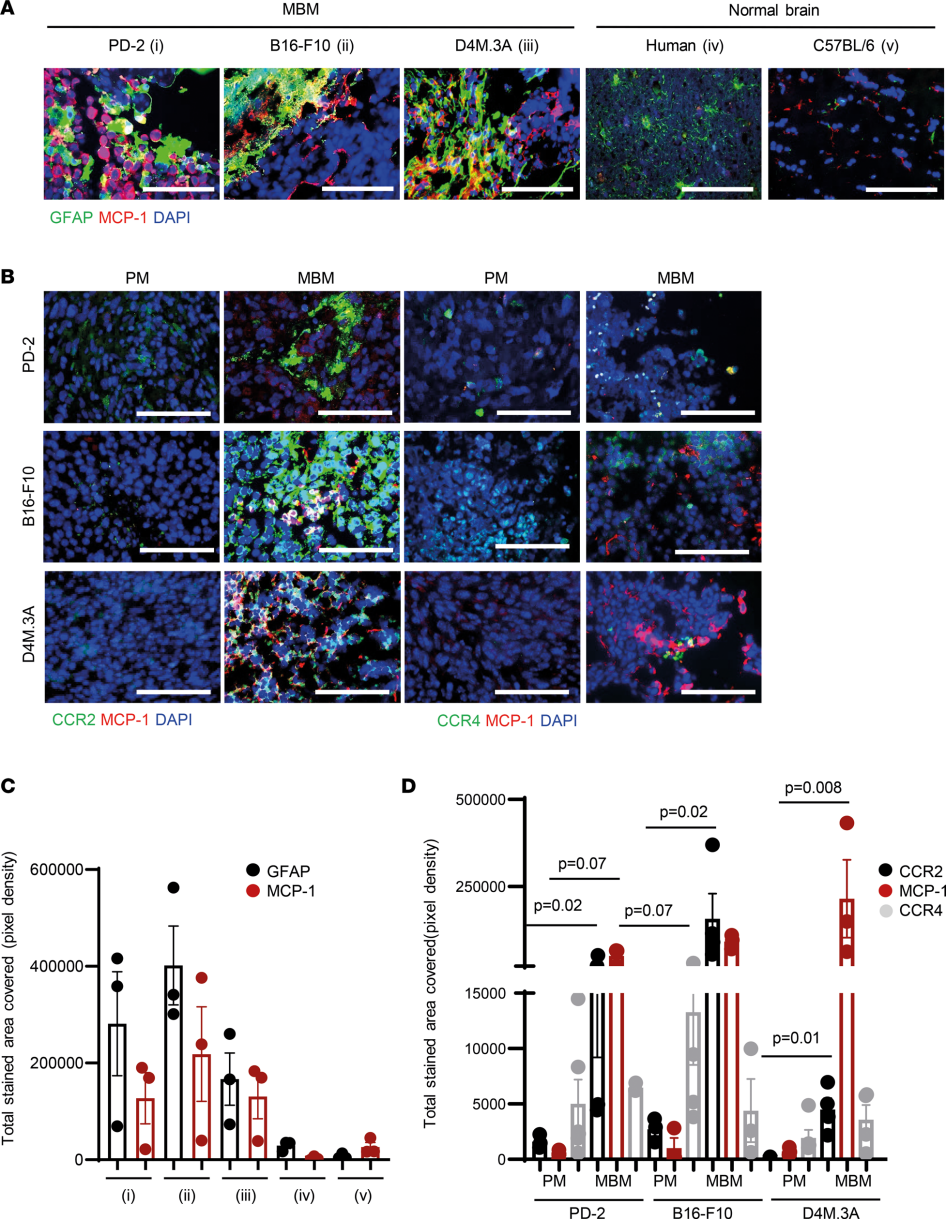
Astrocytes’ activation and MCP-1 secretion may trigger CCR2/CCR4 overexpression in MBM. (**A**) GFAP/MCP-1 immunostaining (green/red) in activated astrocytes within melanoma tumors and in normal brain of human and mouse. FFPE PD-2; cryosection of MBM of mouse models (intracardiac injection of mCherry-labeled D4M.3A cells or mCherry-labeled B16-F10 cells). Representative fields of *n* = 3 MBM per cell line. Nuclei are shown by DAPI staining (blue). Scale bar — 100 μm. Mean ± SD (*n* = 3–7 fields of *n* = 3 MBM per cell line). One-way ANOVA. (**B**) Representative fields of MCP-1/CCR2 and MCP-1/CCR4 staining in PD-2 and in mouse models of PM and MBM. Scale bar — 100 μm. Nuclei are shown by DAPI staining (blue). Mean ± SD *n* = 7–10 fields of *n* = 3 MBM per cell line. One-way ANOVA. (**C**) Quantification of **A** GFAP/MCP-1 immunostaining (green/red) in activated astrocytes within melanoma tumors and in normal brain of human and mouse. (**D**) Quantification of **B** in PD-2 and in mouse models of PM and MBM.

We showed the clinical relevance of targeting MCP-1 in MBM as high levels of MCP-1 were found in MBM samples of patients and mouse models (intracardially or intracranially inoculated D4M.3A of B16-F10 cells). Indeed, MCP-1 was highly expressed in melanoma brain tumors, including in astrocytes (confocal images and colocalization in *Z*-stack), whereas low basal expression was found in normal healthy brain samples from human and murine tissues (Figure 6, A and C; Supplemental Figure 19A; and Supplemental Video 1). Next, astrocyte-melanoma interactions, via MCP-1/CCR2 axis activation, were explored by comparing the expression of CCR2 in FFPE sections of human PM and MBM matched samples or in cryosections of murine PM and in MBM models. In PM, the expression of MCP-1 and CCR2 was poor, while several regions of positive double staining were found in the metastatic lesions (Figure 6, B and C, and Supplemental Figure 20A). As MCP-1 can also bind CCR4 (25), its expression level was assessed in human and murine mouse models as well. Significantly higher expression of all 3 genes — CCR2, CCR4, and MCP-1 — was found in MBM biopsies (from The Cancer Genome Atlas, TCGA) when compared with basal expression in healthy brain specimens of humans (from Genotype-Tissue Expression, GTex) and mice (Supplemental Figure 19B and Supplemental Figure 20B). CCR4 was expressed both in PM and in brain metastases, with the tendency to be highly expressed in MBM of PD and D4M.3A samples. CCR2 was detected mostly in MBM samples from patients, whereas its expression was almost absent in PM samples from those patients (Figure 6, B and D, and Supplemental Figure 20A).

**Figure 7:**
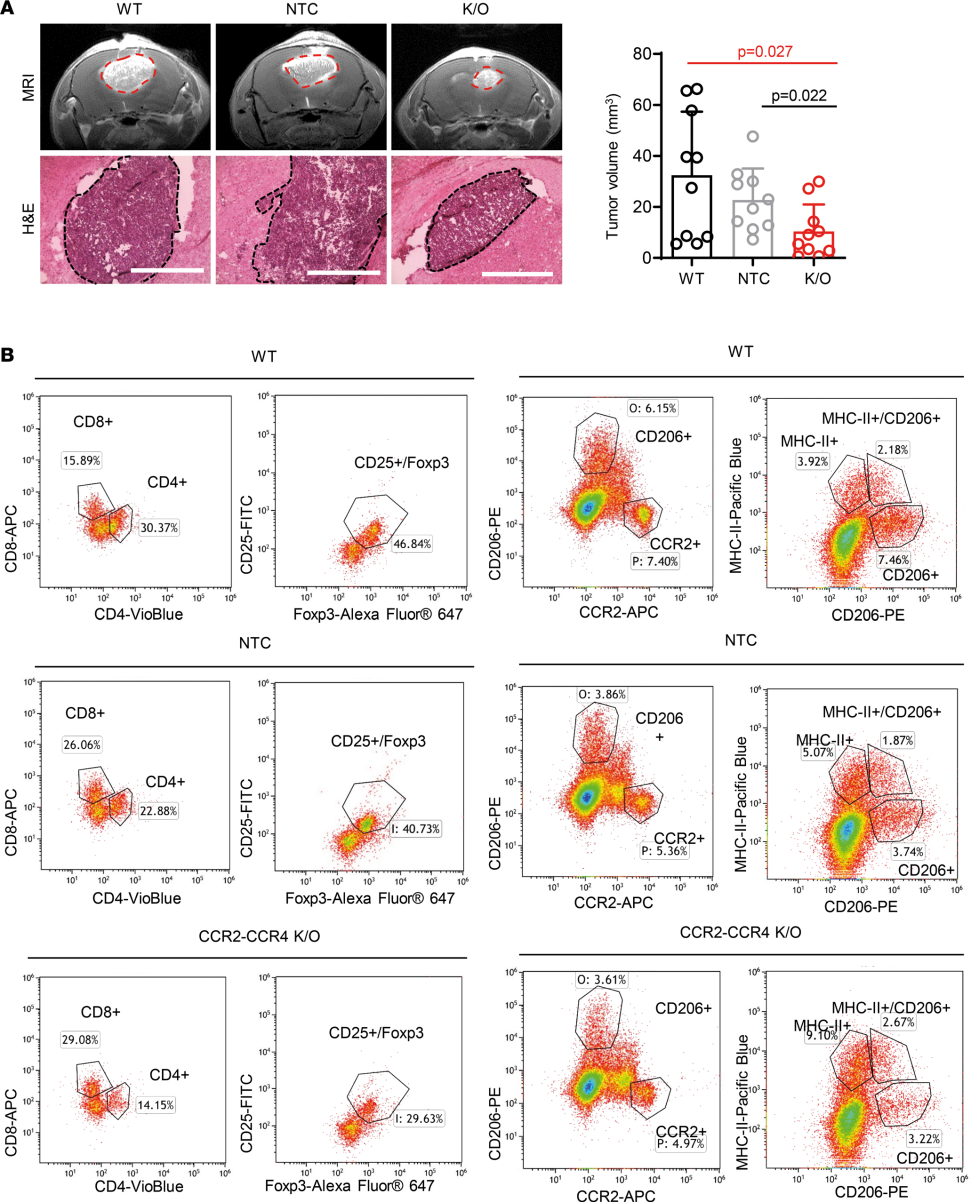
CCR2/CCR4 CRISPR/Cas9 K/O recapitulates tumor growth and tumor landscape as observed following pharmacological inhibition of MCP-1. (**A**) Tumor size and quantification of K/O WT and NTC, shown by its relative tumor volume quantification of MRI scans and H&E staining. Mean ± SEM of *n* = 10 mice per group. Ordinary 1-way ANOVA. (**B**) Immune cell infiltration into tumors (**A**) was analyzed by FACS. For each marker analyzed, the percentage of positive cells gated is presented. *n* = 2 mice per group.

Furthermore, to understand the clinical relevance of targeting CCR2 and CCR4 receptors expressed in MBM and support our results in matched samples of MBM and PM from human patients, we plotted the expression levels of CCR2 and CCR4 collected from Cancer Cell Line Encyclopedia/DepMap (CCLE/DepMap) data sets of melanoma cell lines from the Broad Institute, where skin cutaneous melanoma (SKCM) human biopsies were derived from primary or brain metastatic sites (38). Here, CCR2 expressed by melanoma derived from the brain was 7-fold higher compared with melanoma in the skin, whereas a 2-fold increase was observed for CCR4’s expression (Supplemental Figure 20C). Moreover, in vitro, we found that MCP-1 secreted from astrocytes or recombinant murine MCP-1 (rm-MCP-1) directly induced the expression of CCR2 in B16-F10 melanoma cells (Supplemental Figure 21A), otherwise poorly expressed in the absence of MCP-1 stimulus. These findings demonstrate the induced dependency of CCR2 expression in melanoma cells following MCP-1 stimulation and the consequent role of this activated pathway in support of tumor cell growth, invasion, and migration. Hence, with the intention of blocking the interaction of cancer cells expressing CCR2 (and CCR4) with MCP-1 secreted in MBM (from astrocytes and other sources of cells in the brain, including tumor cells), we knocked out CCR2 and CCR4 genes in melanoma cells, using the CRISPR/Cas9 system. A schematic representation of the experimental setting for the generation of CCR2 and CCR4 knockout (K/O) cells is presented in Supplemental Figure 21B. B16-F10 parental cells (WT) were sorted for CCR2/CCR4 expression (Supplemental Figure 21C). CRISPR/Cas9 and guide RNA (gRNA) targeting CCR2 and CCR4 were introduced through cell electroporation. CCR2^–^CCR4^–^ cells were then sorted (Supplemental Figure 21D). The efficacy of CCR2 and CCR4 K/O was validated through genomic DNA sequencing, resulting in 35% and 30% gene editing, respectively (Supplemental Figure 21E). As NC, B16-F10 cells were treated with CRISPR/Cas9 without a gRNA (NTC cells) and underwent sorting for CCR2^–^CCR4^–^. Having the NTC cells also sorted for CCR2^–^CCR4^–^ expression is an important control, as we expect this negative expression to be transient in NTC cells, in contrast to CCR2^–^CCR4^–^ gRNA-treated cells, where the negative expression should be stable. Finally, B16-F10 WT, NTC, and CCR2/CCR4 K/O MBM were generated following intracranial cell inoculation. On day 9, MRI scan analyses demonstrated that K/O melanoma tumors developed into less aggressive brain lesions (Figure 7A). Moreover, we observed a moderate increase in overall mouse survival (Supplemental Figure 22A). Histological analysis of double-K/O tumors presented decreased tumor-associated CCR2 and CCR4 expression and poor macrophage-associated (F4/80^+^ cells) CCR2^+^ expression. Moderate reduction in GFAP activation was observed along with reduced tumor proliferation (Ki67) and lower levels of Iba1^+^ microglia/macrophages (Supplemental Figure 22B). FACS analysis demonstrated higher infiltration of CD8^+^ T cells and reduced presence of CD4^+^CD25^+^Foxp3^+^ (Tregs) into double-K/O tumors (30%–40% reduction compared with NTC and WT) (Figure 7B). Furthermore, macrophages/microglia (CD11b^+^/F4/80^+^) in K/O tumors showed higher levels of the antigen-presenting marker MHC class II, and lower expression of immunosuppressive markers CD206 and CCR2, compared with WT and NTC control tumors (Figure 7B).

## Discussion

One of the major challenges for the treatment of metastatic melanoma is the development of tumor resistance against immunotherapies (39). This relies on melanoma cell ability to secrete immunosuppressive cytokines within the BME, upregulate inhibitory checkpoint molecules on T cells, and escape immune recognition (40–44). Astrocytes were found to secrete tumor survival factors and cooperate in the establishment of MBM and in the reinforcement of the protumorigenic niche (10, 45, 46). In this study, we investigated the reciprocal role of MCP-1/CCR2 axis interactions between astrocytes (and BME) and melanoma cells in supporting MBM progression, while establishing an immunosuppressive microenvironment. Using several human and murine melanoma cell lines, we demonstrated that astrocytes, even before melanoma-astrocyte interaction, secreted proinflammatory cytokines. Among them we found MCP-1, which attracted migrating melanoma cells. In addition, upon interaction with melanoma cells, astrocytes overexpressed *MCP-1* at the mRNA and protein levels, enhancing melanoma cell migration and invasion. Despite the fact that MCP-1 is secreted to some extent by other brain-resident cells (e.g., microglia and endothelial cells) (13), following melanoma cell interactions with each of the abovementioned BME cells, astrocytes oversecreted MCP-1 while interacting with melanoma cells, resulting in enhanced cell migration and proliferation. Similarly, melanoma and other types of cancer cells were shown to produce and oversecrete MCP-1 while interacting with the brain-resident cells, supporting and increasing the infiltration of immunosuppressive cell subtypes into the BME (17, 47). To inhibit MCP-1 oversecretion in the BME, pharmacological intervention was evaluated in vitro and in a syngeneic intracranially injected MBM mouse model. A small molecule inhibitor of MCP-1, bindarit, developed as an antiinflammatory agent (31), is currently in phase II clinical trials for the treatment of nonmalignant diseases, such as diabetic nephropathy (ClinicalTrials.gov NCT01109212) and coronary restenosis (ClinicalTrials.gov NCT01269242). In preclinical cancer studies, bindarit was shown to reduce local tumorigenesis of melanoma and breast and bone cancer, as well as to attenuate pain and tumor-associated macrophage–related (TAM-related) inflammation in tumors (48–50). Moreover, blocking MCP-1, via bindarit, inhibited lung metastasis formation of prostate tumor in a xenograft model (48, 51).

In our study, we showed that bindarit selectively inhibited MCP-1 in astrocytes, causing reduction of melanoma cell sprouting in 3D multicellular MBM spheroids, and melanoma cell migration in coculture with astrocytes and endothelial cells. The addition of recombinant MCP-1 to the astrocyte-melanoma coculture partially rescued the migration ability of melanoma cells. We speculate that the presence of cell contact while MCP-1 is inhibited may lead to the activation of several alternative mechanisms and pathways, and thus the addition of recombinant MCP-1 resulted in only partial, although significant (*P* = 0.0006), rescue of melanoma cell migration. These findings validate the selectivity of bindarit in inhibiting MCP-1 and demonstrate that MCP-1, though not exclusively, has a paramount role in melanoma and astrocyte interaction, facilitating tumor cell aggressiveness. Indeed, MCP-1 inhibition did not affect melanoma cells in the absence of astrocyte-melanoma interaction. All these findings suggest that (i) the secretion of MCP-1 from astrocytes occurs under activation stimuli (e.g., LPS), and it is enhanced under the influence of melanoma secreted factors and cell contact (coculture); (ii) the CM of astrocytes enriched in MCP-1 are sufficient to increase invasion and migration of melanoma cells; and (iii) bindarit efficiently blocks MCP-1 expression and secretion by astrocytes, while having no direct observable effects on the soluble form of MCP-1.

MCP-1–induced metastasis is attributed to the recruitment and polarization of MCP-1–secreting TAMs (52). Indeed, MCP-1 has an unfavorable effect on tumor prognosis due to the intratumor accumulation of immunosuppressive cell subtypes (e.g., TAM and immature MDSC) (53), reducing the potential cytotoxicity of CD8^+^ T cells (54). When we inhibited MCP-1 in 4 models of MBM, we showed higher activation (CD107^+^) yet not exhaustion (low PD-1^+^ staining) of CD8^+^ T cells; lower infiltration of Tregs, CD11b^+^Gr1^+^ population (MDSCs), and immunosuppressive (CD206) macrophages; and poor recruitment of Iba1^+^ microglia/macrophages into slow-growing tumors (defined by Ki67 staining). Indeed, we corroborated the evidence that in MBM a stronger antitumor activity correlated not only with a lower accumulation of tumor-infiltrating MDSCs and F4/80^+^ macrophages/microglia in favor of CD8^+^ T cells (55) but also with repolarization/activation of TAMs (56). As similarly observed in breast and prostate cancers and in a spontaneous model of lung metastasis, (48, 51, 57), alteration in the macrophage phenotype and activation of CD8^+^ T cells was obtained following MCP-1 neutralization (using monoclonal antibody or bindarit).

We demonstrated that melanoma cells gained the ability to overexpress CCR2 in response to MCP-1 oversecretion, enhancing their tumorigenic properties in vitro. We observed that melanoma cells with higher tropism to the brain were also more sensitive to MCP-1 inhibition both in vitro and in vivo. Indeed, we showed that MBM of patients and murine specimens is characterized by gained expression of CCR2 as opposed to that in primary lesions. A similar scenario in MBM was previously reported for CCR4 receptor (secondary MCP-1 receptor), found to be overexpressed by melanoma cells in advanced disease (25). Although CCR2 K/O in breast cancer cells reduced tumor cell growth and recruitment of antiinflammatory protumorigenic macrophages (58), attempts to neutralize MCP-1 (CNTO888) and CCR2 (MLN1202) led only to transient and unpredictable anticancer activities in preclinical and clinical studies (NCT01204996, NCT01015560) (59). To overcome the lack of therapeutic efficacy by either blocking MCP-1 or blocking CCR2 or CCR4 with pharmacological agents, we present a CRISPR/Cas9 double K/O in melanoma cells to target the activation of an alternative compensatory pathway activated by MCP-1 involved in MBM, such as MCP-1/CCR4. When we evaluated the tumor-infiltrating cell populations, double-K/O tumors presented fewer Iba1^+^-activated F4/80^+^ macrophages/microglia, lower secretion of MCP-1 in GFAP-activated astrocytes, and poor expression of CCR2/CCR4 receptors. In addition, infiltrating macrophages/microglia were primed toward a more proinflammatory/antitumorigenic phenotype as evidenced by the increased expression of the antigen-presenting marker (MHC II) and decreased expression of CD206 and CCR2 protumorigenic markers. In our CCR2/CCR4 double-K/O model, Foxp3^+^ Treg infiltration was reduced (60), while a meaningful infiltration of CD8^+^ T cells was observed in our CCR2/CCR4 double-K/O model, similar to what was obtained in a model of liver cancer following CCR2 K/O or neutralization (61).

Overall, our study presents 2 effective strategies to interfere with tumor-host interactions in MBM. We demonstrate that melanoma cells altered astrocyte secretome and evoked MCP-1 expression and secretion, which in turn induced CCR2 expression in melanoma cells, enhancing the in vitro tumorigenesis (proliferation, migration, and invasion) of melanoma cells, while the inhibition of MCP-1 or CCR2 (via bindarit or CRISPR/Cas9 system) rescued this phenotype in vitro and in vivo. Our strategy to inhibit the MCP-1/CCR2 axis, in addition to the potentially unique combination with CCR4 receptor K/O, remarkably delayed MBM progression, activated the BME toward anticancer and proinflammatory phenotypes, and promoted infiltration of CD8^+^ T cells, altering the melanoma-brain cell landscape.

**Figure 8:**
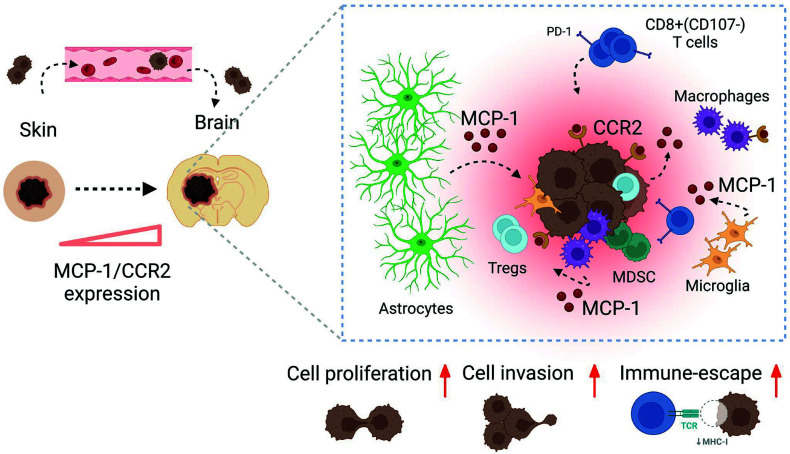
Summary of the proposed interaction mechanism of MCP-1/CCR2 in MBM. PM (in the skin) locally invades the tissue, intravasates into the vasculature, and spreads to distant organs, especially to the brain. In this scenario, cancer cells, BME cells, and in particular activated astrocytes, secrete MCP-1, and in return, melanoma cells express CCR2. TAMs/microglia, activated toward antiinflammatory/protumorigenic phenotypes together with Tregs, are recruited to the tumor site. CD8^+^ T cells are poorly activated (CD107) and express PD-1, which results in uncontrolled tumor dissemination. Created with BioRender.com.

Taken together, MCP-1/CCR2 inhibition represents a valuable strategy for educating immune cells toward anticancer phenotype in MBM. We propose that the MCP-1/CCR2 axis be considered an immune checkpoint regulator involved in melanoma-astrocyte and brain cell interactions that may drive and facilitate the establishment of an immunosuppressive microenvironment in which melanoma cells can take control, grow, and disseminate (Figure 8).

## Methods

All the reagents and antibodies used in the study, including catalog number, lot, dilution, and vendor, are described in the Supplemental Methods.

### Cell cultures

#### Human melanoma cell lines.

Metastatic melanoma 131/4-5B1 cells (provided by Robert Kerbel, Sunnybrook Health Sciences Centre, Toronto, Ontario, Canada) (62) were cultured in RPMI medium supplemented with 10% FBS, 100 IU/mL penicillin, 100 μg/mL streptomycin, 12.5 IU/mL nystatin, and 2 mM l-glutamine. Melanoma A375 cells (ATCC) were cultured in RPMI medium supplemented with 10% FBS, 100 IU/mL penicillin, 100 μg/mL streptomycin, 12.5 IU/mL nystatin, 2 mM l-glutamine, 1 mM sodium pyruvate, and 25 mM HEPES. Melanoma WM115 cells (ECACC) were cultured in MEM supplemented with 10% FBS, 100 IU/mL penicillin, 100 μg/mL streptomycin, 12.5 IU/mL nystatin, 2 mM l-glutamine, 1 mM sodium pyruvate, and 1× of MEM nonessential amino acids.

#### Murine melanoma cell lines.

Melanoma B16-F10 cells (ATCC) were cultured in DMEM supplemented with 10% FBS, 100 U/mL penicillin, 100 μg/mL streptomycin, 12.5 IU/mL nystatin, and 2 mM l-glutamine. Melanoma B2905 (provided by Glenn Merlino, National Cancer Institute, NIH, Bethesda, Maryland, USA) were grown in RPMI supplemented with 10% FBS, 100 U/mL penicillin, 100 μg/mL streptomycin, 12.5 IU/mL nystatin, 25 mM HEPES, and 2 mM l-glutamine. Melanoma Mel-ret cells (provided by Viktor Umansky, German Cancer Research Center, Heidelberg, Germany) were grown in RPMI supplemented with 10% FBS, 100 U/mL penicillin, 100 μg/mL streptomycin, 12.5 IU/mL nystatin, 1 mM sodium pyruvate, and 2 mM l-glutamine. Melanoma D4M.3A cells (provided by David W. Mullins, Geisel School of Medicine – Dartmouth, Hanover, New Hampshire, USA) (34) were grown in Advanced DMEM supplemented with 5% FBS, 100 IU/mL penicillin, 100 μg/mL streptomycin, 12.5 IU/mL nystatin, and 2 mM Glutamax. All melanoma cell lines were labeled with pQC-mCherry retroviral particles, as previously described (63).

#### Human brain-resident cells.

Human astrocytes (ScienceCell) were cultured in astrocyte medium (AM) supplemented with 2% FBS, 100 IU/mL penicillin, 100 μg/mL streptomycin, and 1% astrocyte growth supplements. Human microglia (ScienceCell) were cultured in microglia medium supplemented with 5% FBS, 100 IU/mL penicillin, 100 μg/mL streptomycin, and 1% microglia growth supplements. Human cortical microvessel endothelial cells (CMEC/D3) were cultured in EndoGRO-LS supplemented with 5 ng/mL rh-EGF, 50 μg/mL ascorbic acid, 10 mM l-glutamine, 1 μg/mL hydrocortisone hemisuccinate, 0.75 U/mL heparin sulfate, 2% FBS, 100 U/mL penicillin, 100 μg/mL streptomycin, 12.5 U/mL nystatin, and 1 ng/mL rh–basic FGF.

#### Murine brain-resident cells.

Freshly isolated murine astrocytes were cultured in AM supplemented with 2% FBS, 100 IU/mL penicillin, 100 μg/mL streptomycin, and 1% astrocyte growth supplements. Freshly isolated mBECs were grown in EndoGRO-LS.

Cells were routinely tested for mycoplasma contamination with a mycoplasma detection kit (Biological Industries). All the cell cultures were grown at 37°C in 5% CO_2_.

### Astrocyte CM

To generate AM enriched with secreted cytokines, half a million human astrocytes were grown in serum-free astrocyte medium (AM SFM) for 24 hours. One million freshly isolated murine astrocytes were incubated in AM SFM for 24 hours supplemented with 100 ng/mL LPS to stimulate MCP-1 expression (30).

### Cytokine array

Human or murine astrocytes and melanoma cells (131-4/5B1, A375, WM115, B16-F10, and D4M.3A) were grown for 24 hours in their own SFM. Astrocytes and melanoma cells were then incubated for 24 hours in melanoma and astrocyte CM, respectively. Twenty-four hours later, media were replaced by SFM for additional 24 hours. As NC we included melanoma cells and astrocytes grown in their own CM. Media were collected, filtered with 0.2 μm pore filter, concentrated (Amicon Ultra 15 centrifugal filter), and analyzed for cytokine secretion using Cytokine Array kit according to the protocol provided by the manufacturer.

To assess the selective inhibition MCP-1 via small molecule inhibitor (bindarit), human astrocytes were treated with 0.3 mM bindarit in AM SFM. Twenty-four hours later, the medium was collected, filtered with 0.2 μm pore filter, concentrated (Amicon Ultra 15 centrifugal filter), and analyzed for cytokines secretion using Cytokine Array Kit according to the protocol provided by the manufacturer.

### Drug preparation

For in vitro experiments, a stock solution of 100 mM bindarit was prepared in DMSO, and serial dilutions (0.001, 0.01, 0.1, 0.3, 0.5, 1 mM) were made using the culture medium. For in vivo experiments, 150 mg bindarit was dissolved in 0.5 M NaHCO_3_ and water, then lyophilized overnight. A stock of 100 mg/kg bindarit was prepared in PBS.

### MTT assay

Melanoma D4M.3A cells (750 cells/well), B16-F10 cells (750 cells/well), WM115 cells (2 × 10^3^ cells/well), and A375 cells (1 × 10^3^ cells/well), or brain-resident cells (astrocytes, microglia, CMEC/D3 — 2 × 10^3^ cells/well), were plated onto 96-well plates and incubated for 24 hours. Cells were then treated with bindarit in serial dilutions. Seventy-two hours following treatment, 30 μL of 3 mg/mL MTT solution in PBS was added to the medium for 5 hours. Absorbance of the dissolved formazan crystals (MilliporeSigma) (in DMSO) was measured at 570 nm using SpectraMax M5 plate reader (Molecular Devices). In addition, brain-resident cells (astrocytes, microglia, CMEC/D3 — 2 × 10^3^ cells/well) were exposed to 10 μg/mL anti–MCP-1 neutralizing antibody for 72 hours. MTT cell viability assay was used to determine the IC_50_ as previously described.

### Transwell migration assay

Melanoma cell migration was assessed by using a Transwell method as previously described (63). The details regarding the experimental conditions are presented in Supplemental Methods.

### ELISA

MCP-1 levels secreted by astrocytes were assessed by ELISA using the Human CCL2/MCP-1 Quantikine or the Murine CCL2/MCP-1 Quantikine. The details regarding the experimental conditions are presented in Supplemental Methods.

### Multicellular tumor spheroids

Multicellular tumor spheroids of melanoma cells and astrocytes were prepared using the hanging-drop method as previously shown (64). The details regarding the experimental conditions are presented in Supplemental Methods.

### Western blot

To assess the level of NF-κB pathway activation in the presence of bindarit, human astrocytes were exposed for 2 hours to AM SFM supplemented with 0.3 mM bindarit. Astrocytes were then washed and exposed to B16-F10 CM with or without fresh addition of bindarit for 1 hour and 30 minutes. Cells were subsequently lysed in RIPA buffer supplemented with fresh protease and phosphatase inhibitors (Invitrogen, Thermo Fisher Scientific). Cell lysates were analyzed by SDS-PAGE. Proteins were then transferred on a nitrocellulose membrane and blocked with 5% BSA in Tris-HCl buffer with 1 % Tween-20. Anti–p-p65 and anti–total p65 antibodies were incubated with the nitrocellulose membrane overnight at 4°C. Anti-vinculin antibody (as loading control) and HRP-conjugated anti-rabbit secondary antibody were incubated for 1 hour at room temperature. SuperSignal West Pico PLUS chemiluminescent substrate was added prior to membrane development using iBright 1500 (Life Technologies, Thermo Fisher Scientific). Pixel densities of the protein bands were quantified using ImageJ software (NIH).

### Flow cytometry

For flow cytometry assays, cells were harvested and washed with FACS buffer. For brain-resident basal and melanoma-induced expression of MCP-1, astrocytes (1 × 10^5^ cells/6-well plate), microglia (0.5 × 10^5^ cells/6-well plate), or CMEC/D3 (2 × 10^5^ cells/6-well plate) were grown in monoculture or in coculture with WM115 (1 × 10^5^ cells/6-well plate) melanoma cells for 24 hours in SFM. Cells were harvested and incubated with anti–MCP-1 antibody, followed by anti-mouse Alexa Fluor 488 secondary antibody. For melanoma basal expression of CCR2 and CCR4, B16-F10 cells (5 × 10^5^ cells/10 cm^2^ dish) were grown in melanoma medium for 24 hours. Cells were incubated with APC–anti-CCR2 and PE–anti-CCR4 antibodies. For immune infiltration assessment, cells from freshly isolated D4M.3A MBM tumors were divided into 2 panels: (a) T cell panel: cells were incubated with FITC-labeled anti-CD3, APC-labeled anti-CD8, and VioBlue anti-CD4 antibodies; (b) MDSC and macrophage panel: cells were incubated with FITC–anti-CD11c, APC-Cy7–anti-CD11b, APC–anti–Gr-1, or FITC–anti-F4/80. For immune infiltration assessment, cells from freshly isolated B16-F10 MBM tumors knocked out for CCR2 and CCR4 were divided into 2 panels: (c) Treg panel: cells were incubated with PerCP–anti-CD3, VioBlue anti-CD4, FITC–anti-CD25, and Alexa Fluor 647 anti-Foxp3 following cell membrane fixation and permeabilization; (d) macrophage activation panel: cells were incubated with PE-Vio770–anti-CD11b, FITC–anti-F4/80, VioBlue anti–MHC II, PE–anti-CD206, and APC–anti-CCR2. For immune infiltration assessment, cells from freshly isolated B16-F10 and Mel-ret MBM tumors were divided into 2 panels: (e) T cell activation panel: cells were incubated with VioBlue–anti-CD3, APC–anti-CD8, Alexa Fluor 488–anti-CD107; (f) Treg panel: cells were incubated with PerCP–anti-CD3, VioBlue anti-CD4, FITC–anti-CD25, and Alexa Fluor 647–anti-Foxp3 following cell membrane fixation and permeabilization. Cells were incubated for 30 minutes at room temperature. Single-stained cells for each antibody and a pool of the corresponding isotype control were used as negative staining controls. Splenocytes isolated from the same animals were used as positive controls to discriminate for immune cell populations. Cells were then run and analyzed using CytoFlex cytometer and CytoExpert analysis software or Attune NxT cytometer and Kaluza analysis 1.3 software.

### Animal models

#### PM tumor.

To generate PM before MBM, D4M.3A and B2905 cells — 0.5 × 10^6^ cells/100 μL — or B16-F10 and Mel-ret — 0.1 × 10^6^ cells/100 μL — were inoculated intradermally in immunocompetent C57BL/6 mice (Envigo, CRS). Then, primary lesions were resected once they reached a volume of 70–100 mm^3^.

#### Spontaneous MBM tumor.

mCherry-labeled 131/4-5B1 cells (1 × 10^6^ cells/100 μL), WM115 cells (2 × 10^5^ cells/100 μL), B2905 cells (1 × 10^6^ cells/100 μL), or D4M.3A cells (1 × 10^6^ cells/100 μL) were injected by ultrasound-guided inoculation to 6- to 8-week-old male immunocompromised SCID mice (Envigo, CRS) or immunocompetent C57BL/6 mice. We performed 4.7 T/1 H MRI (MR Solutions) twice a week for the following 4–6 weeks after melanoma cell inoculation. Further details on the mouse models used to generate MBM are presented in Supplemental Methods.

#### Melanoma brain tumor.

For drug efficacy in intervention studies, B16-F10 cells (1.5 × 10^4^ cells/2 μL) were intracranially inoculated stereotactically to the striatum (2 mm left from the bregma and 3.5 mm depth) of 8- to 10-week-old C57BL/6 male mice. Three days following cells’ inoculation, mice were treated i.v. with 100 mg/kg bindarit or PBS QOD. On days 10 and 13 after melanoma cells’ inoculation, mice were euthanized and immediately perfused with 4% paraformaldehyde (PFA) in PBS. Brains were then harvested for further immunostaining analysis. For functional evaluation of CRISPR/Cas9 K/O engineered cells, B16-F10 CCR2/CCR4-K/O cells, NTC cells, or WT cells (1.5 × 10^4^ cells/2 μL) were intracranially inoculated stereotactically into the striatum of 7-week-old male C57BL/6 mice. Mice were euthanized and brains were then harvested for further immunostaining and flow cytometry analysis. For drug efficacy in a prevention study, 8-week-old male C57BL/6 mice were intradermally injected with D4M.3A, B16-F10, and B2905 melanoma cells (5 × 10^5^ cells/100 μL). Tumor growth was measured twice a week using a caliper, and once tumors reached a size of 70–100 mm^3^, they were resected. Mice were immediately administered i.v. with 100 mg/kg bindarit or PBS QOD. Then after tumor resection, B16-F10 and ret-Mel (1.5 × 10^4^ cells/2 μL) cells, and D4M.3A and B2905 cells (5 × 10^4^ cells/2 μL) cells, were inoculated into the striatum of the same mice (previously bearing the primary tumor). Mouse body weight was monitored twice a week, and tumor growth was measured at least 2 times using 4.7 T/1 H MRI. On day 8, 12, 16, or 20 mice were euthanized, and brains were harvested for further immunostaining and flow cytometry analysis.

### Human FFPE specimens

FFPE melanoma samples (primary and brain metastasis, *n* = 40) were obtained from Sheba Medical Center following informed consent. The manipulation of the human samples for immunostaining was accepted by the ethics committees of Tel Aviv University and Sheba Medical Center, under an approved IRB (5727-18-SMC) protocol. A total of 6 primary melanomas and brain metastasis pairs were collected. Healthy human brain samples were collected by Thomas Hyde at the Lieber Institute for Brain Development as described under an approved IRB protocol, 90-M-0142.

### Frozen O.C.T. tissue fixation and preparation for IHC 

Tumor-bearing mice were anesthetized using ketamine (100 mg/kg) and xylazine (12 mg/kg) and perfused with 4% PFA in PBS. Mouse brains were resected, then incubated with 4% PFA for 4 hours, followed by 0.5 M of d-sucrose for 1 hour and 1 M d-sucrose overnight. Tissues were then embedded in O.C.T. on dry ice and stored at –80°C.

### Tissue staining

For immunostaining of brain tumors, frozen O.C.T.-embedded tissues were cryosectioned into 5 μm sections. Slides were fixed and permeabilized in acetone for 20 minutes at room temperature. Briefly, slides were incubated with goat serum (10% goat serum in PBS 1× + 0.02% Tween-20) for 30 minutes. Slides were stained for morphology by H&E or immunostained for anti-mouse/human MCP-1, anti-mouse/human GFAP, anti-mouse/human CCR2, anti-mouse/human CCR4, anti-mouse/human Iba1, anti-mouse CD31, anti-mouse/human Ki67, anti-mouse CD8a, anti-mouse F4/80, anti-mouse IL-6, anti-mouse/human PD-1, anti-mouse PD-L1, and anti-mouse CD206. After 1 hour of incubation, slides were incubated with secondary antibodies for an additional 1 hour: goat anti-rabbit Alexa Fluor 488 for GFAP, CCR2, and Ki67; goat anti-mouse Alexa Fluor 647 for MCP-1, NK-1.1, and CD206 markers; goat anti-rabbit Alexa Fluor 647 for PD-1, CCR4, and Ki67 markers; and goat anti-rat Fluor 488 for CD31, CD8a, F4/80, IL-6, or PD-L1 markers. Nuclei were counterstained using Hoechst solution. The stained tissues were then fixed and mounted on a glass microscope slide using ProLong Gold antifade mounting. Fluorescence and bright-field images were captured using a fluorescence and bright-field illumination microscope (Evos FL Auto, Life Technologies, Thermo Fisher Scientific) at 40× and 10× original magnifications, respectively.

### Data availability

For the gene expression comparison in healthy versus SKCM brain metastasis, the DESeq2-normalized and batch-corrected expression counts of healthy brain biopsies and SKCM brain metastasis were downloaded from the UCSC Xena browser using the following link: https://xenabrowser.net/datapages/?dataset=TcgaTargetGtex_RSEM_Hugo_norm_count&host=https%3A%2F%2Ftoil.xenahubs.net&removeHub=https%3A%2F%2Fxena.treehouse.gi.ucsc.edu%3A443 (set ID TcgaTargetGtex_RSEM_Hugo_norm_count). Their respective phenotype information was also downloaded from the same website using the following link: https://xenabrowser.net/datapages/?dataset=TcgaTargetGTEX_phenotype.txt&host=https%3A%2F%2Ftoil.xenahubs.net&removeHub=https%3A%2F%2Fxena.treehouse.gi.ucsc.edu%3A443 (set ID TcgaTargetGTEX_phenotype.txt). The transcript to gene mapping was downloaded from BioMart: https://useast.ensembl.org/info/data/biomart/index.html

### Statistics

Data are expressed as mean ± SD for in vitro assays and as mean ± SEM for in vivo assays. Statistical analyses were performed with Student’s 2-tailed *t* test or 1-way or 2-way ANOVA. Statistical significance in mouse overall survival was determined by the log-rank test using GraphPad Prism software (GraphPad Software Inc.). *P* < 0.05 was considered statistically significant.

### Study approval

All animal procedures were performed in compliance with Tel Aviv University and approved by the Institutional Animal Care and Use Committee (protocol no. 01-16-054 and no. 01-21-006). Postmortem human brain tissue was obtained by autopsy from the Offices of the Chief Medical Examiner of the District of Columbia, and of the Commonwealth of Virginia, Northern District, all with written informed consent from the legal next of kin (protocol 90-M-0142 approved by the National Institute of Mental Health/NIH IRB). Additional postmortem human brain tissue samples were provided by the National Institute of Child Health and Human Development Brain and Tissue Bank for Developmental Disorders (http://www.BTBank.org) under contracts NO1-HD-4-3368 and NO1-HD-4-3383. The IRBs of the University of Maryland and State of Maryland Department of Health and Mental Hygiene, both in Baltimore, Maryland (USA), approved the protocol, and the tissue was donated to the Lieber Institute for Brain Development under the terms of a Material Transfer Agreement. Clinical characterization, diagnoses, and macro- and microscopic neuropathological examinations were performed on all samples using a standardized paradigm, and patients with evidence of macro- or microscopic neuropathology were excluded. Details of tissue acquisition, handling, processing, dissection, clinical characterization, diagnoses, neuropathological examinations, RNA extraction, and quality control measures were as described previously (65). The Brain and Tissue Bank (https://www.medschool.umaryland.edu/btbank/) cases were handled in a similar fashion.

## Author contributions

RSF and SP conceived the study; SP, AS, EY, PO, DBS, S Soffer, AB, ADN, AI, EP, AR, and IS carried out experimental procedures; SP, EY, DBS; ER, S Sinha, TW, AM, and RSF performed formal analysis and investigation; IB, HB, TMH, and HFF acquired resources; SP, AS, EY, RSF, PO, and DBS wrote the manuscript; and all authors reviewed the manuscript and provided comments. RSF acquired funding for and supervised the study.

## Supplementary Material

Supplemental data

Supplemental video 1

## Figures and Tables

**Table 1:**
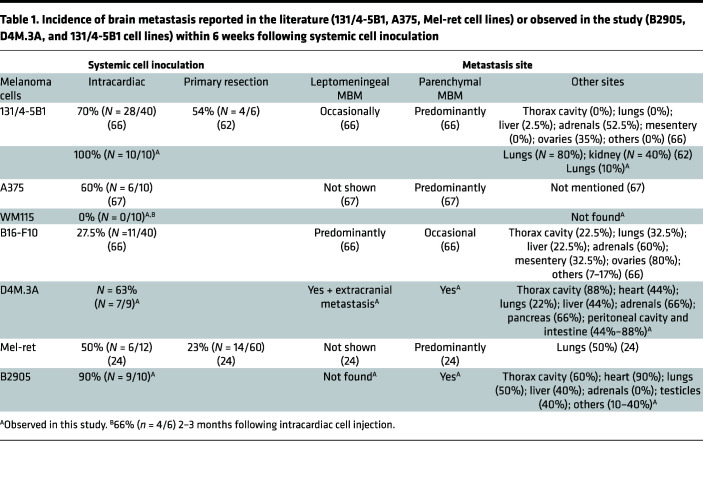
Incidence of brain metastasis reported in the literature (131/4-5B1, A375, Mel-ret cell lines) or observed in the study (B2905, D4M.3A, and 131/4-5B1 cell lines) within 6 weeks following systemic cell inoculation

**Table 2:**
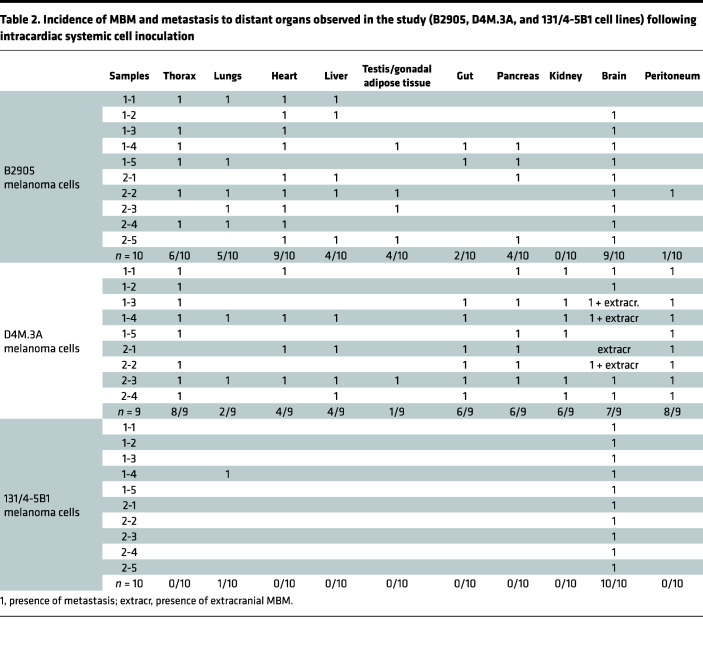
Incidence of MBM and metastasis to distant organs observed in the study (B2905, D4M.3A, and 131/4-5B1 cell lines) following intracardiac systemic cell inoculation

## Version 1 08/18/2022

In-Press Preview

## Version 2 09/08/2022

Electronic publication

## Version 3 10/05/2022

Updated affiliation numbers
