# Supplementary material for: MCP-1/CCR2 axis inhibition sensitizes the brain microenvironment against melanoma brain metastasis progression
Source: JCI Insight. 2022 Sep 8;7(17):e154804. doi: 10.1172/jci.insight.154804 (PMC9536270; doi:10.1172/jci.insight.154804)
Supplement: Supplemental data [file jciinsight-7-154804-s253.pdf]

Supplemental Information

Materials. DMEM, Advanced DMEM, RPMI, MEM media, fetal bovine serum (FBS), and Glutamax were purchased from Gibco (USA). Astrocytes medium (AM – Cat. No. 1801) and astrocytes growth supplements were purchased from ScienceCell (USA). Microglia medium (MM – Cat. No. 1901) and microglia growth supplements were purchased from ScienceCell (USA). EndoGRO Basal Medium and supplements kit (SCME001 kit), Hemacolor®Rapid staining (Cat. No. 1119562500/1119572500) were purchased from Merck (Darmstadt, Germany). Dulbecco's phosphate buffer saline (PBS), L-glutamine, penicillin, streptomycin, sodium pyruvate, HEPES, MEM non-essential amino acids (NAA) solution, trypsin mycoplasma detection kit, EZ-RNA II total RNA isolation kit and fibronectin (1 mg/ml; Dilution: 1:100) were purchased from Biological Industries Ltd. (Kibbutz Beit HaEmek, Israel). Percoll solution (Cat. No. p4937) lipopolysaccharide (LPS; Cat. No. L8274), 3-(4,5-dimethylthiazol-2-yl)-2,5-diphenyltetrazolinium bromide (MTT; Cat. No. M2128), β-mercaptoethanol (Cat. No.M6250), latex beads for phagocytosis assays (Cat. No. L4655), methyl cellulose (Cat. No. M0512), EDTA (Cat. No. E5134), Triton-100x (Cat. No. 9002931; Lot. No. 033K01501), Dextran, from *Leuconostoc spp.* (Cat. No.31390; Lot. 20509147), Tween-20 (Cat. P1379; Lot. No. SLCB6677), Trizma® base (Cat. No. T1503; Lot. No. SLCD9753), acrylamide/bis-acrylamide, 40% solution (Cat. No. A7802; Lot. No. SLBR6765V), formamide (Cat. No. F9037), Evans Blue (Cat. No. 46160; Lot. No. 1185693), and all other chemical reagents, including salts and solvents, were purchased from Sigma-Aldrich (Rehovot, Israel). D-(+)-Sucrose (Cat. No. 001922059100), and Glycine (Cat. No. 000713239100; Lot. No. 1303611), and acetone (Cat. No. 000103020500; Lot. No. 1318371) were purchase from Bio-Labs ltd (Jerusalem, Israel). Milli-Q water was prepared using a Millipore water purification system. Amicon Ultra Centrifugal Filters with 3K molecular weight cut-off (Cat. No. UFC900324), bovine albumin serum (BSA), and sodium dodecyl sulfate (SDS) (Cat. No. 428018; Lot. No. 2956240) were purchased from Merck Millipore (Burlington, Massachusetts, USA). CellEvent™ Caspase-3/7 Green ReadyProbes™ Reagent (Cat. R37111) was purchased from Thermo-Fisher Scientific (Waltham, Massachusetts, USA). ImageLock tissue culture plate (Cat. No. 4379) were purchased from Sartorious (Göttingen, Germany). Sodium azide (Cat. No. 14314) was purchased from Alfa Aesar (Thermo Fisher Scientific, United Kingdom). SuperSignal™ West Pico Plus chemiluminescent substrate (Cat. No.

34580 Lot. No. UL293523), and Neon™ transfection system kit (Cat. No. MPK1025) were purchased from Thermo Scientific (Rockford, Illinois, USA). Quick DNA miniprep kit (Cat. No. D3024) was purchased from Zymo Research (Irvine, California, USA). PrimeSTAR MAX (Cat. No. R045B) was purchased from Takara Bio (Mountain View, California, USA). T7 Endonuclease 1 (Cat. No. M0302S) was purchased from New England Biolabs (United Kingdom). The qScript™ cDNA Synthesis Kit was purchased from Quantabio (Beverly, Massachusetts, USA). Fast SYBR TM green Master Mix was purchased from Applied Biosystems (California, USA). Collagenase IV, Dispase II (neutral protease) and DNase I, were purchased from Worthington Biochemical Corporation (New Jersey, USA). Rat tail collagen type I, growth factors-reduced (GFR) Matrigel®, 70 µm nylon strainer, and Transwell® permeable support (Cat. No. 3422) were purchased from Corning® (Glendale, Arizona, USA). RBC lysis solution (Cat. No. 420301) anti-mouse/rat/human MCP-1 neutralizing antibody (Cat. No. 505912; Lot. No. B296185; Clone 2H5) were purchased from BioLegend (San Diego, California, USA). MACS MS magnetic columns for cell separation (Cat. No. 130-042-201), CD11b MicroBeads (Cat. No. 130-093-634), and CD31 MicroBeads (Cat. No. 130-097-418) for cell isolation were purchased from Miltenyi Biotec (Bergisch Gladbach, Germany). 2-[(1-Benzyl-1H-indazol-3-yl)methoxy]-2-methylpropanoic (bindarit; Cat No. 130641-38-2) was purchased Angene Chemical (United Kingdom). Human MCP-1 ELISA kit (Cat. No. DCP00), Mouse MCP-1 ELISA kit (Cat. No. MJE00B) Mouse XL Cytokine Array Kit (Cat. No. ARY028), Human Cytokine Array kit (Cat. No. ARY005B), and hr-bFGF were purchased from R&D Systems (Minneapolis, Minnesota, USA). Recombinant human MCP-1 (rh MCP-1) (Cat. No. 300-04; Lot. No. 090831), recombinant murine MCP-1/JE (rm MCP-1) (Cat. 250-10; Lot. No. 0510126), rabbit anti-murine JE/MCP-1 (Cat. No. 500-P113; Lot. No. 0608M126RB), rabbit anti-murine KC (CXCL1) (Cat. No. 500-P115; Lot. No. 0909M127RB), rabbit anti-murine RANTES (Cat. No. 500-P118; Lot. No. 0406M124RB), rabbit anti-murine MIP-2 (CXCL2) (Cat. No. 500-P130; Lot. No. 091CY152RB) neutralizing antibodies were purchased from PeproTech (Rehovot, Israel). Anti-mouse SERPIN E1 neutralizing antibody (Cat. No. AM26216PU-N; Lot. No. 27379M0819-A) was purchased from Origene (OriGene Tech. GmbH, Herford, Germany). ProLong® Gold mounting (Cat. No. P36934) and Hoechst 33342 (Cat. No. H3570) were purchased from Invitrogen (Carlsbad, California, USA). Optimal Cutting Temperature (O.C.T.) compound (Cat. No. 4585) was purchased from Scigen

Scientific (Gardena, California, USA). Normal goat serum (Cat. No. OORA01661; Lot. No. 28653) was purchased from Aviva Systems Biology Corporation (San Diego, California, USA). Paraformaldehyde (PFA) 16% solution (Cat. No. 15710) was purchased from Electron Microscopy Sciences (Hatfield, Pennsylvania, USA). Mayer's Hematoxylin solution (Cat. No. 05-06002) and Eosin Y solution (Cat. No. 05-10002) were purchased from Bio-Optica (Milano, Italy). Primers and siRNA duplex, Alt-R spCas9 Nuclease V3 Cat. No. 1081059), and Alt-R CRISPR-Cas9 sgRNA were purchased from IDT (Jerusalem, Israel). Polyethyleneimine (PEI) transfecting agent was purchased from Polyplus transfection® (Illkirch, France). Plasmids: mCherry was subcloned by our group into the pQCXIP vector (Clontech, USA) as previously described (*1*). Protease/phosphatase inhibitor (100x) (Cat. No. 5872S; Lot. No.19) was purchased from Cell Signaling Technology® (Massachusetts, USA). Magnetol, Gd-DTPA was purchased from Soreq M.R.C. Radiopharmaceuticals (Israel). For animal studies 6-10 week-old male C57BL/6 mice, and 6–8 weeks old male SCID mice were purchased from Envigo CRS (Nes Ziona, Israel).

Antibodies: Primary immunostaining antibodies. Mouse anti-mouse/human MCP-1 (Cat. No. NBP2-22115; Lot. No. B-1; Dilution 1:100), Rabbit anti-mouse/human CCR2 (Cat. No. NBP2-67700; Cat. No. HM0909; Dilution 1:100), Rabbit anti-mouse/human Iba1 (Cat. No. NBP2-19019; Lot. No. 41556; Dilution: 1:200), rabbit anti-mouse/human Ki-67 (Cat. No. NB500-170; Lot. No. G15; Dilution 1:50), Rat anti-mouse F4/80 (BM8, Cat. NBP1-60140; Lot. No. 28094M1219-A; Dilution 1:50 dilution), Rabbit anti-mouse CCR4 (Cat. No. NBP1-86584; Lot. No. B96904) were purchased from Novus (Colorado, USA). Rat anti-mouse CD274 (B7-H1, PD-L1) (Cat. No. 124318; Lot No. B276997; Clone 10F.9G2; Dilution 1:100) was purchased from BioLegend (San Diego, California, USA). Rat anti-mouse CD31 (Cat. No. 550272; Lot. No. 6273859; Dilution 1:25) was purchased from BD Biosciences (Franklin Lakes, NJ, USA). Rat anti-mouse/human CD8 (Cat. No. 14-0808-82; Lot. No. 2003225; Clone 4SMIS; Dilution 1:50) was purchased from eBioscience (San Diego, California, USA). Mouse anti-mouse CD206 (Cat. No. 60143-1-Ig; Lot. No. 10004170; Dilution 1:200), Rabbit anti-mouse/human PD1/CD279 (Cat. No. 60143-1-Ig; Lot. No. 10004170) were purchased from Proteintech (Illinois, USA). Rabbit anti-mouse/human GFAP (Cat. No. Dilution 1: 500) was purchased from Dako (Denmark). Rabbit anti-mouse/human CCR4 (Cat. No.

PA5-99885; Lot. No. VE2987252; Dilution 1: 100) was purchased from Invitrogen (Carlsbad, California, USA). Rat anti-mouse IL-6 (Cat. No. ab191194; Lot. No. GR3186600-3; Clone MP5-20F3; Dilution 1:100) was purchased from Abcam (Cambridge, United Kingdom).

Secondary immunostaining antibodies: Goat anti-mouse Alexa Fluor® 647 (Cat. No. ab15115; Lot. No. GR309891-3; Dilution 1:300), goat anti-rabbit Alexa Fluor® 488 (Cat. No. ab150077; Lot No. GR315933-2; Dilution 1:300), and goat anti-rabbit Alexa Fluor® 647 (Cat. No. Ab150079; Lot. No. Gr3176223-2; Dilution 1:300) were purchased from Abcam (Cambridge, United Kingdom). Goat anti-rat Alexa Fluor® 488 (Cat 112-545-068; Lot. No. 143654; Dilution 1:300) and goat anti-rat Alexa Fluor® 647 (Cat. No. 112-605-003; Lot No. 137652; Dilution 1:300) were purchased from Jackson ImmunoResearch Laboratories, Inc. (West Grove, Pennsylvania, USA). **Flow cytometry antibodies.** Anti-mouse CCR2-APC (Cat. No. 150628; Lot. No. B294911 Clone SA203G11; Dilution 1:100), rat IgG2a, κ isotype control-APC (Cat. No. 150628; Lot. No. B294911 Clone SA203G11; Dilution 1:100), anti-mouse FoxP3-Alexa Fluor® 647 (Cat. No. 126408; Lot. No. B264076; Clone MF-14; Dilution 1:50), rat IgG2b, κ Isotype control-Alexa Fluor® 647 (Cat. No. 400626; Lot. No. B243822; Clone RTK4530; Dilution 1:50), anti-mouse CCR4-PE (Cat. No. 131204; Lot. No. B224241; Clone 2G12; Dilution 1:100), rat IgG2b, κ isotype control-PE (Cat. No. 400608; Lot. No. B220932; Clone RTK4530; Dilution 1:100), anti-mouse CD206-PE (Cat. No. 141706; Lot No. B280038; Clone C068C2; Dilution 1:100), mouse IgG1, κ isotype control-PE (Cat. No. 400112; Lot. No. B291605; Clone MOPC-21; Dilution 1:100), anti-mouse CD25-FITC (Cat. No. 101907; Lot. No. B276319; Clone 3C7; Dilution 1:50), rat IgG2b, κ isotype-control FITC (Cat. No. 400633; Lot. No. B210159; Clone RTK4530, Dilution 1:50), anti-mouse CD11c FITC (Cat. No.117305; Lot. No. B244373; Clone N418, Dilution 1:100), Armenian hamster IgG isotype control-FITC (Cat. No. 400905; Lot. No. B256163; Clone HTK888, Dilution 1:100), anti-mouse CD107a (LAMP-1)-Alexa Fluor®488 (Cat. No. 121608; Lot. No. B272833; Clone 1D4B; Dilution 1:100), rat IgG2a, κ isotype control-Alexa Fluor®488 (Cat. No. 400525; Lot. No. B228070; Clone RTK2758, Dilution 1:100), anti-mouse Ly-6G/Ly-6C (Gr1)-APC (Cat. No. 108411; Lot. No. B262854; Clone RB6-8C5, Dilution 1:100), rat IgG2b, κ isotype control-APC (Cat. No. 400611; Lot. No. B261320; Clone RTK4530, Dilution 1:100), anti-mouse/human CD11b-APC/Cyanine7 (Cat. No. 101225; Lot. No. B273856; Clone M1/70, Dilution 1:100), rat IgG2b, κ isotype control-APC/Cyanine7 (Cat. No. 400623; Lot.

No. B248744; Clone RTK4530, Dilution 1:100), anti-mouse CD3ε PerCP/Cyanine5.5 (Cat. No. 100328; Lot. No. B281044; Clone 145-2C11; Dilution 1:50), and Armenian hamster IgG isotype control PerCP/Cyanine5.5 (Cat. No. 400931; Lot. No. B247913; Clone HTK888; Dilution 1:50) were purchased from BioLegend (San Diego, California, USA). Anti-mouse CD3-FITC (Cat. No. 130-119-798; Lot. No. 5190919162; Clone REA641; Dilution 1:50), anti-mouse F4/80-FITC (Cat. No.130-117-509; Lot. No. 52003066886; Clone REA126; Dilution 1:50), REA Control-FITC (Cat. No. 130-113-449; Lot No. 5190711318; Clone REA293; Dilution 1:50), anti-mouse CD8b-APC (Cat. No. 130-111-712; Lot No. 5190919051; Clone: REA793; Dilution 1:50), Rat IgG2a isotype control-APC (Cat. No. 130102655; Lot. No. 5191030093; Dilution 1:10), anti-mouse CD4 VioBlue® (Cat. No. 130-118-696; Lot No. 5190919087; Clone REA605; Dilution 1:50), anti-mouse MHC Class II- VioBlue® (Cat. No. 130-123-278; Lot. No. 5200805636; Clone M5/114.15.2; Dilution 1:50), anti-mouse CD3ε VioBlue® (Cat. No. 130118849; Lot. No. 5200805619; Clone 17A2; Dilution 1:50), anti-mouse VioBlue® CD11b REAfinity™ (Cat. No. 130113810, Lot. No. 5200805605; Clone REA592; Dilution 1:50), REA Control-VioBlue® (Cat. No. 130-113-545; Lot. No. 5190711335; Clone REA293; Dilution 1:50), anti-mouse CD11b-PE-Vio®770 (Cat No. 130-113-808; Lot No. 5190919070; Clone REA592; Dilution 1:50), and REA Control-PE-Vio®770 (Cat. No. 130-113-452; Lot No. 5191025178; Clone REA293; Dilution 1:50) were purchased from Miltenyi Biotec (Bergisch Gladbach, Germany). **Western Blot antibodies:** Anti-human total p65 (Cat. No. D14E12; Lot. No. 13; Dilution 1:1000), anti-human phosphor-p65 (Cat. No.3033T; Lot. No. 16; Dilution 1:1000), anti-human vinculine (Cat. No. E1E9V; Lot. No. 6; Dilution 1:1000) and anti-rabbit HRP (Cat. No. 7074P2; Lot. No. 28; Dilution 1:2000) were purchased from Cells Signaling Technology® (Massachusetts, USA).

Brain-resident cell isolation. Brains from 6-10 week-old male C57BL/6 mice were harvested, chopped and incubated in rotation with Collagenase III\Dispase solution for 50 min at 37°C. Red blood-cells lysis was carried out followed by Percoll gradient for myelin separation. Cell suspension was then incubated with CD11b and CD31 microbeads for microglia and endothelial cells isolation, respectively, then placed with the microbeads in MACS MS magnetic columns. Isolated microglia were discarded while endothelial cells were plated in 96 well plates or Transwell® chambers following well-surface coating with 0.2 mg/ml rat tail type I collagen. The

negative population, enriched in astrocytes, was collected following beads separation, plated in a 10 cm^2 dish and grown in AM. After cells separation, each of the populations was evaluated for cell type and purity by FACS (Attune NxF, Life Technologies, USA).

mCherry FACS sorting. To assess the changes in astrocytes gene signature following interaction with melanoma cells, human or murine astrocytes were seeded ($1x10^6$ cells/10 cm^2 plate) and 24 h after mCherry-labeled 131/4-5B1, WM115, B16-F10, D4M.3A, B2905 cells, respectively, were added to the astrocytes culture ($0.5x10^6$ cells/10 cm^2 plate). Alternatively, astrocytes were grown alone in AM SFM for 24 h. Cells were detached and washed using PBS buffer supplemented with 2 mM EDTA and 0.5% BSA, and 0.1 % sodium azide (FACS buffer) and sorted by discarding mCherry-labeled melanoma cells by FACS (AriaIII, BD Bioscience, USA) using mCherry channel (635 nm). Total RNA was extracted from astrocytes for gene expression analysis.

Apoptosis assay in primary hippocampal cultures. Primary hippocampal neurons were prepared as described (*2*). The experiments were performed in cultures after 14 – 21 days *in vitro*. Cells were treated with 0.3 mM and 1 mM Bindarit o rvehicle (DMSO) for 1 h in MEM supplemented with 32.7mM glucose, or for 2 days in feeding medium. In order to detect apoptotic cells, coverslips were incubated with CellEvent™ Caspase-3/7 Green ReadyProbes™ Reagent according to manufactured protocol. Coverslips were imaged using FV1000 spectral Olympus confocal microscope using a 20x 0.75 N.A. objective under 488 nm (excitation) and 510 – 570 nm (emission) in an extracellular Tyrode solution containing 145 mM NaCl, 3 mM KCl, 15 mM glucose, 10 mM HEPES, 1,2 mM $MgCl_2$, and 1.2 mM $CaCl_2$, adjusted at pH 7.4 with NaOH. As positive control to induce cell death, primary hippocampal neurons were exposed for 30 minutes to 20 µM FCCP. For the analysis, ImageJ software was used to count the number of apoptotic cells relative to the neurons in the field of view.

siRNA MCP-1 silencing. Human astrocytes ($4x10^4$ cells/ml/well) were seeded onto 6-well plates and 24 h later transfected with polyplexes containing PEI and MCP-1 siRNA (NM_002982). siRNA-targeting MCP-1 (200 or 500 nM), or negative control siRNA (NC 500 nM) were incubated with

PEI for 20 min prior cells treatment, according to manufacturer's protocol. Twenty-four hours later cells were collected, and total RNA was extracted.

siRNA treatment and astrocytes viability. Human astrocytes ($4x10^4$ cells/ml/well) were seeded in 24-well plates and 24 h later transfected with polyplexes containing PEI:siRNA at 200 nM or 500 nM siRNA targeting MCP-1 or NC. Twenty-four to seventy-two hours later cells were detached using trypsin and counted using Coulter cells counter (Beckman, USA).

mRNA isolation and qPCR. Total RNA was isolated using EZ-RNA II total RNA isolation kit according to the manufacturer's protocol. Briefly, samples were lysed with 0.5 mL denaturing solution/6-well culture plate. Water saturated phenol was added, and samples were centrifuged. Isopropanol was then added to the aqueous colorless (upper) phase to precipitate RNA, and samples were centrifuged once more. RNA pellet was washed with 75% ethanol, centrifuged, and resuspended with ultra-pure double distilled water. RNA concentration was evaluated by measuring absorption (A260/A280) using NanoDrop® ND-1000 Spectrophotometer according to the manufacturer's V3.5 user's manual (Nano- Drop Technologies, Wilmington, DE). Reverse transcription reaction for mRNA was performed using qScript™ cDNA Synthesis Kit for RT-PCR, following the manufacturer's protocol. Briefly, 1 µg of total RNA sample was mixed with qScript Reverse Transcriptase, dNTPs and nuclease free water. The reaction tube was then incubated at 42°C for 30 min and heated at 85°C for 5 min to stop cDNA synthesis reaction. cDNA levels were quantified using custom qPCR primers and normalized to GAPDH or HPRT housekeeping genes. qPCR for cDNAs was performed using Fast SYBR™ green Master Mix, according to manufacturer's instructions using StepOnePlus real-time PCR system (Applied Biosystems, Thermo Fisher, USA). **Human primers:** Human MCP-1 Forward: 5'- GGCTGAGACTAACCCAGAAAC – 3'; reverse: 5' - GAATGAAGGTGGCTGCTATGA - 3'. **Murine primers:** Mouse MCP-1 Forward: 5'- TGTGTTCATCCCCAGAACCG -3'; reverse: 5'GGGTACAGTTCCTTGGAGCC -3'. Mouse CCR2 Forward: 5'- TGTGTTCATCCCCAGAACCG-3': reverse: 5'- GGGTACAGTTCCTTGGAGCC -3'. Mouse HPRT Forward: 5' - TGATTATGGACAGGACTGAAAGA -3'; reverse: 5' – GCAGGTCAGCAAAGAACTTATAG - 3'.

Human/murine primers: human/mouse GAPDH Forward: 5' - ATTCCACCCATGGCAAATTC - 3'; reverse: 5' - GGATCTCGCTCCTGGAAGATG - 3'.

Melanoma proliferation in co-culture. Human and murine melanoma mCherry-labeled cells (WM115 – $2x10^3$ cells/well, A375 – $1x10^3$cells/well, D4M.3A - 750 cells/well, B16-F10 – 1 $x10^3$ cells/well) were plated alone onto 96-well culture plates in their growing medium for 24 h. In addition, melanoma cells were co-cultured with human or murine astrocytes at a ratio of 1:1 in AM. Twenty-four hours later, astrocytes SFM was added to the cell mono-culture or the co-culture. Cells' growth was monitored for 48 h by IncuCyte^{TM} ZOOM Live Cell Imaging system (Essen BioScience, Sartorius, USA) and red phase images (representing mCherry-labeled melanoma cells) were taken every two hours using 10X objective. Results were calculated by the IncuCyte^{TM} Software and presented as % of total red area covered (image/well) normalized to time zero for each cell cultures. Additionally, human WM115 melanoma mCherry cells ($2x10^3$ cells/well) were co-cultured with astrocytes (1:1), or microglia (2:1), or CMEC/D3 (1:2) in their growing medium. Twenty-four hours later, brain resident cells SFM supplemented with 10µg/ml anti-MCP-1 neutralizing antibody, was added to the co-cultures. Cell growth was monitored for 48 h by IncuCyte^{TM} ZOOM Live Cell Imaging system (Essen BioScience, Sartorius, USA) and red phase images (representing mCherry-labeled melanoma cells) were taken every two hours using 10X objective. Results were calculated by the IncuCyte^{TM} Software and presented as % of total red area covered (image/well) normalized to time zero for each cell cultures.Fhehem

Transwell® Migration Assay. For testing melanoma migration towards astrocytes-secreted cytokines enriched medium, melanoma 131/4-5B1 or D4M.3A ($1x10^5$ cells/insert), WM115 or B16-F10 cells ($5x10^4$ cells/insert) were seeded in 8 µm inserts of a Transwell® coated with 10 µg/mL bovine fibronectin. Astrocytes SFM or CM was loaded in the lower chamber and migration of the melanoma cells towards SFM or CM was monitored for 12 to 48 h. In order to assess melanoma migration in co-culture with astrocytes, human astrocytes ($1x10^5$ cells/well) or murine astrocytes ($2x10^5$ cells/well) were seeded in the lower chambers of the Transwell® inserts. Astrocytes SFM alone or supplemented with 0.3 mM bindarit was added to growing astrocytes

in the lower chamber. Melanoma cells (131/4-5B1, A375, WM115, D4M.3A, or B16-F10) were then allowed to migrate towards untreated or treated astrocytes for 12 to 48 h.

D4M.3A cells were allowed to migrate towards astrocytes' CM collected from treated astrocytes (0.3 mM bindarit) or towards astrocytes CM supplemented with freshly added 0.3 mM bindarit, as additional control to exclude cytotoxic effects of the bindarit on the migrating cells.

In order to evaluate melanoma migration towards astrocytes CM depleted of specific cytokines, murine (B16-F10 $5x10^4$ cells/well or D4M.3A - $1x10^5$ cells/well) and human (WM115 or 131/4-5B1 – $5x10^4$ cells/well) melanoma cells were seeded in the upper chambers of the Transwell®. Astrocytes SFM, astrocytes CM alone or supplemented with neutralizing antibodies (RANTES, MCP-1, GRO-α, or SERPIN-E1), were loaded in the lower chamber. Melanoma cells were then allowed to migrate towards the astrocytes CM supplemented with neutralizing antibodies for the selected cytokines for 12 to 24 h. For the D4M.3A and B16-F10 migration assays, astrocytes were previously activated with LPS for 24 h prior CM collection, as described before. Anti-human neutralizing antibodies for RANTES (0.1 µg/ml), MCP-1 (0.2 µg/ml), GRO-α (0.1 µg/ml), SERPIN E1 (0.1 µg/ml), IL-6 (0.4 µg/ml), IL-8 (0.2 µg/ml) were used according to the manufacturer's protocol. Anti-mouse neutralizing antibodies for RANTES (0.6 µg/ml), MCP-1 (12 µg/ml), GRO-α (0.5 µg/ml), SERPIN E1 (4 µg/ml), IL-6 (0.016 µg/ml), MIP-2 (1.8 µg/ml), were used according to the manufacturer's protocol.

In order to evaluate the effect on melanoma migration of other brain resident cells which secrete MCP-1, human WM115 melanoma cells ($1x10^5$ cells/well) were seeded in the upper chambers of the Transwell®. Melanoma cells were let to migrate towards human astrocytes, or human microglia, or CMEC/D3 for 12h in SFM (AM, or MM, or EndoGRO media). Control co-culture were compared to treated co-culture, where in the latter MCP-1 was neutralized by treating the brain resident cells with 10 µg/ml anti-MCP-1 neutralizing antibody.

In order to rescue melanoma cells migration following neutralization of MCP-1 in co-culture with astrocytes or in astrocytes CM, human WM115 melanoma cells ($1x10^5$ cells/well) were seeded in the upper chambers of the Transwell®. Melanoma cells were let to migrate towards untreated astrocytes, or bindarit-treated astrocytes, or bindarit-treated astrocytes supplemented with 1 µg/ml rh MCP-1. Alternatively, melanoma cells were let to migrate towards the CM of untreated

astrocytes, or bindarit-treated astrocytes, or bindarit-treated astrocytes supplemented with 1 µg/ml rhMCP-1 for 18h.

In order to evaluate the toxicity on brain resident cell migration of bindarit and anti-MCP-1 neutralizing antibody, human astrocytes, or human microglia, or CMEC/D3 ($1x10^5$ cells/well) were seeded in the upper chambers of the Transwell®. The brain resident cells were exposed to 0.3 mM bindarit, or 10 µg/ml anti-MCP-1 neutralizing antibody and let to migrate towards serum-containing media (AM, or MM, or EndoGRO media) for 24 h. All migrated cells were then fixed and stained (Hema 3 Stain System; Fisher Diagnostics, fixed and stained (Hema 3 Stain System; Fisher Diagnostics, USA). The stained migrated cells were imaged using EVOS FL Auto microscope (Life Technologies, USA) using 10X objective, brightfield illumination.

For transendothelial migration, freshly isolated mBEC cells ($4x10^5$ cells/insert) were seeded and grown for 96 h in 8 µm Transwell® inserts coated with 0.2 mg/ml collagen type I. mCherry-labeled D4M.3A or B16-F10 cells were seeded on 100 ng/ml collagen type I added on top of mBEC. Melanoma cells were allowed to migrate for 48 h towards treated astrocytes (0.3 mM bindarit) or 0.3 mM bindarit pre-treated astrocytes CM. Migrated cells were then fixed, and the nuclei were stained with Hoechst solution in PBS (1:5000). Migrated mCherry-labeled cells were imaged using EVOS FL Auto microscope (Life Technologies, USA) using 10X objective, Tex-Red LED Cube illumination. Migrated cells from the captured images per membrane were analyzed using ImageJ 1.52v software.

Wound Healing Assay. To study the role of MCP-1 in mediating melanoma cells migration, (WM115 and 131/4-5B1 cells) ($5x10^4$ cells/well) were plated onto 96-well ImageLock tissue culture plate and allowed to grow until confluence. Next, a wound was created in each well using a 96-pin wound-making tool (WoundMaker, Essen BioScience, Sartorius, USA), and dislodged cells were washed with PBS. Melanoma cells were treated for 48h with astrocytes CM, human astrocytes CM treated with siRNA:PEI polyplexes (500 nM siRNA targeting MCP-1 or scramble siRNA), astrocytes CM supplemented with 10 µg/mL anti-MCP-1 neutralizing antibody, astrocytes SFM supplemented with 1 µg/mL rh/m-MCP-1. To monitor wound closure, the plate was placed in the IncuCyte™ ZOOM Live Cell Imaging system (Essen Bioscience, Sartorius, USA) and phase

contrast images were taken every 2 h intervals over a course of 48 h using 10X objective. Results were calculated by the IncuCyte^{TM} Software and presented as relative wound density (relative to the background density of the wound at time 0).

ELISA. Activation of human or murine astrocytes was achieved by growing 8x10^4 cells/6 wells-plate in astrocytes SFM (with LPS in case of murine cells) for 24 h. Inhibition of human or murine astrocytes-secreted MCP-1 was achieved by growing 8x10^4 cells/6 wells-plate in astrocytes SFM supplemented with 0.3 mM bindarit (with LPS in case of murine cells). Alternatively, human astrocytes (4x10^4 cells/6 wells-plate) were grown in astrocytes medium supplemented with 2% serum for 24 h. PEI complexed with MCP-1 siRNA and NC siRNA were added to the medium. Twenty-four hours following treatment, the cells were incubated in AM SFM for additional 24 h. To assess MCP-1 secretion in melanoma-activate astrocytes, human melanoma WM115 cells or murine melanoma D4M.3A (5x10^5 cells/10 cm^2 dish) were grown in melanoma SFM. Twenty-four hours later, melanoma CM was collected, filtered with 0.2 µm filter and added to growing human and murine astrocytes (2.5x10^5 or 5x10^{5}cells/10 cm^2 dish), respectively. MCP-1 inhibition in melanoma activated-astrocytes was achieved by exposing astrocytes to WM115 CM supplemented with 0.3 mM bindarit. CM of melanoma cells was used to determine the basal MCP-1 melanoma containing-medium prior to astrocytes enrichment secretion. Twenty-four hours later astrocytes CM or melanoma CM was collected, filtered with 0.2 µm filter and analyzed for MCP-1 secretion. In order to evaluate MCP-1 secretion from brain resident cells, astrocytes (1x10^5 cells/6 well-plate), or microglia (0.5x10^5 cells/6 well-plate), or CMEC/D3 (2x10^5 cells/6 well-plate) were either grown in monoculture or in co-culture with WM115 (1x10^5 cells/6 well-plate) melanoma cells for 24 h in SFM. Twenty-four hours later astrocytes, or microglia, or CMEC/D3 CM was collected, filtered with 0.2 µm filter and analyzed for MCP-1 secretion, while the cells were harvested and analyzed by flow cytometry for intracellular MCP-1 expression.

Multicellular tumor spheroids (MCTS). 3D tumor spheroids were formed from a mixture of melanoma and astrocytes. Human or murine mCherry-labeled melanoma cells (WM115, A375, D4M.3A, B16-F10, B2905 – 4x10^5 cells/ml) alone or in combination with human GFP-labeled or

unlabeled murine astrocytes ($4x10^5$ cells/ml - in ratio 1:1) were grown in AM supplemented with 0.24 w/v% methyl cellulose. Cells were seeded in 25 µL droplets on the inner side of a 20 mm dish and incubated for 48 h at 37°C when the plate is facing upside down to allow for spheroid formation. For basal invasion assay, 3D spheroids were then embedded in GFR Matrigel® and exposed to astrocytes SFM. For cytokines-induced invasion, 3D mono-culture spheroids were grown in astrocytes CM. In order to evaluate the inhibition of invasion following bindarit treatment, WM115 and D4M.3A ($4x10^5$ cells/ml) alone or in combination with human or murine astrocytes ($4x10^5$cells/ml - in ratio 1:1) were exposed to astrocytes SFM alone or supplemented with 0.3 mM bindarit, or to untreated astrocytes CM or supplemented with 0.3 mM bindarit-treated astrocytes. 3D MCTS invasion was visualized following 24 h and 48 h using EVOS FL Auto cell imaging system (Life Technologies, USA) at 10X magnification. The sprouting of MCTS was analyzed using ImageJ 1.52v software, and the results were presented as total area covered or % of total area covered per spheroid (pixel density) per well.

Hemolysis Assay. Hemolysis Assay. Fresh blood was obtained from a male Wistar rat (~250 g) by cardiac puncture and collected in a heparinized. Blood was then centrifuged at 1000 x g for 10 min at 4°C. Supernatant was discarded and erythrocytes were washed 3 times with pre-chilled PBS. Then a 2% w/w RBC stock solution (2% RBC in cold PBS) was loaded into a 96-well plate and incubated with serial dilutions (0.31-2.5 mg/ml) of bindarit for 1 h at 37°C. Following plate centrifugation, the supernatants were transferred to a new plate and absorbance was measured at 550 nm using a SpectraMax M5 plate reader (Molecular Devices, San Jose, California, USA). SDS and Triton-X100 were used as positive controls, whereas 70kDa Dextran was used as a negative control.

Evans blue permeability Miles assay. B16-F10 MBM bearing mice were injected i.v. with 0.15 ml of Evans blue solution (30 mg/ml in saline) (n=3 PBS treated-group; n=4 bindarit-treated group). Thirty minutes later, mice were perfused with PBS, brains were harvested, and intracranially tumors were isolated and incubated in 0.5 ml formamide (Sigma-Aldrich, Israel) for 48 h at 55°C.

The absorbance of the Evans blue dye was then measured at 620 nm and the blank (formamide only) was subtracted to the Evans blue measurements, using SpectraMax M5 plate reader (Molecular Devices, San Jose, California, USA).

CCR2 and CCR4 markers sorting. For basal expression of CCR2 and CCR4 receptors, B16-F10 cells were grown till confluent and harvested using FACS buffer. CCR2-APC and CCR4-PE primary antibodies were incubated in FACS buffer with the cell's suspension for 30 min at room temperature. CCR2+/CCR4+ B16-F10 expressing cells were sorted using AriaIII (BD Bioscience, USA) and cultured for a week melanoma medium. The sorted cells underwent CRISPR/Cas9 K/O or NTC K/O and then sorted again for CCR2-/CCR4- following incubation with CCR2-PE and CCR4-APC.

CRISPR/Cas9 for CCR2/CCR4 melanoma B16-F10 knockout (K/O). For B16-F10 CCR2/CCR4 knock-out (K/O), cells were sorted for $CCR2^+/CCR4^+$ expression and cultured in plate for a week. Then, electroporation was performed on 1 x 10^5cells/µl with 18.3 pmol Alt-R spCas9 Nuclease V3, and 22 pmol sgRNA, in Buffer R using a Neon electroporation system at 1600v 20ms 1pulse. CRISPR/Cas9 negative K/O was generated in absence of gRNAs (NTC). Subsequently, cells were grown overnight in a 6-well plate at the concentration of 1 x10^6 cells/ml in antibiotic-free media. K/O assessment was then performed by flow cytometry using Attune NxT on day 3 following electroporation. Finally, B16-F10 cells were sorted for CCR2-/CCR4-.
CRISPR/Cas9 sgRNA sequences: CCR2 5'- AGTATGCCGTGGATGAACTG -3'; CCR4 5'– CAGACCCAACAAGAAGACCA -3'.

CCR2 and CCR4 K/O genes quantification. For quantification of gRNA activity, genomic DNA was extracted using Quick DNA miniprep kit and 500ng genomic DNA was amplified by PCR using PrimeSTAR MAX for 35cyles. Resulting amplicon was denatured and reannealed in a thermocycler prior to cleaving by T7 Endonuclease 1 at 37°C for 30 min. DNA cleavage was analyzed by agarose gel electrophoresis and quantified using Biovision (Vilber Lourmat) using a rolling ball for background subtraction. Efficiency of gene editing was calculated as cleavage efficiency (*3*).

Cleavage efficiency: 100 x (1 – (1 – fraction cleaved)$^{1/2}$.

Fluorescence microscopy and image analysis. Confocal images were captured using a Nikon Ti microscope equipped with a Yokogawa CSU X-1 spinning disc, X60 oil objective and an Andor iXon897 EMCCD camera, controlled by Andor IQ3 software. 3-dimensional co-localization analysis was done using Bitplane Imaris 8.4.3 software.

Generation of MBM. In order to generate MBM, melanoma cells were either intracardially-innoculated (spontaneous MBM tumor) or intracranially-inoculated (melanoma brain tumor) into immunocompromised SCID mice, or immunocompetent C57BL/6 mice. The cell lines used in this study, with the exception for D4M.3A, WM115 and B2905 melanoma cells, were previously reported to form spontaneous brain metastasis following either intracardiac injection of melanoma cells, or resection of the primary tumors. The location of those metastases are predominantly parenchymal and leptomeningeal in the murine cell lines (Mel-ret, B16-F10 (*4-6*), D4M.3A and B2905 in Table 1), as opposed to the human cell lines (131/4-5B1, A375 in Table 1) which prefentially colonize the brain parenchyma (*7, 8*). The yield of brain metastasis following intracardiac inoculation of non-brain tropic melanoma cells, varies between 7 to 60% (*5, 9-11*) and between 20 to 54% following primary tumor resection (97–180 days post-primary resection) (*7, 12*). In our spontaneous models of MBM (intracardiac inoculation of melanoma cells) the incidence of MBM was 100% in 131/4-5B1 model (n=10), 0% in WM115 model (n=10), 90% in B2905, and 70% in D4M.3A model (n=9) in 3-6 weeks. WM115 model developed MBM (66%, n=6) following 2-3 months from intracardiac cell inoculation. However, with the exception of 131/4-5B1 model, in most of the cases mice succumbed from lungs and intraperitoneal lesions while showing signs of brain metastasis (Table 2). The incidence raised to 60% and 80-90% following primary resection (in 4–6 months) or intracardiac inoculation of brain-tropic melanoma cells (*9, 12*). For interventional purpose, MBM were generated following intracranial inoculation of melanoma cells. Examples in literature exploiting the model of intracranial inoculation of D4M.3A and B16-F10 were used for interventional immunotherapy studies (*13, 14*).

Magnetic Resonance Imaging (MRI). Tumor bearing mice were imaged at the Sackler Cellular &

Molecular Imaging Center (SCMIC), Tel Aviv University. Tumor bearing mice were scanned using 4.7T/1H MRS 4000™ or 7T MRI (MR Solutions, UK) (32-channel head-coil) following gadolinium i.p. injection. MRI scans were taken using a conventional T1 according to FSE26 sequence. T1 data were analyzed using RadiAnt DICOM Viewer or MRIcro software, and the tumor area was calculated per each axial scan. The tumor volume was obtained by summing the tumor areas from each scan.

References

1. E. Segal *et al.*, Targeting angiogenesis-dependent calcified neoplasms using combined polymer therapeutics. *PLoS One* **4**, e5233-e5233 (2009).
2. B. Styr *et al.*, Mitochondrial Regulation of the Hippocampal Firing Rate Set Point and Seizure Susceptibility. *Neuron* **102**, 1009-1024.e1008 (2019).
3. D. Y. Guschin *et al.*, A rapid and general assay for monitoring endogenous gene modification. *Methods Mol Biol* **649**, 247-256 (2010).
4. L. Balathasan, J. S. Beech, R. J. Muschel, Ultrasonography-Guided Intracardiac Injection: An Improvement for Quantitative Brain Colonization Assays. *The American Journal of Pathology* **183**, 26-34 (2013).
5. H. Schwartz *et al.*, Incipient Melanoma Brain Metastases Instigate Astrogliosis and Neuroinflammation. *Cancer Research* **76**, 4359-4371 (2016).
6. G. Schackert, I. J. Fidler, Site-specific metastasis of mouse melanomas and a fibrosarcoma in the brain or meninges of syngeneic animals. *Cancer Res* **48**, 3478-3484 (1988).
7. W. Cruz-Munoz, S. Man, P. Xu, R. S. Kerbel, Development of a Preclinical Model of Spontaneous Human Melanoma Central Nervous System Metastasis. *Cancer Research* **68**, 4500-4505 (2008).
8. N. Herwig, B. Belter, J. Pietzsch, Extracellular S100A4 affects endothelial cell integrity and stimulates transmigration of A375 melanoma cells. *Biochemical and Biophysical Research Communications* **477**, 963-969 (2016).
9. H. Doron *et al.*, Inflammatory Activation of Astrocytes Facilitates Melanoma Brain Tropism via the CXCL10-CXCR3 Signaling Axis. *Cell Reports* **28**, 1785-1798.e1786 (2019).
10. M. Amit *et al.*, Characterization of the melanoma brain metastatic niche in mice and humans. *Cancer Med* **2**, 155-163 (2013).
11. M. Nakai *et al.*, Establishment of a murine model for metastasis of cytokine-producing tumor to the brain. *Pigment Cell Res* **10**, 304-309 (1997).
12. M. Valiente *et al.*, Brain Metastasis Cell Lines Panel: A Public Resource of Organotropic Cell Lines. *Cancer Research* **80**, 4314-4323 (2020).
13. L. W. Pfannenstiel *et al.*, Combination PD-1 blockade and irradiation of brain metastasis induces an effective abscopal effect in melanoma. *OncoImmunology* **8**, e1507669 (2019).
14. N. Nayyar, M. Singh, J. Stocking, M. Brehm, P. Brastianos, 62. PRESENCE OF EXTRACRANIAL TUMORS INFLUENCES RESPONSE TO IMMUNE CHECKPOINT INHIBITORS IN A PRE-CLINICAL MODEL OF MELANOMA BRAIN METASTASIS. *Neurooncol Adv* **2**, ii13-ii13 (2020).

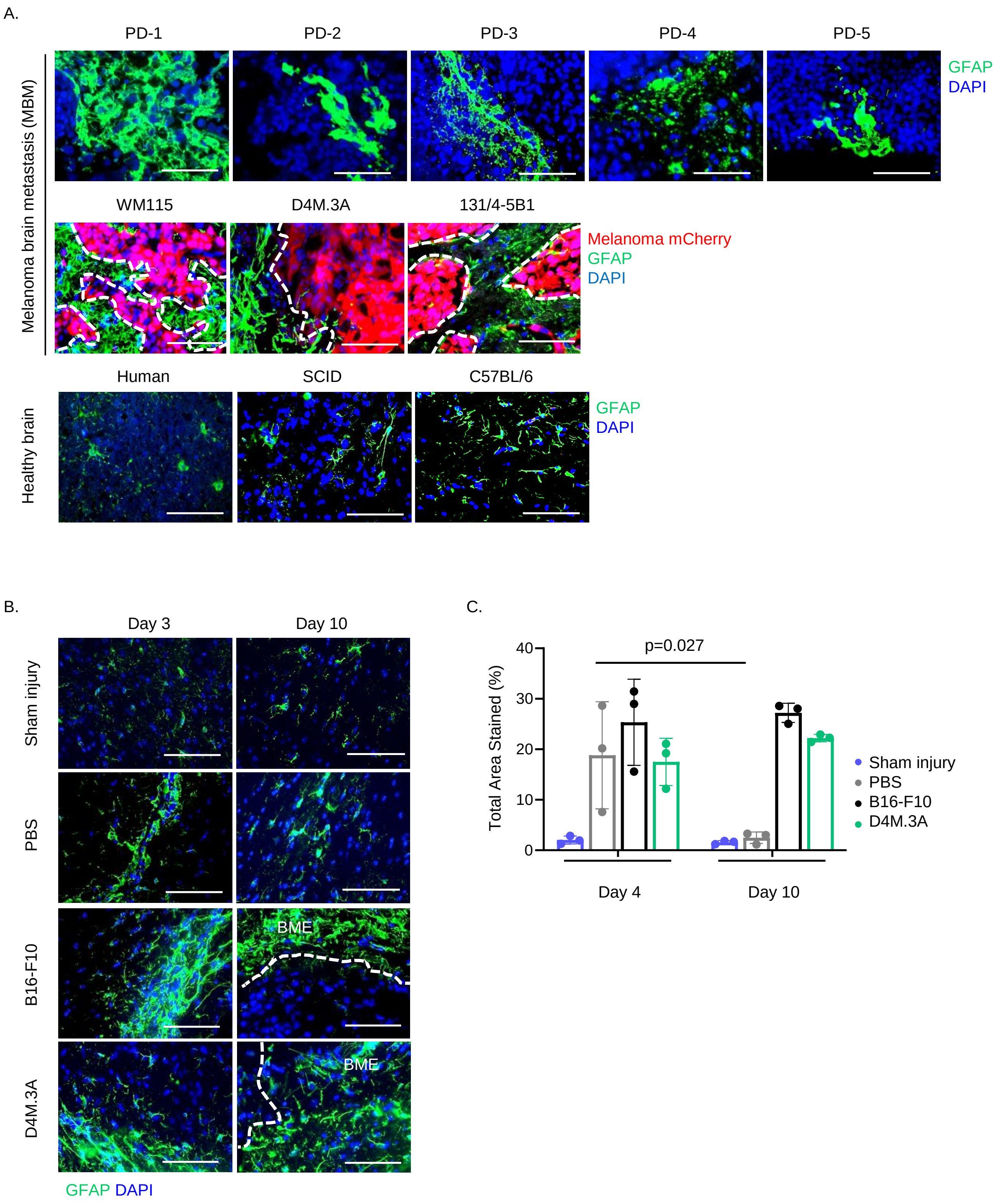
A.
PD-1
PD-2
PD-3
PD-4
PD-5
GFAP
DAPI
Melanoma brain metastasis (MBM)
WM115
D4M.3A
131/4-5B1
Melanoma mCherry
GFAP
DAPI
Healthy brain
Human
SCID
C57BL/6
GFAP
DAPI
B.
Day 3
Day 10
Sham injury
PBS
B16-F10
D4M.3A
BME
BME
GFAP DAPI
C.
Total Area Stained (%)
40
30
20
10
0
p=0.027
Day 4
Day 10
Sham injury
PBS
B16-F10
D4M.3A

Supplemental Figure 1. Activated-astrocytes in MBM (A) GFAP immunostaining (green) showing activated astrocytes in melanoma tumors compared to normal brain samples of human and mouse (C57BL/6 and SCID, n=3 mice). Representative images of FFPE of patient-derived (PD-1, PD-2, PD-2, PD-4, and PD-5) N=5 patients; cryo-section of melanoma brain metastases (MBM) of human WM115 (n=4 mice) and murine D4M.3A (n=3 mice) mCherry-labeled cells following intracardiac cell inoculation (red). Nuclei are shown by DAPI staining (blue). Scale bar - 100 µm. **(B)** Astrocytes morphology and GFAP activation of brain cryo-section with sham injury and PBS intracranially-injected compared to B16-F10 and D4M.3A tumor-bearing mice (n=3 mice). Nuclei are shown by DAPI staining (blue), GFAP (green). Scale bar - 100 µm. **(C)** Quantification of GFAP marker staining following sham injury (in blue), PBS administration (in gray), B16-F10 (in black) or D4M.3A (in green) melanoma cells intracranially-inoculated. Pixel density (total area covered in % per field) of GFAP marker. Dots represent mean ± S.D. of n= 3 fields/slide in n=3 mice of biological replicates. Ordinary Two-way ANOVA statistical analysis.

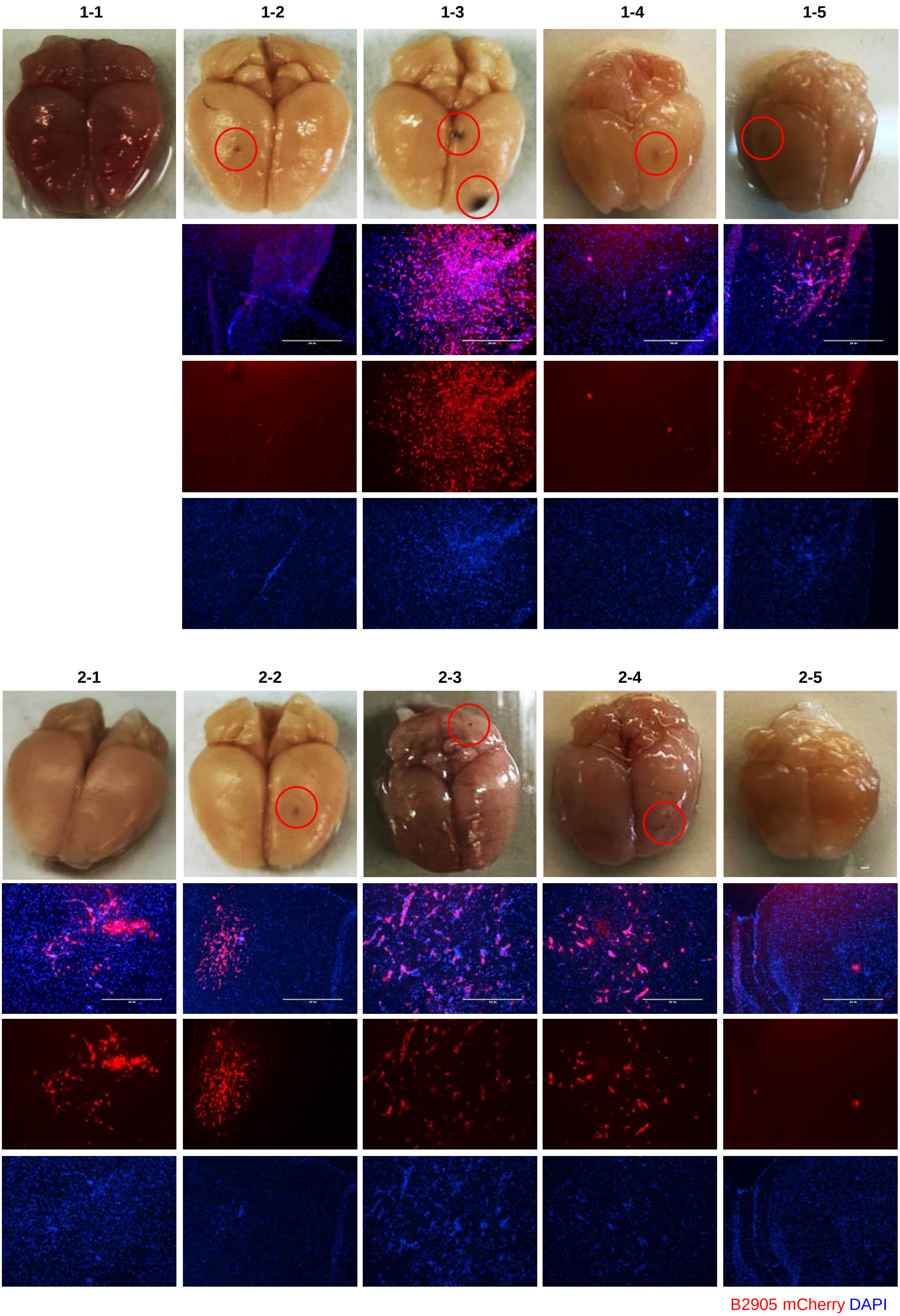
1-1
1-2
1-3
1-4
1-5
2-1
2-2
2-3
2-4
2-5
B2905 mCherry DAPI

Supplemental Figure 2. Spontaneous formation of B2905 MBM following intracardiac cell inoculation. B2905 melanoma cells were intracardially inoculated in immunocompetent mouse model (n=10) to generate MBM. Upper panel – photos of the harvested brains in which visually detectable MBM are marked in red (red cycle). Lower panel - IHC for mCherry marker showed the presence of MBM detected in the parenchyma or in leptomeningeal location.. Brain specimens that did not develop MBM, did not show any positive staining for mCherry and therefore were left blank. Nuclei are shown by DAPI staining (blue), mCherry (red). Scale bar - 400 µm.

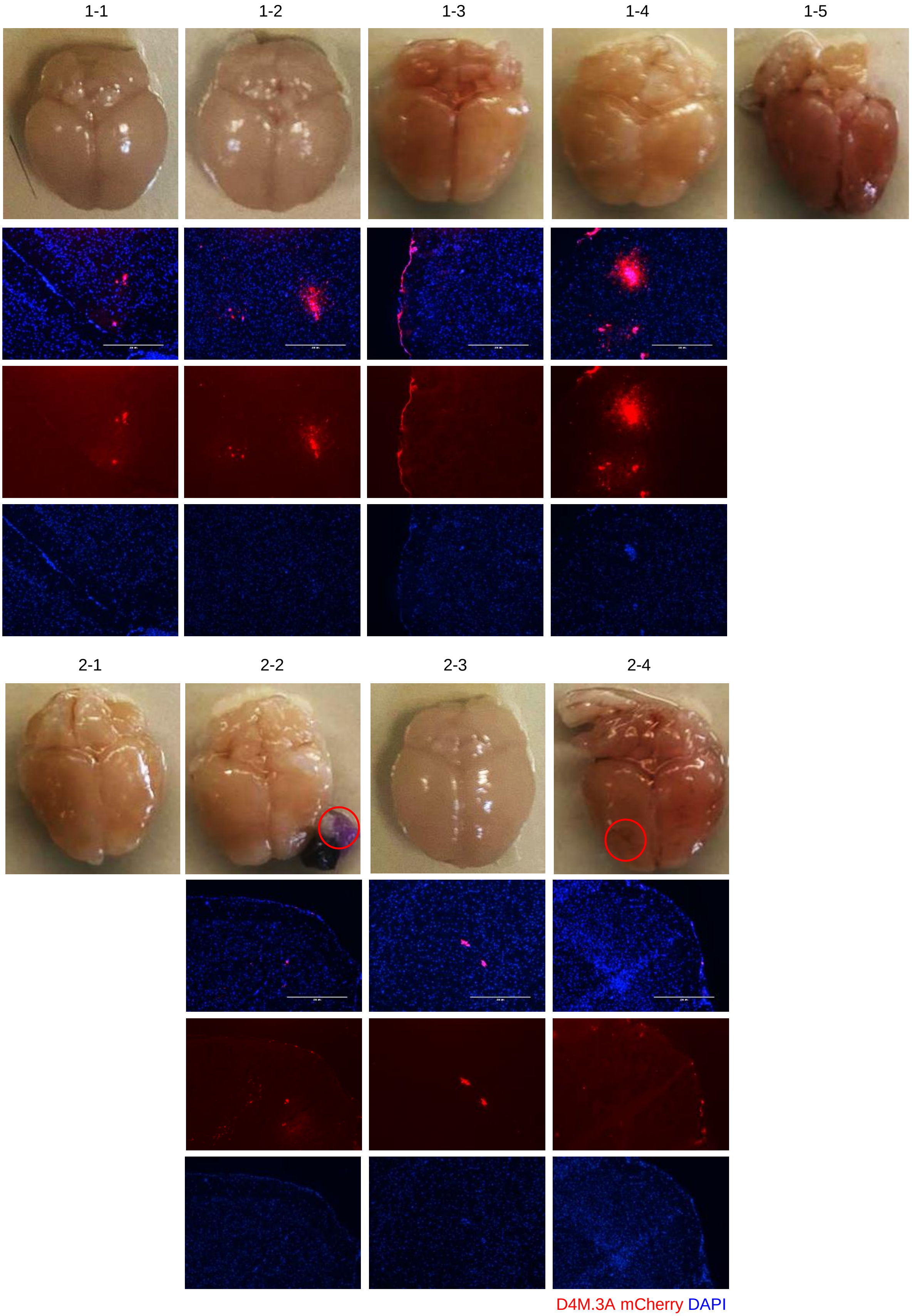
1-1
1-2
1-3
1-4
1-5
2-1
2-2
2-3
2-4
D4M.3A mCherry DAPI

Supplemental Figure 3. Spontaneous formation of D4M.3A MBM following intracardiac cell inoculation. D4M.3A melanoma cells were intracardially inoculated in immunocompetent mouse model (n=9) to generate MBM. Upper panel – photos of the harvested brains in which visually detectable MBM are marked in red (red cycle). Lower panel - IHC for mCherry marker showed the presence of MBM detected in the parenchyma or in leptomeningeal location. Brain specimens that did not develop MBM, did not show any positive staining for mCherry and therefore were left blank. Nuclei are shown by DAPI staining (blue), mCherry (red). Scale bar - 400 µm.

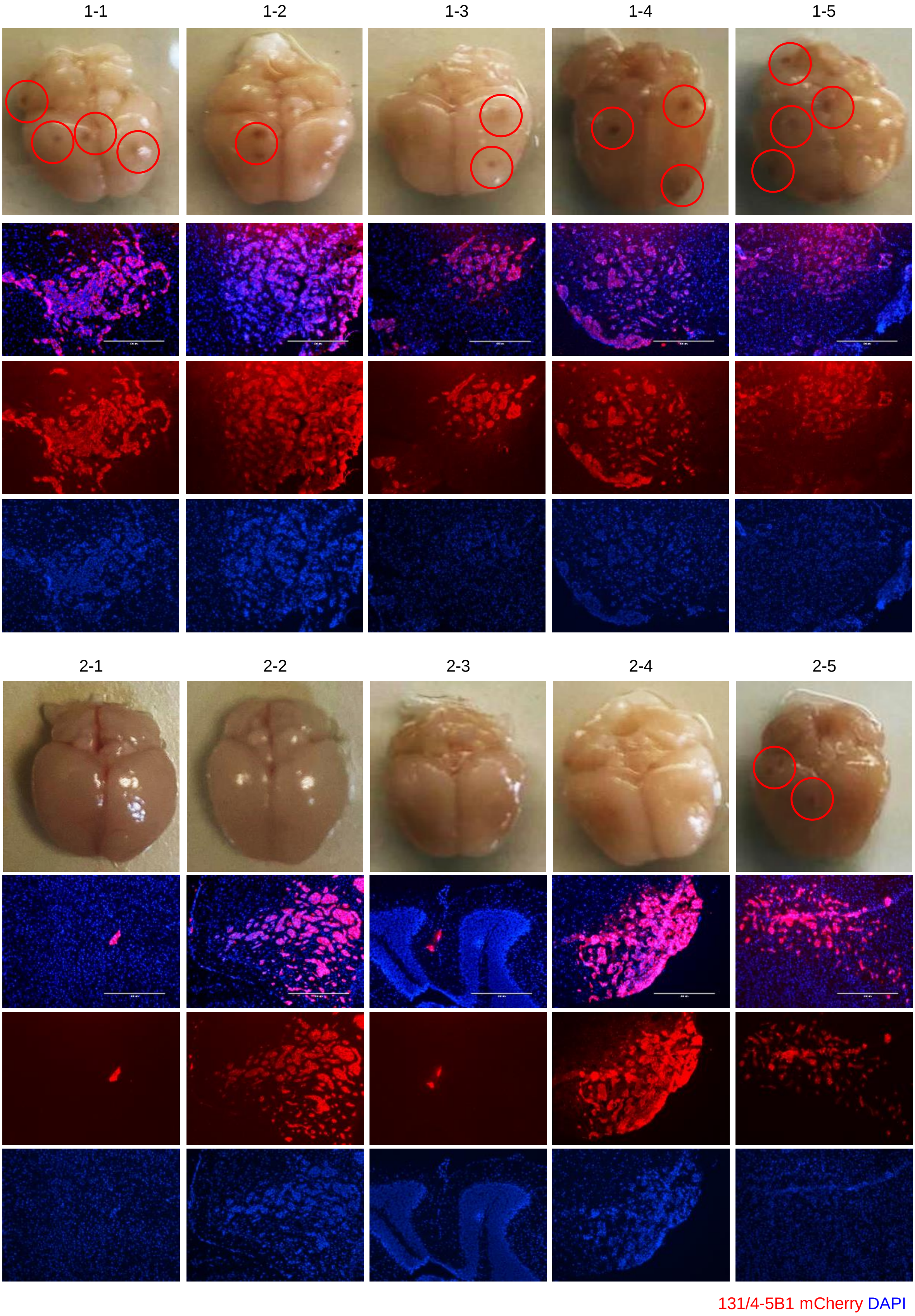
1-1
1-2
1-3
1-4
1-5
2-1
2-2
2-3
2-4
2-5
131/4-5B1 mCherry DAPI

Supplemental Figure 4. Spontaneous formation of 131/4-5B1 MBM following intracardiac cell inoculation. 131/4-5B1 melanoma cells were intracardially inoculated in immunocompromised mouse model (n=10) to generate MBM. Upper panel – photos of the harvested brains in which visually detectable MBM are marked in red (red cycle). Lower panel - IHC for mCherry marker showed the presence of MBM detected in the parenchyma or in leptomeningeal location. Nuclei are shown by DAPI staining (blue), mCherry (red). Scale bar - 400 μm.

A.

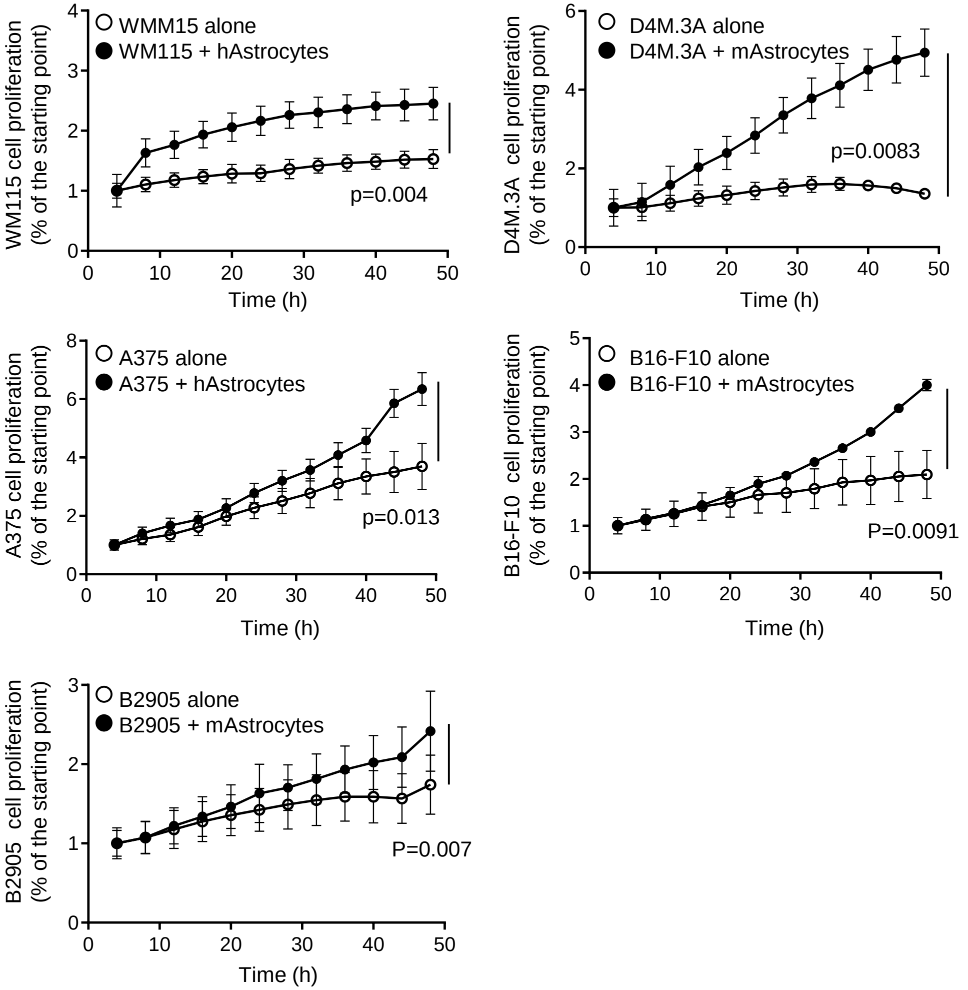
WMM15 alone
WM115 + hAstrocytes
WM115 cell proliferation (% of the starting point)
p=0.004
Time (h)
D4M.3A alone
D4M.3A + mAstrocytes
D4M.3A cell proliferation (% of the starting point)
p=0.0083
Time (h)
A375 alone
A375 + hAstrocytes
A375 cell proliferation (% of the starting point)
p=0.013
Time (h)
B16-F10 alone
B16-F10 + mAstrocytes
B16-F10 cell proliferation (% of the starting point)
P=0.0091
Time (h)
B2905 alone
B2905 + mAstrocytes
B2905 cell proliferation (% of the starting point)
P=0.007
Time (h)

B.

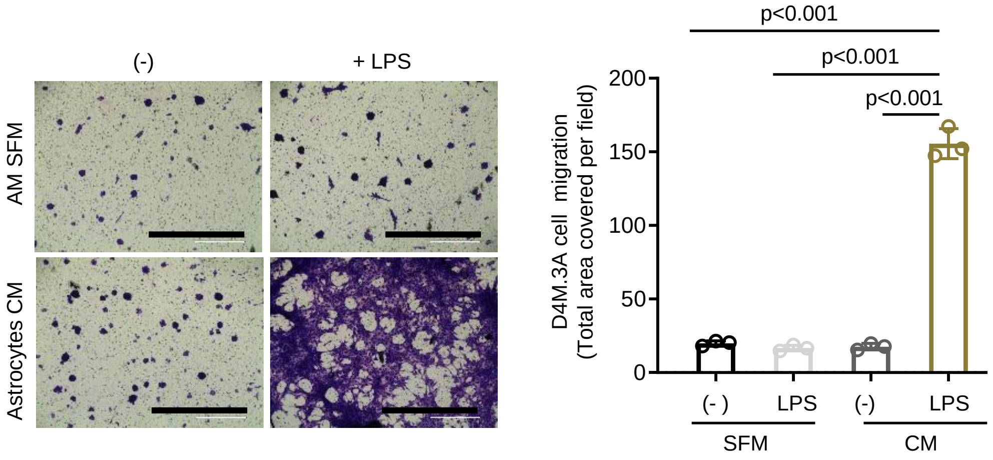
(-)
+ LPS
AM SFM
Astrocytes CM
p<0.001
p<0.001
p<0.001
D4M.3A cell migration (Total area covered per field)
(-)
LPS
(-)
LPS
SFM
CM

Supplemental Figure 5. Astrocyte activation promotes melanoma growth and migration. (A) Co-culture with astrocytes enhances melanoma proliferation (WM115, A375, D4M,3A, B16-F10, and B2905 melanoma cells) in 48 h. Data is presented as the mean ± S.D. fluorescence signal of mCherry-labeled melanoma, measured by Incucyte™ live cell imaging. Dots represent mean ± S.D. of n= 4 fields per well of three wells of biological replicates. Two-way ANOVA statistical analysis was performed. **(B)** Migration of D4M.3A melanoma cells in Transwell® in the presence of astrocytes (AS) SFM (in black), AS SFM + LPS (100 ng/ml – in light gray), AS CM (in dark gray), or AS-LPS activated CM (in taupe) for 48 h. Pixel density (total area covered per field) of the migrated cancer cells was quantified using ImageJ 1.52v software. Representative cell migrating fields are shown (n=3). Scale bar - 400 µm. Dots represent mean ± S.D. of n= 3 fields/well of triplicates of biological replicates. Ordinary one-way ANOVA statistical analysis.

A.

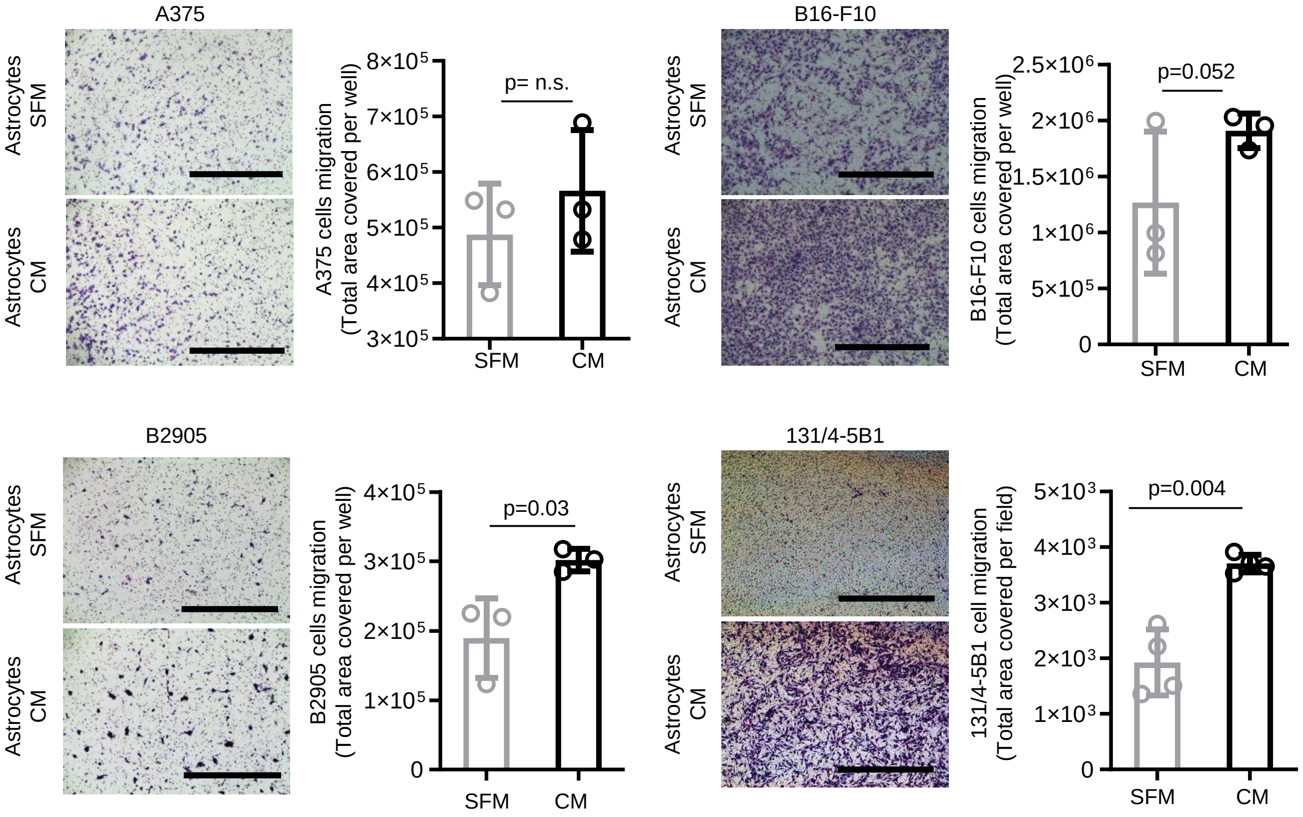

A375
Astrocytes SFM
Astrocytes CM
A375 cells migration (Total area covered per well)
p= n.s.
SFM
CM
B16-F10
B16-F10 cells migration (Total area covered per well)
p=0.052
B2905
B2905 cells migration (Total area covered per well)
p=0.03
131/4-5B1
131/4-5B1 cell migration (Total area covered per field)
p=0.004

B.

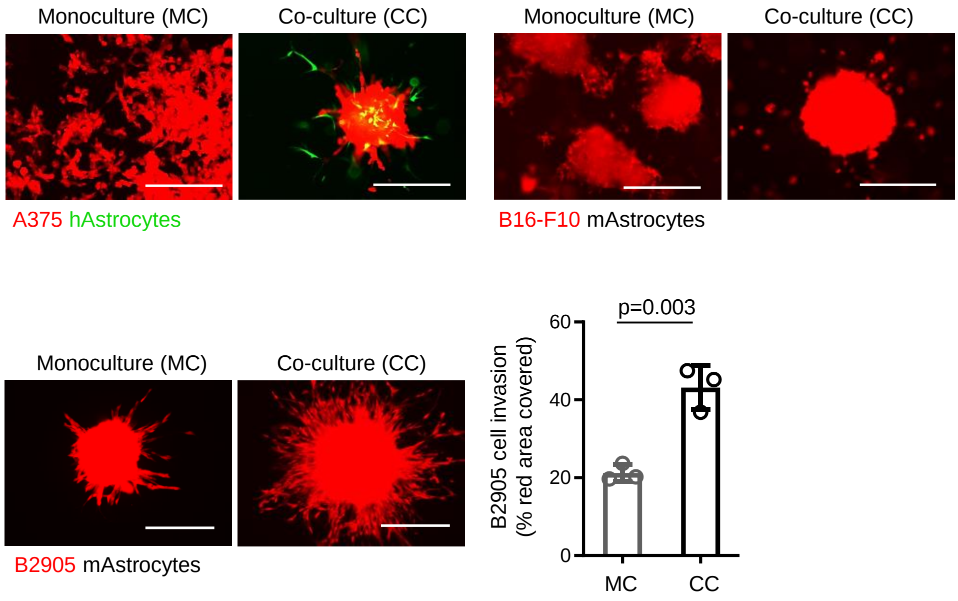

Monoculture (MC)
Co-culture (CC)
A375 hAstrocytes
B16-F10 mAstrocytes
B2905 mAstrocytes
B2905 cell invasion (% red area covered)
p=0.003
MC
CC

Supplemental Figure 6. Astrocyte activation promotes melanoma migration and invasion. (A) Astrocytes conditioned-media (CM - black) enhance melanoma Transwell® migration (A375, B16-F10, B2905 and 131/4-5B1 cells). Pixel density (total area covered per field) of the migrated cancer cells was quantified using ImageJ 1.52v software. Representative cell migrating fields are shown (n=3). Scale bar - 400 µm. Dots represent mean ± S.D. of n= 3 fields/well of three wells of three independent experiments. Nonparametric Student's t-test statistical analysis was performed. **(B)** Co-culture with astrocytes enhances the sprouting of melanoma 3D multicellular spheroid (A375, B16-F10, and B2905 mCherry-labeled melanoma cells). Mouse astrocytes were unlabeled. Human astrocytes were GFP-labeled in green. Dots represent melanoma cell invasion as fluorescence signal of mean ± S.D. of n=3 spheroids/ well of n=3 wells of three independent experiments (representative image of one independent experiment). Nonparametric Student's two sided t-test statistical analysis was performed. Scale bar - 400 µm.

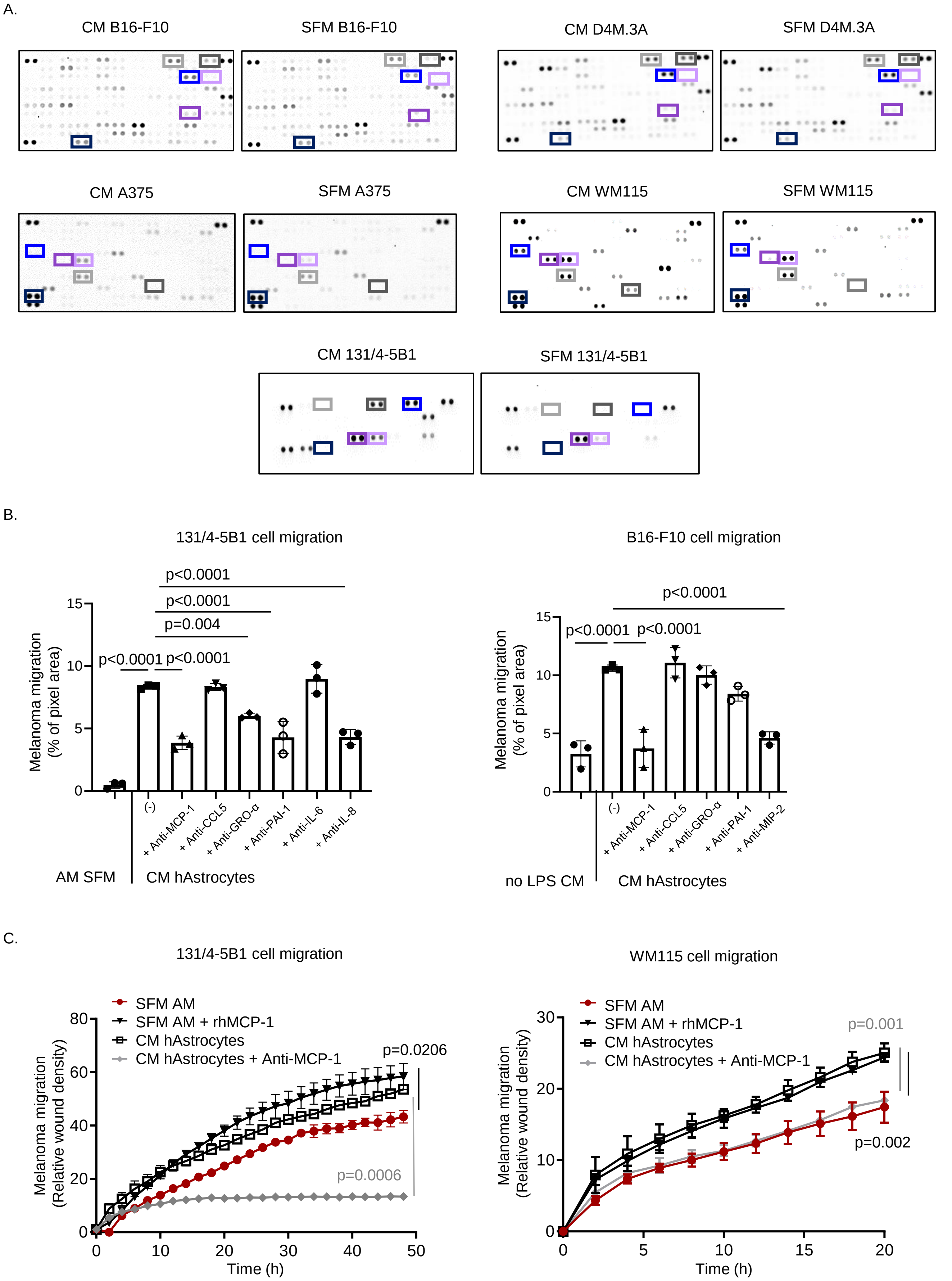
A.
CM B16-F10
SFM B16-F10
CM D4M.3A
SFM D4M.3A
CM A375
SFM A375
CM WM115
SFM WM115
CM 131/4-5B1
SFM 131/4-5B1
B.
131/4-5B1 cell migration
Melanoma migration (% of pixel area)
p<0.0001
p<0.0001
p=0.004
p<0.0001
p<0.0001
(-)
+ Anti-MCP-1
+ Anti-CCL5
+ Anti-GRO-α
+ Anti-PAI-1
+ Anti-IL-6
+ Anti-IL-8
AM SFM
CM hAstrocytes
B16-F10 cell migration
Melanoma migration (% of pixel area)
p<0.0001
p<0.0001
p<0.0001
(-)
+ Anti-MCP-1
+ Anti-CCL5
+ Anti-GRO-α
+ Anti-PAI-1
+ Anti-MIP-2
no LPS CM
CM hAstrocytes
C.
131/4-5B1 cell migration
SFM AM
SFM AM + rhMCP-1
CM hAstrocytes
CM hAstrocytes + Anti-MCP-1
p=0.0206
p=0.0006
Melanoma migration (Relative wound density)
Time (h)
WM115 cell migration
SFM AM
SFM AM + rhMCP-1
CM hAstrocytes
CM hAstrocytes + Anti-MCP-1
p=0.001
p=0.002
Melanoma migration (Relative wound density)
Time (h)

Supplemental Figure 7. MCP-1 is a key astrocytes-secreted upon melanoma cells-astrocytes interactions. (A) Representative array membranes of AS grown in melanoma CM vs melanoma SFM (B16-F10, D4M.3A, A375, WM115, and 131/4-5B1). MCP-1 in gray, RANTES in dark gray, GRO-α in blue, PAI-1 in dark blue, IL-6 in purple, and IL-8/MIP-2 in light purple. **(B)** Melanoma cells (131/4-5B1 and B16-F10) were allowed to migrate in the presence of SFM, astrocytes CM in the presence or absence of neutralizing antibodies. Astrocytes CM without LPS and astrocytes CM was used as negative control for cell migration in B16-F10 and 131/4-5B1, respectively. Mean values ± S.D. of triplicates of three independent biological repeats. One-way ANOVA statistical analysis. **(C)** Wound healing migration assay of WM115 and 131/5B1 cells grown in the presence of astrocytes SFM (red circle), astrocytes CM (white square), rhMCP-1 (black triangle) in astrocytes SFM, and MCP-1 nAb (grey diamond) in astrocytes CM. Dots represent mean ± S.D. of n= 2 field per well of triplicates of biological replicates. Two-way ANOVA statistical analysis.

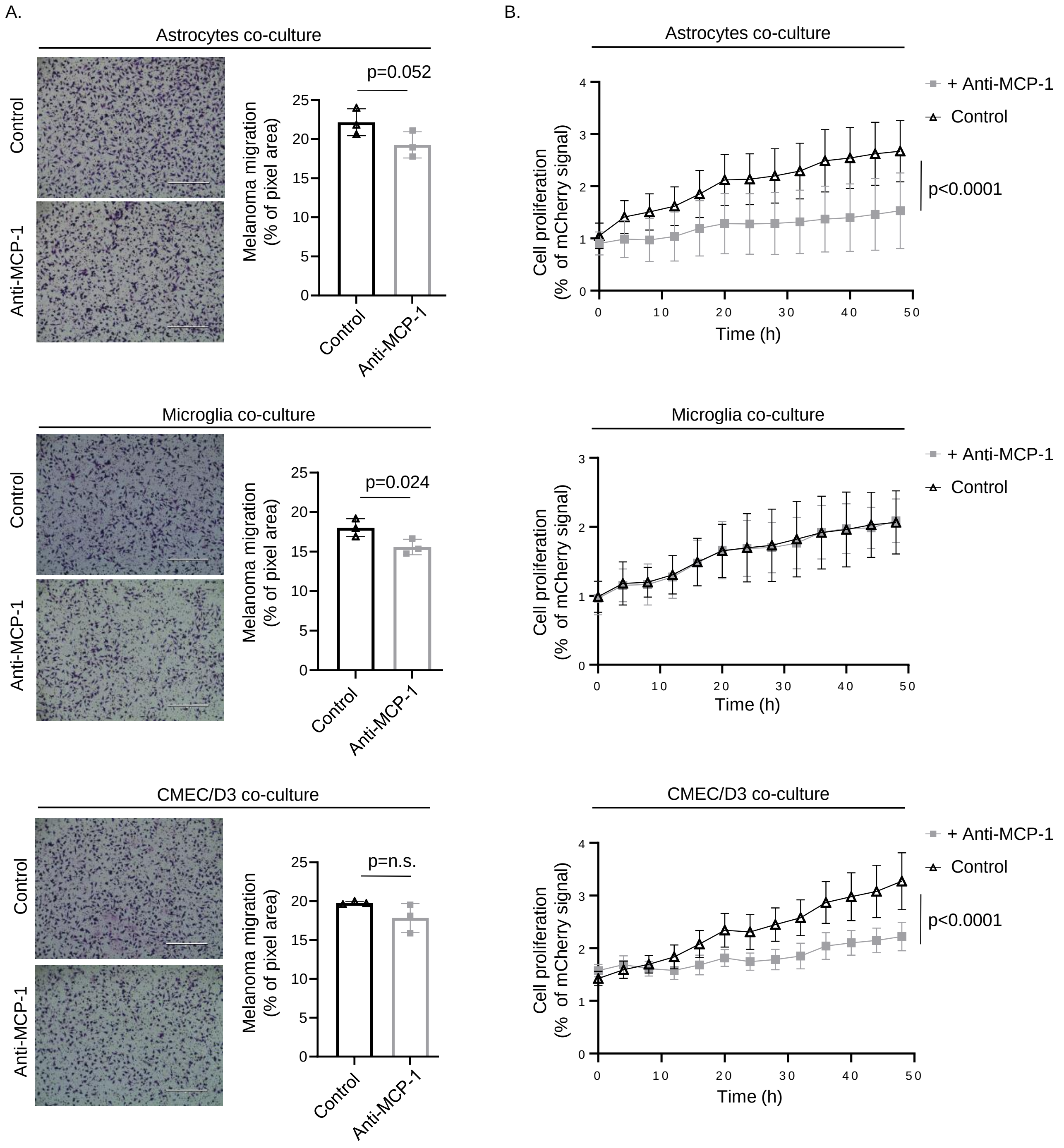

A.
B.
Astrocytes co-culture
Control
Anti-MCP-1
p=0.052
Melanoma migration (% of pixel area)
Cell proliferation (% of mCherry signal)
Time (h)
+ Anti-MCP-1
p<0.0001
Microglia co-culture
p=0.024
CMEC/D3 co-culture
p=n.s.

Supplemental Figure 8. MCP-1 neutralization in astrocytes is pivotal for melanoma cell migration and proliferation. (A) Migration of WM115 melanoma cells in Transwell® in co-culture with astrocytes, or microglia, or CMEC/D3 treated with anti-MCP-1 neutralizing antibody for 12 h. Pixel density (% of total area covered per field) of the migrated cancer cells. Representative cell migrating fields are shown. Scale bar - 400 µm. Mean ± S.D. of n= 3 fields/ well of triplicates of biological replicates. Nonparametric Student's t-test statistical analysis. **(B)** WM115 melanoma cell proliferation in co-culture with astrocytes and CMEC/D3 in 48 h following neutralization of MCP-1. Mean ± S.D. fluorescence signal of mCherry-labeled melanoma, measured by Incucyte™ live cell imaging. Mean ± S.D. of n= 4 fields per well of triplicates of biological replicates. Two-way ANOVA statistical analysis.

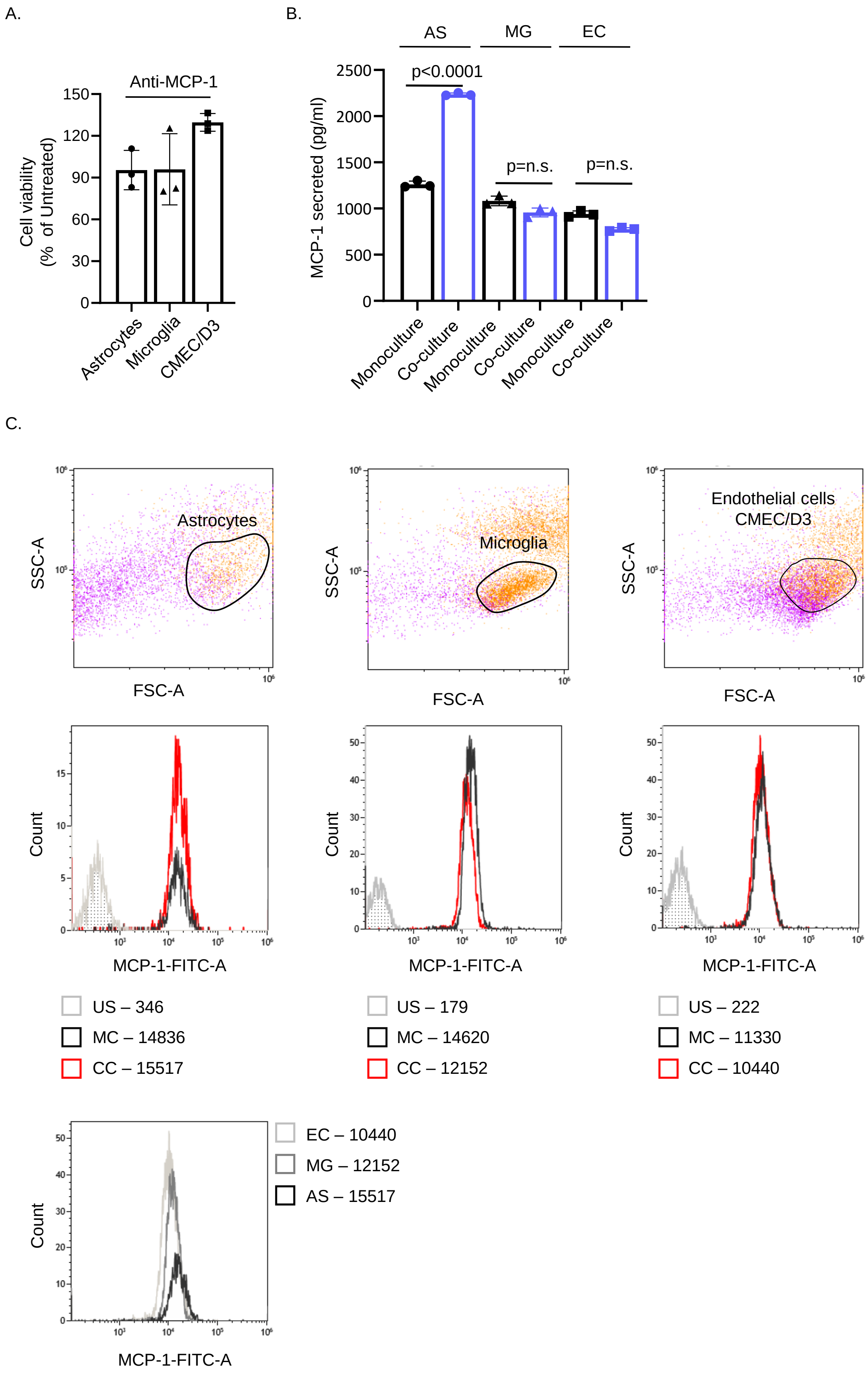
A.
Anti-MCP-1
Cell viability
(% of Untreated)
150
120
90
60
30
0
Astrocytes
Microglia
CMEC/D3
B.
AS
MG
EC
p<0.0001
p=n.s.
p=n.s.
MCP-1 secreted (pg/ml)
2500
2000
1500
1000
500
0
Monoculture
Co-culture
Monoculture
Co-culture
Monoculture
Co-culture
C.
Astrocytes
Microglia
Endothelial cells
CMEC/D3
SSC-A
FSC-A
Count
MCP-1-FITC-A
US – 346
MC – 14836
CC – 15517
US – 179
MC – 14620
CC – 12152
US – 222
MC – 11330
CC – 10440
EC – 10440
MG – 12152
AS – 15517

Supplemental Figure 9. Melanoma cells induces the expression of MCP-1 in bran resident cells and remarkable secretion in astrocytes. (A) Brain resident cells (astrocytes - circle, microglia - triangle, CMEC/D3 - square) were exposed to 10 µg/ml anti-MCP-1 nAb for 72 h. Cell viability was assessed by metabolic activity MTT assay. Mean ± S.D. of triplicates of biological replicates. Nonparametric Student's t-test statistical analysis. **(B)** MCP-1 secretion in astrocytes (AS), microglia (MG), and CMEC/D3 (EC) grown as monoculture in SFM, or in co-culture with WM115 melanoma cells for 24h. Mean ± S.D. of triplicates of biological replicates. Nonparametric Student's t-test statistical analysis. **(C)** Flow cytometry analysis of MCP-1 intracellular expression in astrocytes (AS), or microglia (MG), or CMEC/D3 (EC) grown in co-culture with WM115 melanoma cells (in red), or grown in monoculture (in black) for 24 h. The mean fluorescent intensity (MFI) of unstained control (US – gray dots), monoculture (MC – in black), and co-culture (CC – in red) of melanoma and brain resident cells, is shown. The FSC and SCC of the gated cell population is presented (n=3).

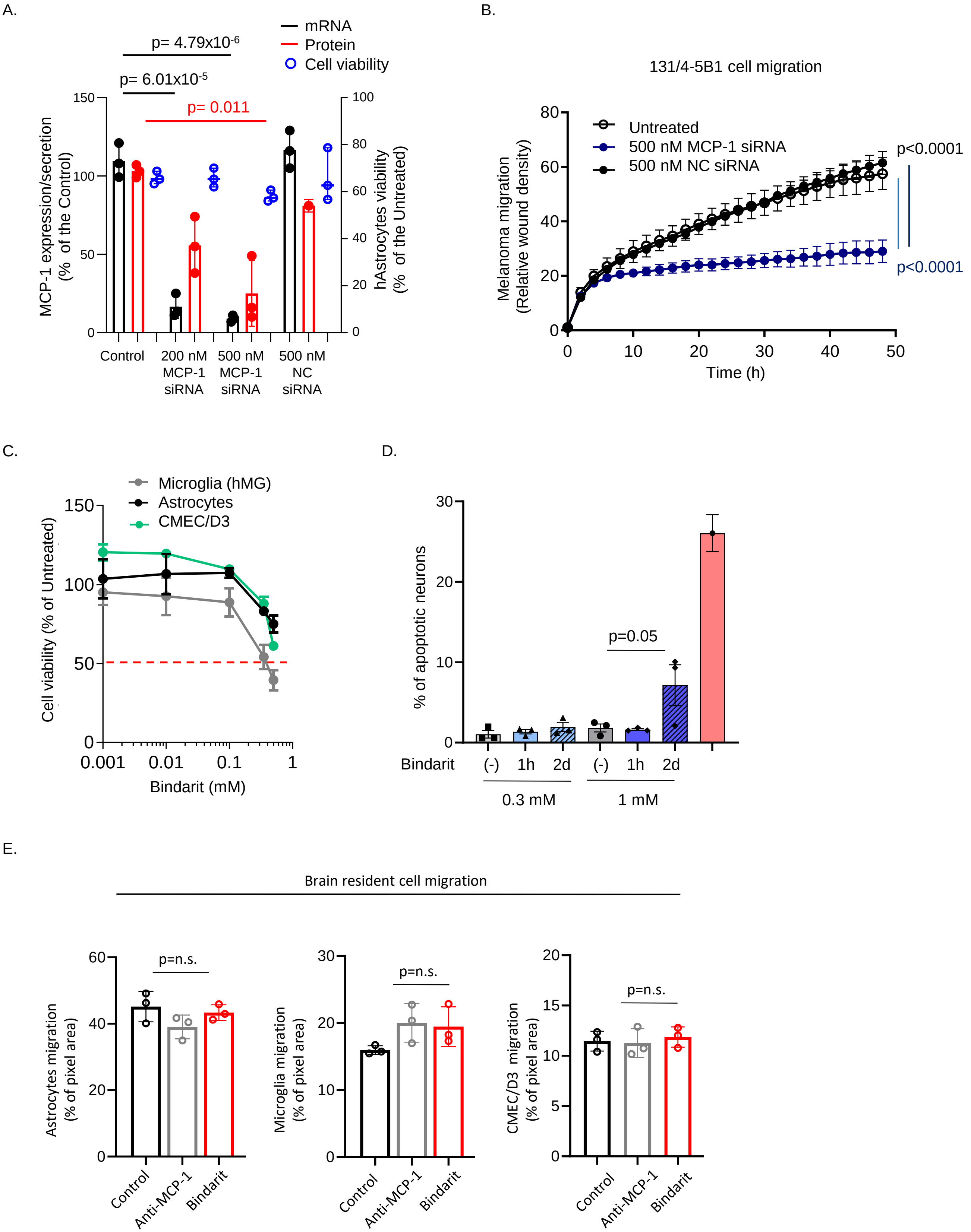
A.
mRNA
Protein
Cell viability
p= 4.79x10-6
p= 6.01x10-5
p= 0.011
MCP-1 expression/secretion (% of the Control)
hAstrocytes viability (% of the Untreated)
Control
200 nM MCP-1 siRNA
500 nM MCP-1 siRNA
500 nM NC siRNA
B.
131/4-5B1 cell migration
Untreated
500 nM MCP-1 siRNA
500 nM NC siRNA
p<0.0001
p<0.0001
Melanoma migration (Relative wound density)
Time (h)
C.
Microglia (hMG)
Astrocytes
CMEC/D3
Cell viability (% of Untreated)
Bindarit (mM)
D.
% of apoptotic neurons
p=0.05
Bindarit
(-)
1h
2d
(-)
1h
2d
0.3 mM
1 mM
E.
Brain resident cell migration
Astrocytes migration (% of pixel area)
Microglia migration (% of pixel area)
CMEC/D3 migration (% of pixel area)
p=n.s.
p=n.s.
p=n.s.
Control
Anti-MCP-1
Bindarit

Supplemental Figure 10. Silencing of MCP-1 in astrocytes reduces melanoma migration without affecting cell viability. (A) Following 24h incubation with siRNA:PEI targeting MCP-1, or NC:PEI in astrocytes medium. MCP-1 mRNA expression in astrocytes was significantly reduced to 20% and 12% compared to control or NC in a siRNA concentration-dependent manner (black bars), without affecting the cell viability of human astrocytes (blue dots). MCP-1 silencing also reduced the secreted protein levels to 46% and 30% compared to control or NC in a siRNA concentration-dependent manner (red bars). Mean ± S.D. of triplicates of three independent experiments. One-way ANOVA statistical analysis. **(B)** Wound healing migration assay of 131/4-5B1 cells grown in the presence of astrocytes CM (untreated – black empty dot), or siRNA:PEI targeting MCP-1 (in blue), or NC (black full dot). Dots represent mean ± S.D. of n= 2 field per well of triplicates of biological replicates. Two-way ANOVA statistical analysis was performed. **(C)** Microglia – in gray, astrocytes – in black, and CMEC/D3 –in green exposed to bindarit for 72 h. Cell viability was assessed by metabolic activity MTT assay. Mean ± S.D. of triplicates of three independent biological repeats. Nonparametric Student's t-test statistical analysis. **(D)** Percentage of apoptotic cells in primary hippocampal neurons treated with 0.3 and 1 mM vehicle (in gray), or bindarit (in blue) for 1 h (plain bars) and 2 days (pattern bars) or 30 minutes with 20 µM carbonyl cyanide 4-phenylhydrazone - FCCP (positive control for cell death in pink). n=3 coverslips from 2 independent experiments and n=1 for the positive control. Average of the percentage of apoptotic cells in 4 fields of view. Nested One-way ANOVA, Tukey’s Multiple Comparison test. Mean ± SEM. (E) Transwell® migration of brain resident cells (astrocytes, microglia, CMEC/D3) treated with anti-MCP-1 nAb (in gray), or 0.3 mM bindarit (in red) for 24 h compared to untreated control (in black). Mean ± S.D. of n=3 fields/well of biological replicates. Nonparametric Student's t-test statistical analysis.

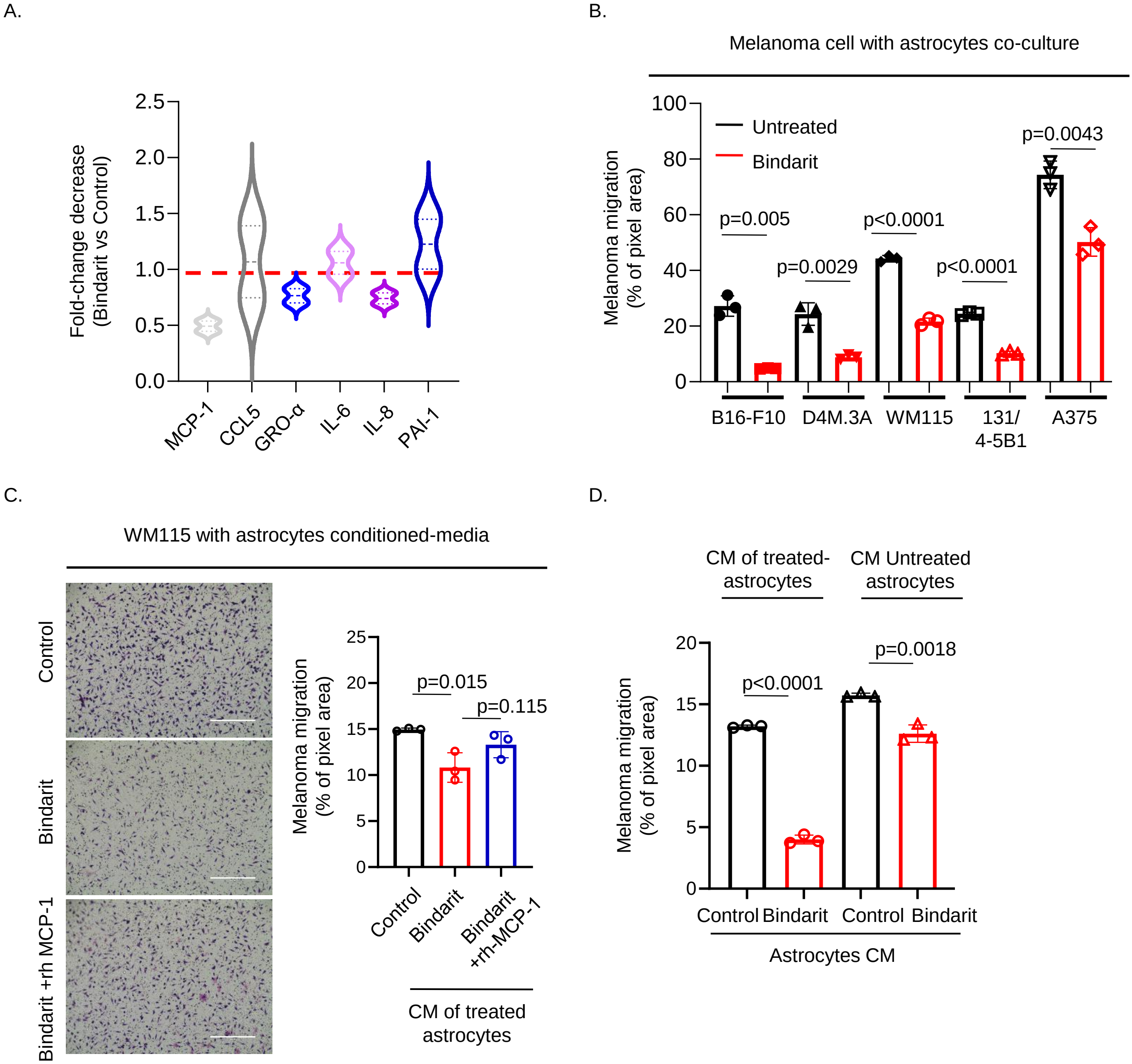
A.
Fold-change decrease (Bindarit vs Control)
MCP-1
CCL5
GRO-α
IL-6
IL-8
PAI-1
B.
Melanoma cell with astrocytes co-culture
Untreated
Bindarit
Melanoma migration (% of pixel area)
p=0.005
p=0.0029
p<0.0001
p<0.0001
p=0.0043
B16-F10
D4M.3A
WM115
131/4-5B1
A375
C.
WM115 with astrocytes conditioned-media
Control
Bindarit
Bindarit +rh MCP-1
Melanoma migration (% of pixel area)
p=0.015
p=0.115
Control
Bindarit
Bindarit +rh-MCP-1
CM of treated astrocytes
D.
CM of treated-astrocytes
CM Untreated astrocytes
Melanoma migration (% of pixel area)
p<0.0001
p=0.0018
Control
Bindarit
Control
Bindarit
Astrocytes CM

Supplemental Figure 11. Bindarit selectively inhibits MCP-1 secretion and melanoma cell migration (A) Levels of the selected six pro-inflammatory cytokines secreted by astrocytes in SFM following bindarit treatment for 24 h. Results are expressed as fold change secretion. Mean of two independent biological repeats. **(B)** Melanoma cells (B16-F10, D4M.3A, WM115, 131/4-5B1, A375) were co-culture with astrocytes (in black) and treated with 0.3 mM bindarit (in red) in a Transwell® assay for 6 to 24 h. Pixel density (% of total area covered per field) of the migrated cancer cells was quantified using ImageJ 1.52v software. Mean ± S.D. of triplicates of three independent biological repeats. Nonparametric Student's t-test statistical analysis was performed. **(C)** Transwell® migration of melanoma WM115 cells towards CM of bindarit-treated astrocytes (in red), or towards untreated astrocytes CM (in black), or towards CM of bindarit-treated astrocytes supplemented with rh-MCP-1 in blue. Representative fields of WM115 migrated cells. Scale bar - 400 µm. Mean ± S.D. of triplicates of biological replicates. One-way ANOVA statistical analysis. **(D)** D4M.3A Transwell® migration towards bindarit-treated astrocytes or towards astrocytes CM supplemented with 0.3 mM bindarit. Mean ± S.D. of triplicates of three independent biological repeats. Nonparametric Student's t-test statistical analysis.

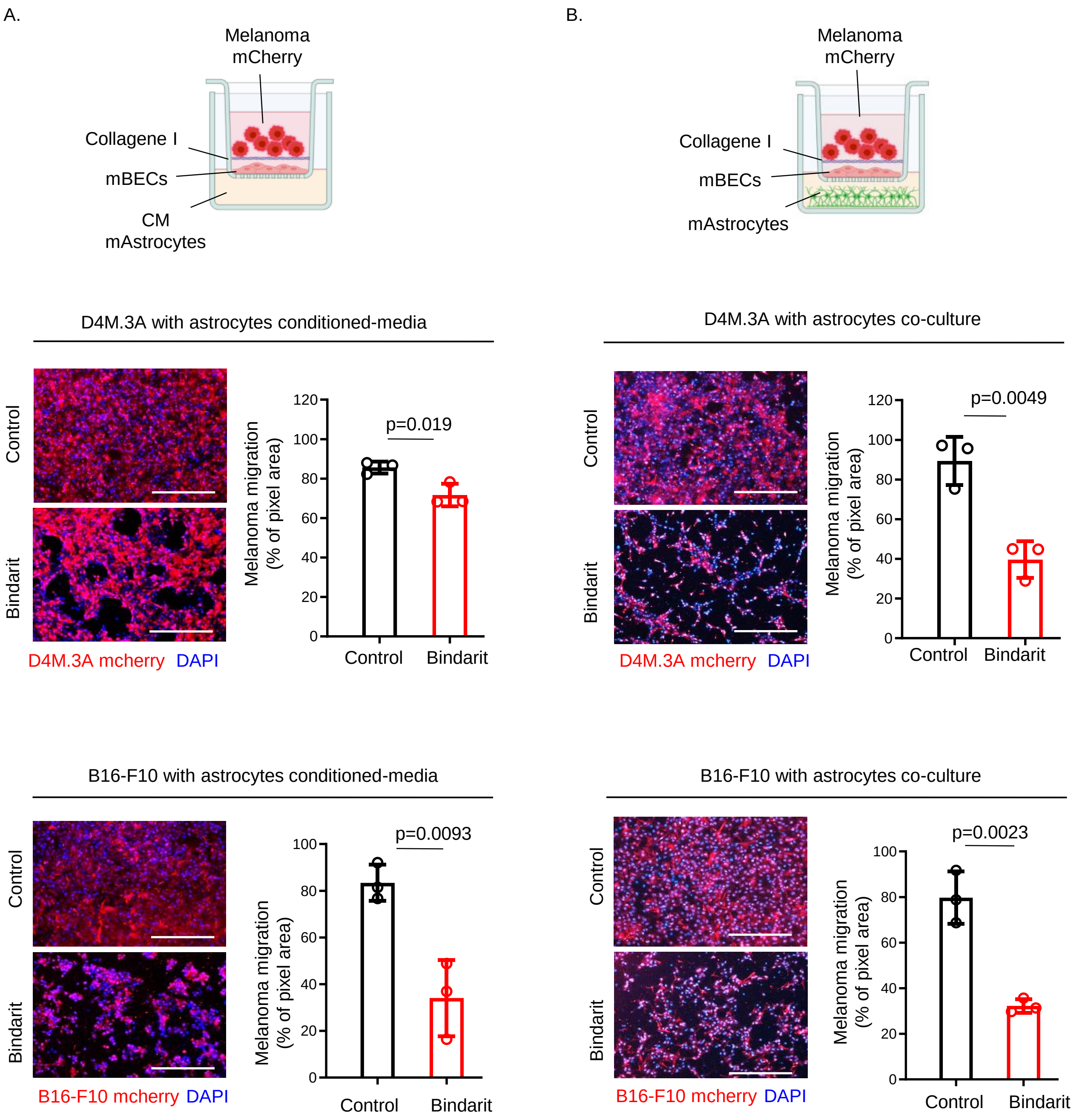

A.
Melanoma
mCherry
Collagene I
mBECs
CM
mAstrocytes
B.
Melanoma
mCherry
Collagene I
mBECs
mAstrocytes
D4M.3A with astrocytes conditioned-media
Control
Bindarit
D4M.3A mcherry DAPI
Melanoma migration
(% of pixel area)
120
100
80
60
40
20
0
p=0.019
Control
Bindarit
D4M.3A with astrocytes co-culture
Control
Bindarit
D4M.3A mcherry DAPI
Melanoma migration
(% of pixel area)
120
100
80
60
40
20
0
p=0.0049
Control
Bindarit
B16-F10 with astrocytes conditioned-media
Control
Bindarit
B16-F10 mcherry DAPI
Melanoma migration
(% of pixel area)
100
80
60
40
20
0
p=0.0093
Control
Bindarit
B16-F10 with astrocytes co-culture
Control
Bindarit
B16-F10 mcherry DAPI
Melanoma migration
(% of pixel area)
100
80
60
40
20
0
p=0.0023
Control
Bindarit

Supplemental Figure 12. Melanoma cell transendothelial migration is decreased following MCP-1 inhibition (A-B) Migration of melanoma mCherry-labeled D4M.3A cells towards astrocytes CM **(A)** or towards astrocytes **(B)** in a transendothelial migration assay. A schematic representation of the Transwell® assay is presented (mBECs – murine brain endothelial cells). Pixel density (% of the total area covered per field). Representative fields of mCherry-labeled D4M.3A cells. Scale bar - 400 µm. Mean ± S.D. of triplicates of three independent biological repeats. Nonparametric Student's t-test statistical analysis.

A.

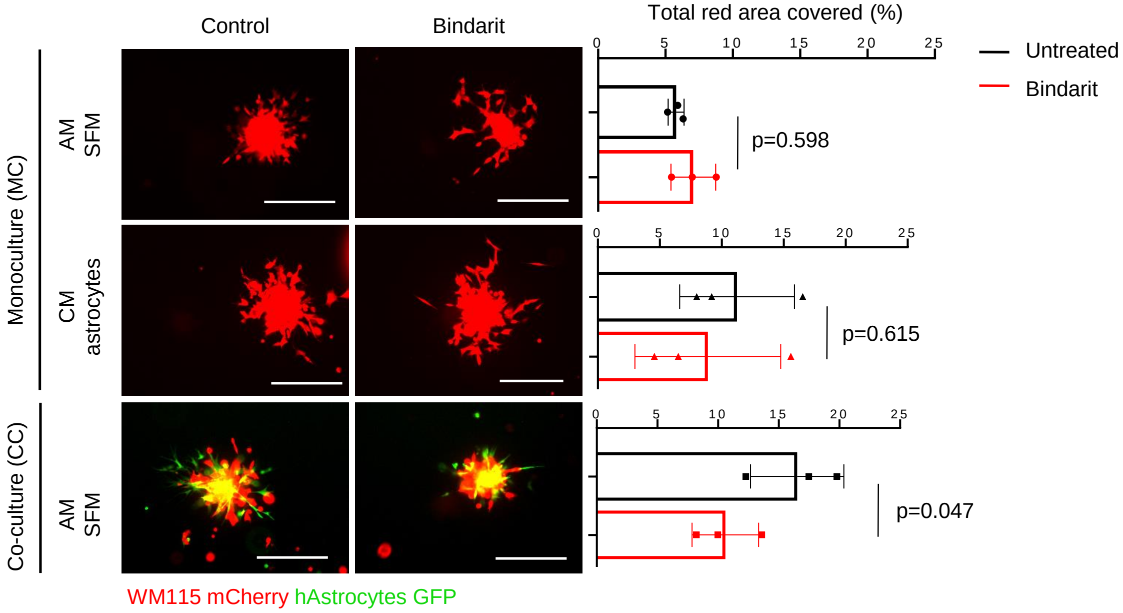
Control
Bindarit
Total red area covered (%)
0
5
10
15
20
25
Untreated
Bindarit
Monoculture (MC)
AM SFM
p=0.598
CM astrocytes
p=0.615
Co-culture (CC)
AM SFM
p=0.047
WM115 mCherry hAstrocytes GFP

B.

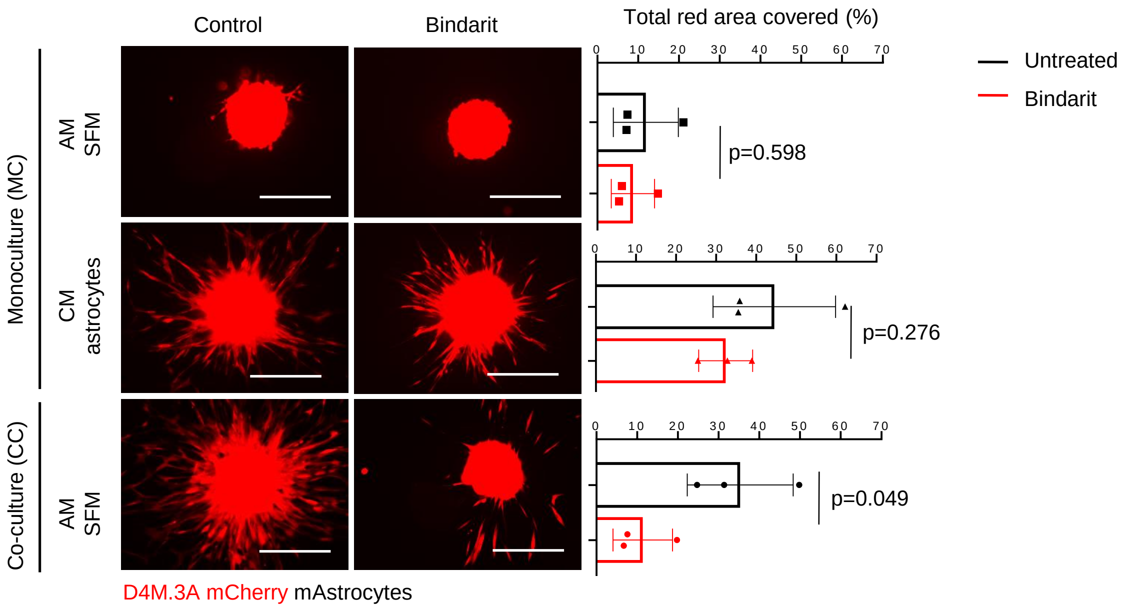
Control
Bindarit
Total red area covered (%)
0
10
20
30
40
50
60
70
Untreated
Bindarit
Monoculture (MC)
AM SFM
p=0.598
CM astrocytes
p=0.276
Co-culture (CC)
AM SFM
p=0.049
D4M.3A mCherry mAstrocytes

Supplemental Figure 13. Melanoma 3D invasion in co-culture with astrocytes is decreased following MCP-1 inhibition (A-B) 3D Matrigel® invasion of melanoma **(A)** WM115 mCherry cells or **(B)** D4M.3A mCherry cells monoculture grown in astrocytes SFM, in astrocytes CM or co-cultured with astrocytes (in green or unlabeled). mCherry-labeled cell sprouting (in red) % of the total area covered per one spheroid (untreated control – in black, bindarit-treated spheroids – in red). Representative cell migrating fields are shown (n=3). Mean ± S.D. of triplicates of three independent biological repeats. Nonparametric Student's t-test statistical analysis. Scale bar - 400 µm.

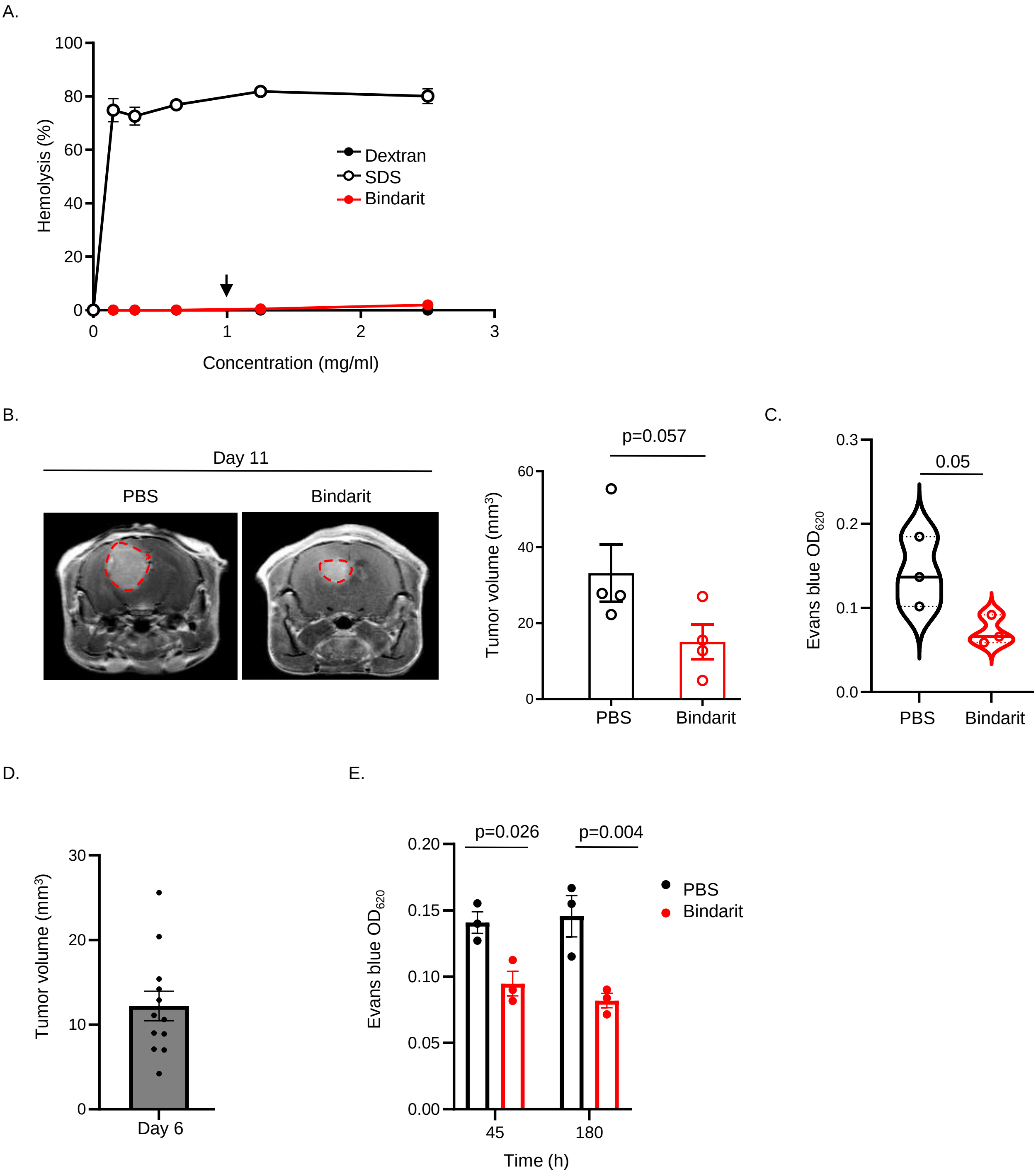
A.
100
80
60
40
20
0
Hemolysis (%)
Dextran
SDS
Bindarit
0
1
2
3
Concentration (mg/ml)
B.
Day 11
PBS
Bindarit
p=0.057
60
40
20
0
Tumor volume (mm3)
PBS
Bindarit
C.
0.3
0.2
0.1
0.0
0.05
Evans blue OD620
PBS
Bindarit
D.
30
20
10
0
Tumor volume (mm3)
Day 6
E.
0.20
0.15
0.10
0.05
0.00
p=0.026
p=0.004
Evans blue OD620
PBS
Bindarit
45
180
Time (h)

Supplemental Figure 14. Inhibition of MCP-1 decreases MCP-1-induced vessel hyper-permeability in MBM tumors. **(A)** Hemolysis assay of bindarit at the concentration administered in vivo (1 mg/ml). Dextran and SDS were used as negative and positive controls for hemolysis, respectively. Mean ± S.D. of one representative experiment of three independent biological repeats. **(B)** Representative MRI scans at day 11 (n=4 per group) and quantification of tumor size. Mean ± S.E.M. are presented (n=4). Nonparametric Student's t-test statistical analysis. **(C)** Distribution of relative absorbance of Evans blue dye in bindarit-treated group compared to PBS-treated group. Mean distribution of n=3 mice per group. **(D)** Tumor size in MRI scans on day 6 following B16-F10 intracranially inoculated cells. n=12 mice randomized in 2 groups for 2 endpoint (n=3 mice per group). **(E)** Distribution of relative absorbance of Evans blue dye in bindarit-treated group compared to PBS-treated group on day 7 and 8, and measured on day 8 at 45’ and 3 h post-treatment. Mean ± S.E.M. distribution of n=3 mice per group. Nonparametric Student's t-test statistical analysis.

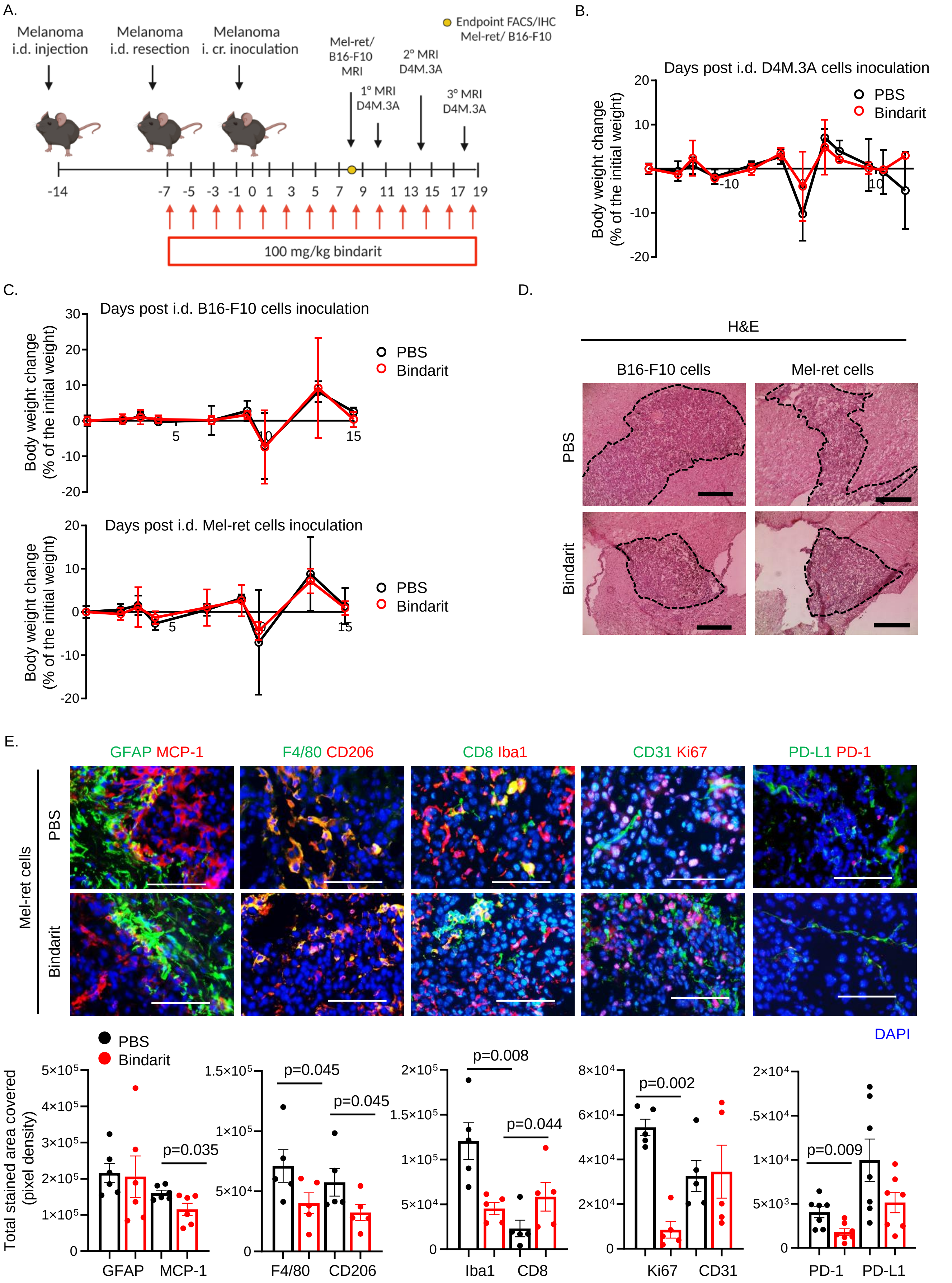
A.
Melanoma i.d. injection
Melanoma i.d. resection
Melanoma i. cr. inoculation
Mel-ret/ B16-F10 MRI
1° MRI D4M.3A
2° MRI D4M.3A
3° MRI D4M.3A
Endpoint FACS/IHC Mel-ret/ B16-F10
-14 -7 -5 -3 -1 0 1 3 5 7 9 11 13 15 17 19
100 mg/kg bindarit
B.
Days post i.d. D4M.3A cells inoculation
Body weight change (% of the initial weight)
PBS
Bindarit
C.
Days post i.d. B16-F10 cells inoculation
Days post i.d. Mel-ret cells inoculation
D.
H&E
B16-F10 cells
Mel-ret cells
PBS
Bindarit
E.
GFAP MCP-1
F4/80 CD206
CD8 Iba1
CD31 Ki67
PD-L1 PD-1
Mel-ret cells
DAPI
Total stained area covered (pixel density)
p=0.035
p=0.045
p=0.045
p=0.008
p=0.044
p=0.002
p=0.009
GFAP
MCP-1
F4/80
CD206
Iba1
CD8
Ki67
CD31
PD-1
PD-L1

Supplemental Figure 15. Bindarit treatment improves CD8 T-cell infiltration and decreases immune co-inhibitory molecules. (A) Scheme of treatment. Briefly, D4M.3A, or B16-F10, or Mel-ret melanoma cells were i.d. inoculated into immunocompetent C57BL/6 mice to generate primary lesions (n=10 per cell line). Following PM resection (70-100 mm^3 lesions), mice were treated with PBS, or 100 mg/kg bindarit (QOD). Then, MBM were generated by intracranial cell inoculation according to the scheme of treatment. Created with BioRender.com. **(B)** Body weight change was monitored twice a week upon D4M.3A tumor resection. Mean ± S.E.M. of n=4 in bindarit-treated group, n=5 in PBS-treated group. **(C)** Body weight change was monitored twice a week upon B16-F10 or Mel-ret tumor resection. Mean ± S.D. of n=5 per group. **(D)** H&E staining for tumor morphology and size of B16-F10 or Mel-ret MBM at day 8 post cell inoculation. Scale bar - 400 µm. **(E)** Histological analysis at day 8 (representative fields of n=5-7 fields per marker in n=3 mice per group) of MCP-1/GFAP (in red and in green, respectively), CD206 staining associated to F4/80 macrophages (in red and in green, respectively), infiltration of $CD8^+$ T cells /Iba1 microglia/microphages (red/green), tumor proliferation (Ki67 – in red) and blood vasculature (CD31 – in green), PD-1/PD-L1 exhausted T cells/ inhibitory molecules (red/green) in Mel-ret MBM. Mean ± S.E.M. Nonparametric Student's t-test statistical analysis.

A.

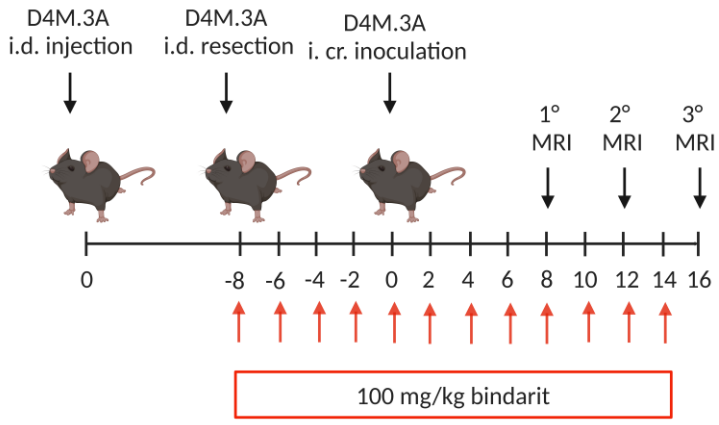
D4M.3A
i.d. injection
D4M.3A
i.d. resection
D4M.3A
i. cr. inoculation
1° MRI
2° MRI
3° MRI
0
-8
-6
-4
-2
0
2
4
6
8
10
12
14
16
100 mg/kg bindarit

B.

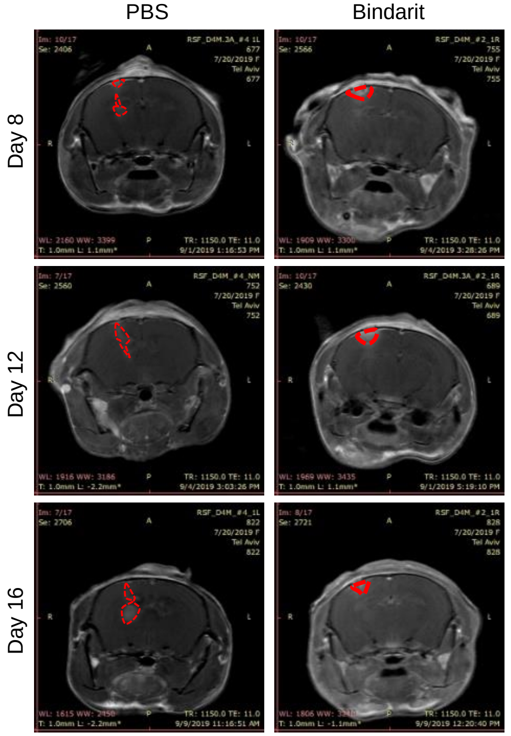
PBS
Bindarit
Day 8
Day 12
Day 16

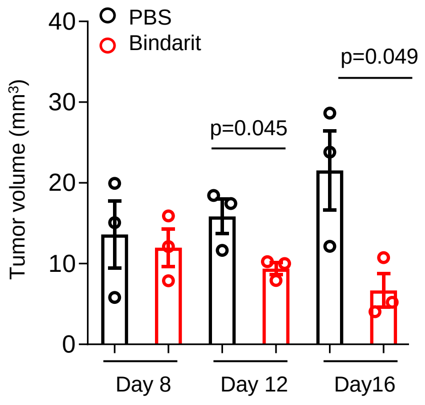
PBS
Bindarit
p=0.045
p=0.049
Tumor volume (mm^3)
40
30
20
10
0
Day 8
Day 12
Day16

C.

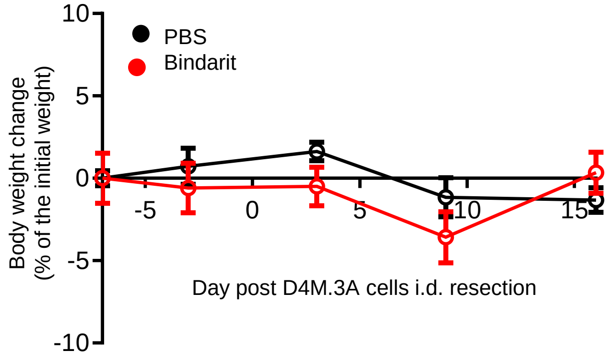
PBS
Bindarit
Body weight change
(% of the initial weight)
10
5
0
-5
-10
-5
0
5
10
15
Day post D4M.3A cells i.d. resection

Supplemental Figure 16. MCP-1 inhibition delays D4M.3A MBM progression. (A) D4M.3A melanoma cells were injected i.d. in immunocompetent C57BL/6 mice to generate primary lesions. After primary melanoma (PM) resection, mice were treated with 100 mg/kg bindarit (n=3), and intracranially injected with D4M.3A cells to generate MBM, according to the scheme of treatment. Created with BioRender.com. **(B)** Representative MRI scans and quantification tumor size of the mice bearing MBM at day 8, 12 and 16 are presented (n=3 per group). Mean ± S.E.M. Treatment with bindarit resulted in the inhibition of tumor growth, as shown by quantification dot plots of tumor size. **(C)** Mice body weight change was monitored twice a week upon D4M.3A tumor resection. Mean ± S.D. of n=3 per group.

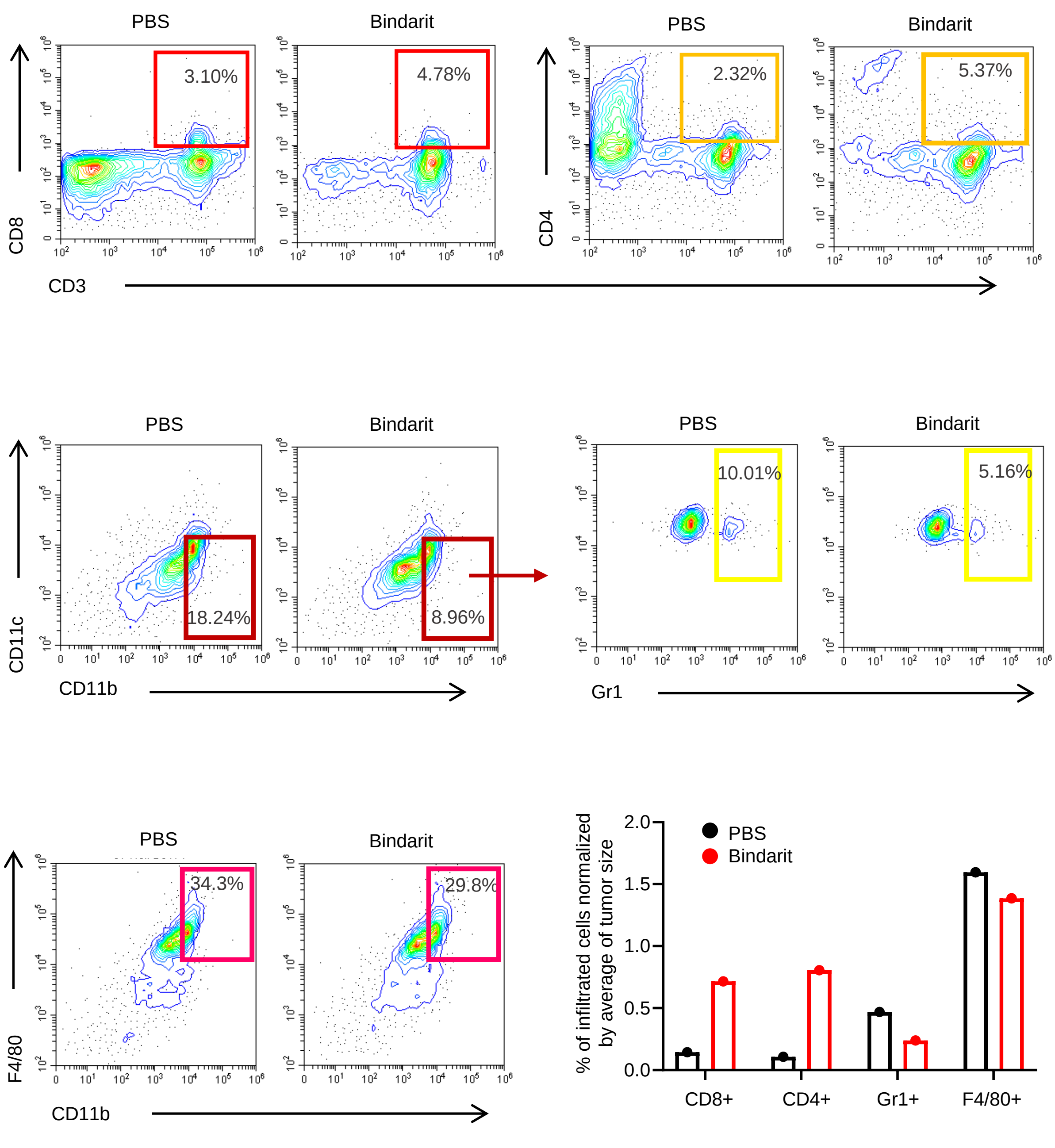
PBS
Bindarit
PBS
Bindarit
CD8
CD4
CD3
3.10%
4.78%
2.32%
5.37%
PBS
Bindarit
PBS
Bindarit
CD11c
CD11b
Gr1
18.24%
8.96%
10.01%
5.16%
PBS
Bindarit
F4/80
CD11b
34.3%
29.8%
% of infiltrated cells normalized by average of tumor size
PBS
Bindarit
CD8+
CD4+
Gr1+
F4/80+

Supplemental Figure 17. Bindarit treatment shows impaired myeloid-derived suppressor cell (MDSC) infiltration and increased anti-tumor T-cell recruitment in MBM. Flow cytometry of infiltrating immune cells in D4M.3A MBM on day 16 post intracranial melanoma cells inoculation. MCP-1 inhibition moderately enhanced the infiltration of cytotoxic $CD8^+$ T cells and $CD4^+$ T cells and decreased the levels of immune-suppressive cells, such as macrophages (CD11b/F4/80) and MDSCs and (CD11b/Gr1). n=2 mice per group. Representative FSC and SSC of the gated lymphocytes population is presented.

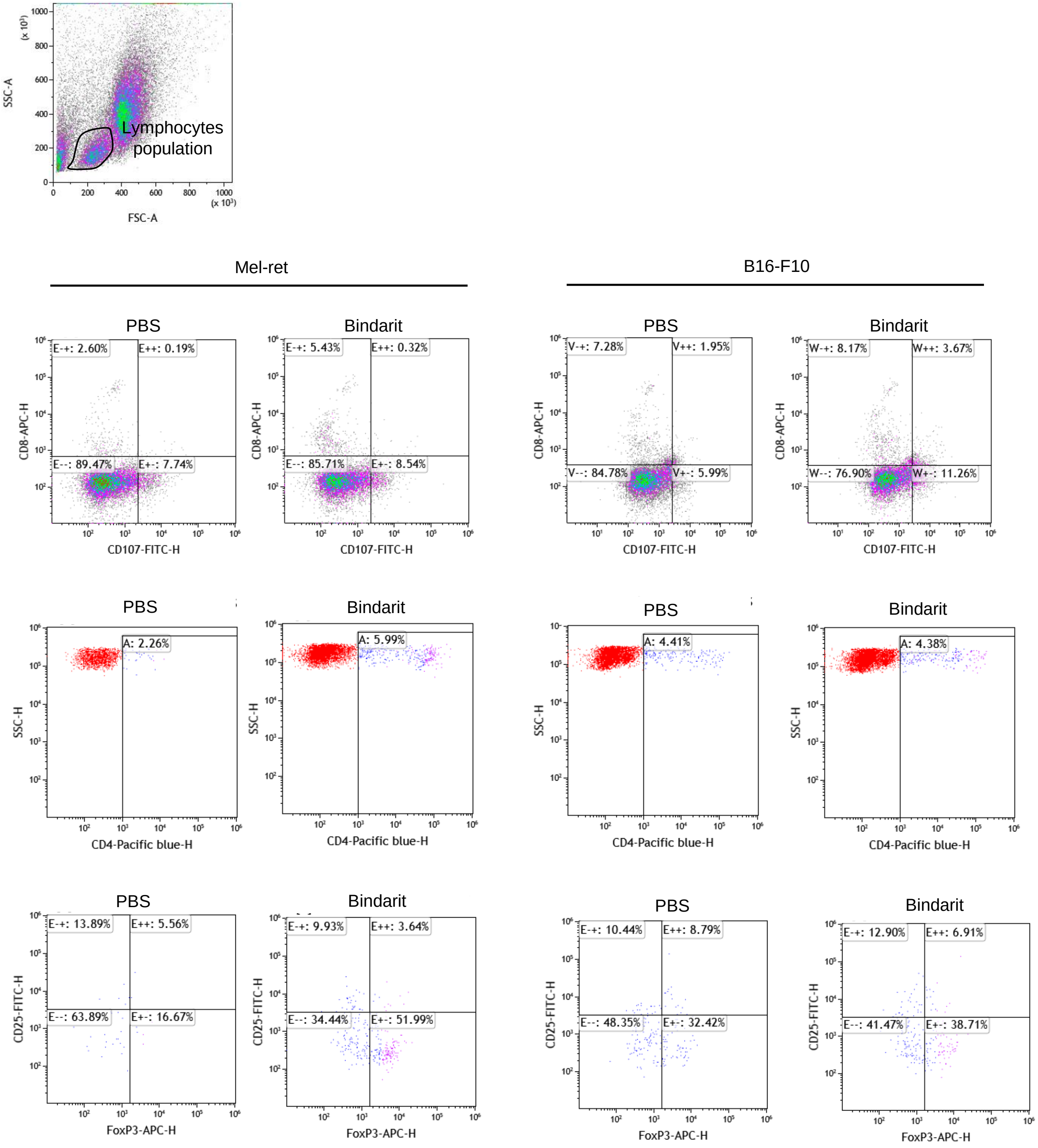

Lymphocytes population
SSC-A
FSC-A
Mel-ret
B16-F10
PBS
Bindarit
PBS
Bindarit
E-+: 2.60%
E++: 0.19%
E--: 89.47%
E+-: 7.74%
E-+: 5.43%
E++: 0.32%
E--: 85.71%
E+-: 8.54%
V-+: 7.28%
V++: 1.95%
V--: 84.78%
V+-: 5.99%
W-+: 8.17%
W++: 3.67%
W--: 76.90%
W+-: 11.26%
CD8-APC-H
CD107-FITC-H
PBS
Bindarit
PBS
Bindarit
A: 2.26%
A: 5.99%
A: 4.41%
A: 4.38%
SSC-H
CD4-Pacific blue-H
PBS
Bindarit
PBS
Bindarit
E-+: 13.89%
E++: 5.56%
E--: 63.89%
E+-: 16.67%
E-+: 9.93%
E++: 3.64%
E--: 34.44%
E+-: 51.99%
E-+: 10.44%
E++: 8.79%
E--: 48.35%
E+-: 32.42%
E-+: 12.90%
E++: 6.91%
E--: 41.47%
E+-: 38.71%
CD25-FITC-H
FoxP3-APC-H

Supplemental Figure 18. Tumor-infiltrating lymphocytes following treatment with bindarit in MBM. Flow cytometry of lymphocyte infiltration in Mel-ret MBM or B16-F10 MBM at day 8 post intracranial melanoma cells inoculation. The infiltration of CD8+-activated (CD107+) T cells was upregulated in bindarit treated-group, whereas suppressive CD4-Tregs were decreased. n=2 per group. Representative FSC and SSC of the gated lymphocytes population is presented.

A.

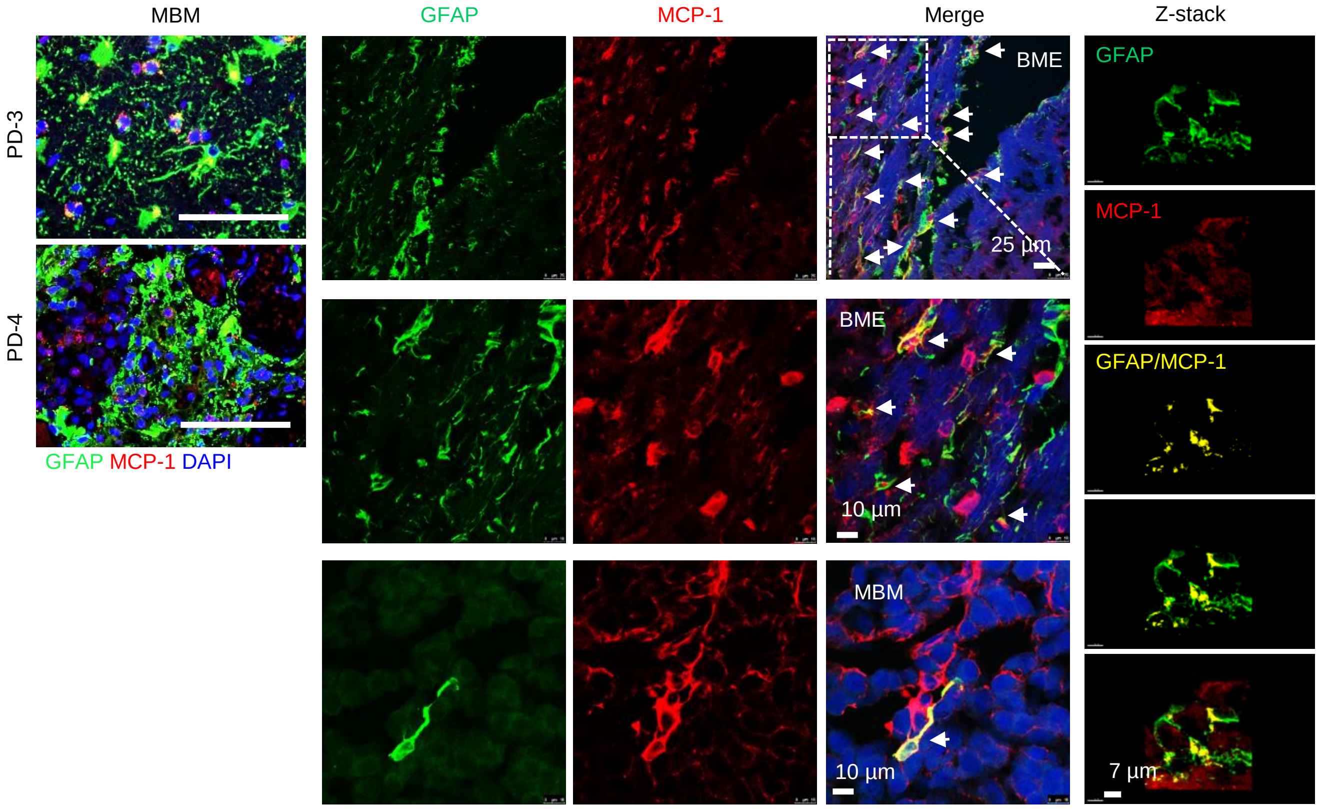
MBM
GFAP
MCP-1
Merge
Z-stack
PD-3
PD-4
GFAP MCP-1 DAPI
BME
25 µm
BME
10 µm
MBM
10 µm
GFAP
MCP-1
GFAP/MCP-1
7 µm

B.

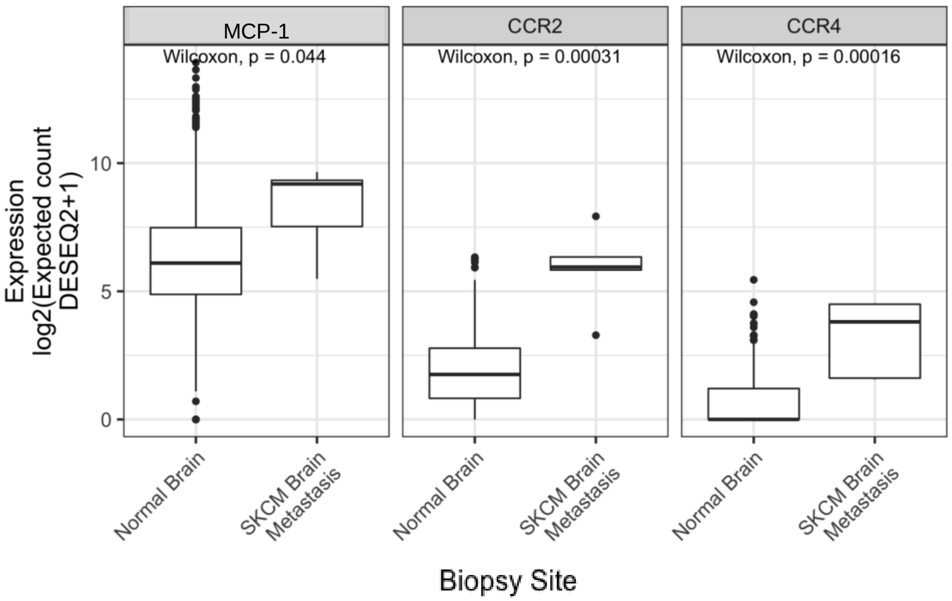
MCP-1
Wilcoxon, p = 0.044
CCR2
Wilcoxon, p = 0.00031
CCR4
Wilcoxon, p = 0.00016
Expression
log2(Expected count
DESEQ2+1)
10
5
0
Normal Brain
SKCM Brain Metastasis
Normal Brain
SKCM Brain Metastasis
Normal Brain
SKCM Brain Metastasis
Biopsy Site

Supplemental Figure 19. Astrocytes' activation and MCP-1 secretion may trigger CCR2/CCR4 overexpression in MBM. (A) Representative images of GFAP/MCP-1 (in green/red, respectively) staining in FFPE samples of human patients bearing MBM (PD-3 and PD-4). Scale bar - 100 µm. Confocal images of GFAP (in green), MCP-1 (in red), and their co-localization (in yellow and white arrows) in B16-F10 MBM and in the BME. Scale bar – 25 and 10 µm. Z-stack: representative images of 3-dimentional co-localization (in yellow) of GFAP (in green) and MCP1 (in red) performed using confocal Z-stacks images obtained at X60 magnification. Scale bar - 7µm. **(B)** Expression profile of MCP-1 (CCL2), CCR2, and CCR4 in melanoma brain metastasis (n=5 from TCGA) compared to their expression in normal brain (n=1148 from GTex). *p*-value comparing the expression median across groups are two-sided and computed using Wilcoxon rank-sum test. The boxplot shows the expression distribution, where the center line denotes the median, the box edges indicate the interquartile range and the black line represents the rest of the distribution.

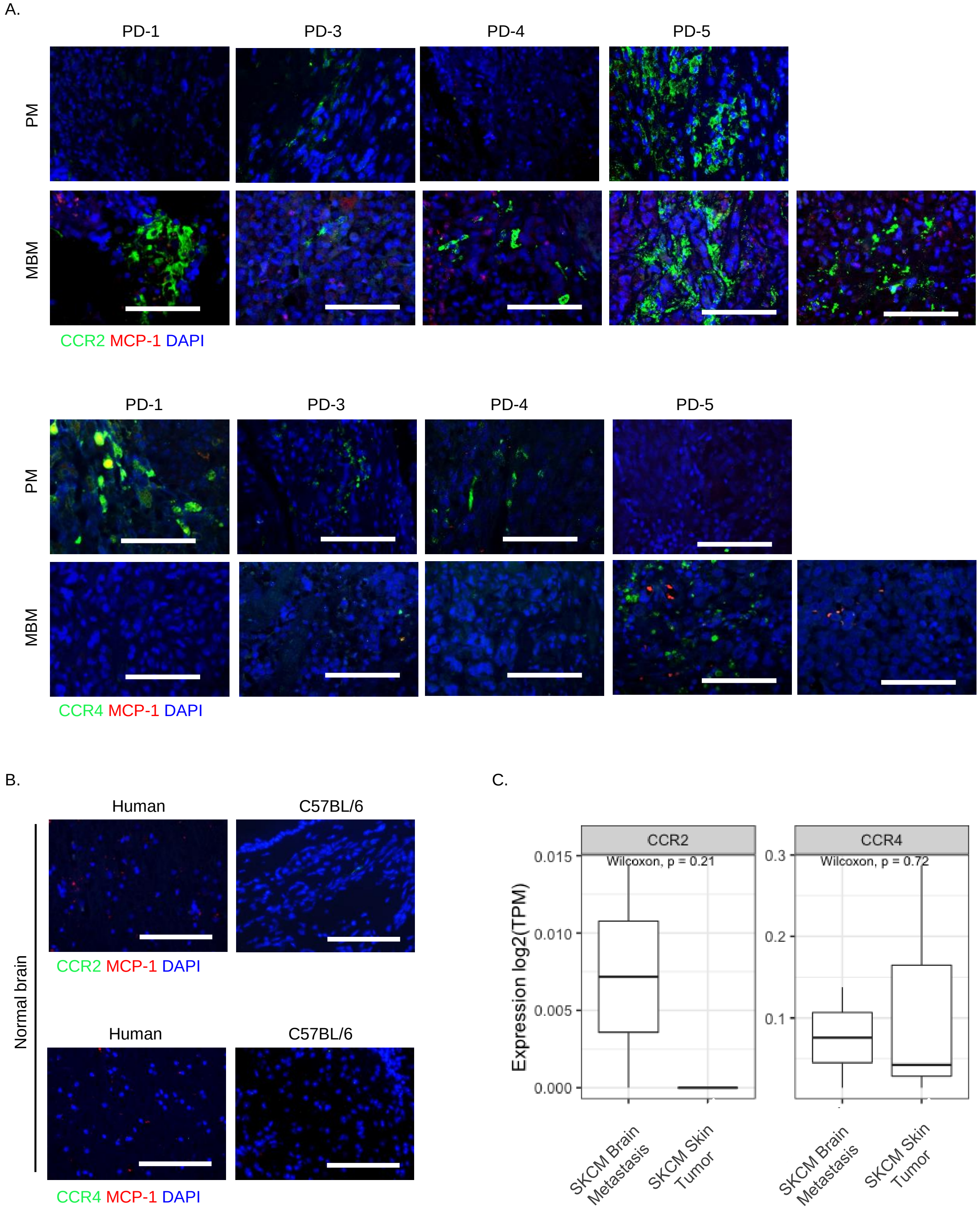
A.
PD-1
PD-3
PD-4
PD-5
PM
MBM
CCR2 MCP-1 DAPI
PD-1
PD-3
PD-4
PD-5
PM
MBM
CCR4 MCP-1 DAPI
B.
Human
C57BL/6
Normal brain
CCR2 MCP-1 DAPI
Human
C57BL/6
CCR4 MCP-1 DAPI
C.
CCR2
Wilcoxon, p = 0.21
CCR4
Wilcoxon, p = 0.72
Expression log2(TPM)
0.015
0.010
0.005
0.000
0.3
0.2
0.1
SKCM Brain Metastasis
SKCM Skin Tumor
SKCM Brain Metastasis
SKCM Skin Tumor

Supplemental Figure 20. CCR2/CCR4 overexpression in MBM. (A) Representative fields of MCP-1/CCR2 (in green/red, respectively) and MCP-1/CCR4 (in green/red, respectively) staining in PD-1, PD-3-5 samples of MBM. Scale bar - 100 µm. **(B)** Representative fields of MCP-1/CCR2 (in green/red, respectively) and MCP-1/CCR4 (in green/red, respectively) staining in healthy brain samples of human and mouse (C57BL/6 strain). Scale bar - 100 µm. **(C)** Expression profile of CCR2/4 (y-axis) in CCLE/DepMap of melanoma cell from the broad institute, where SKCM human biopsies were taken either from the brain (n=2) or skin (n=3).p-value comparing the expression median across groups are two-sided and computed using Wilcoxon rank-sum test. The boxplot shows the expression distribution, where the center line denotes the median and the box edges indicate the interquartile range.

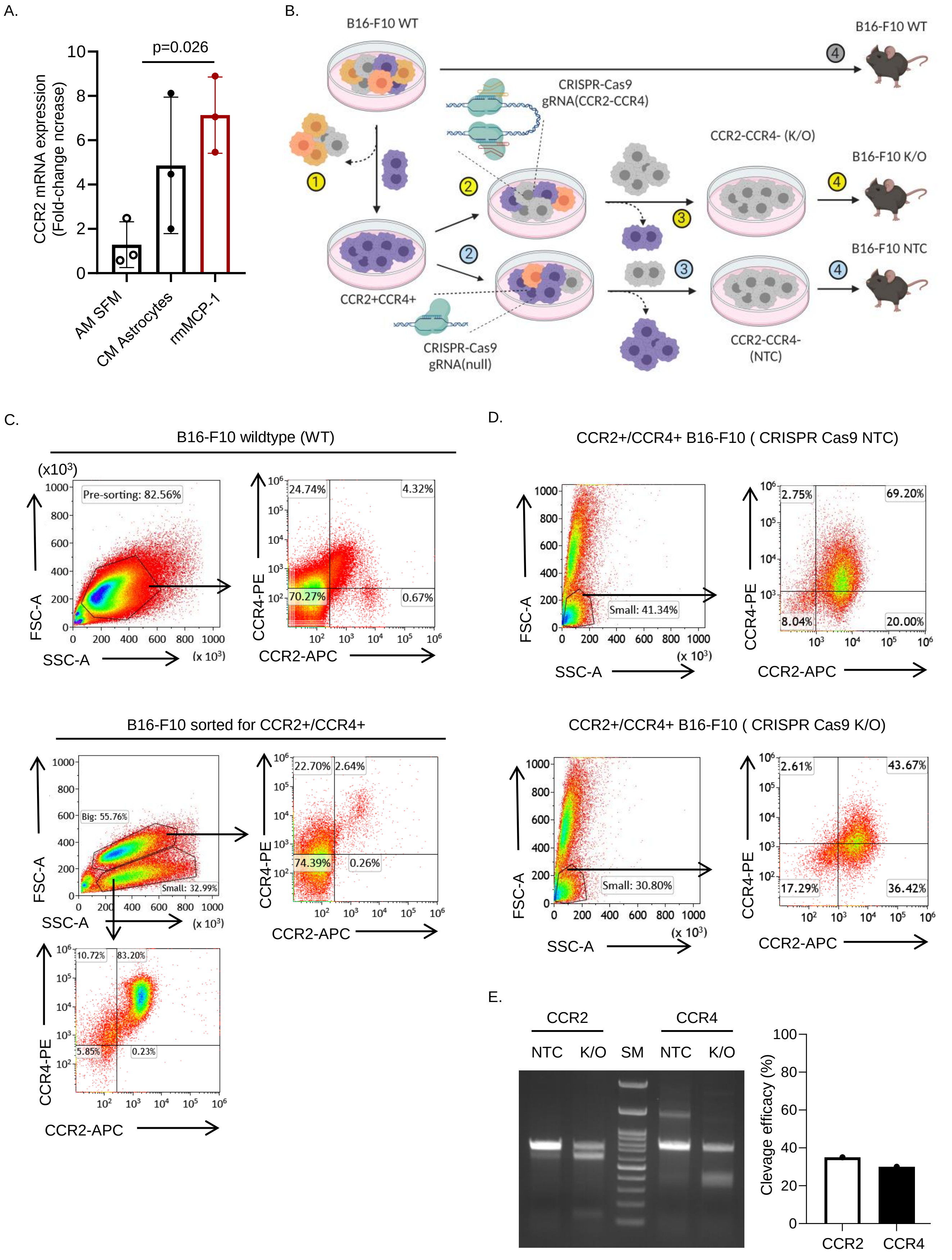
A.
p=0.026
CCR2 mRNA expression (Fold-change ncrease)
AM SFM
CM Astrocytes
rmMCP-1
B.
B16-F10 WT
CRISPR-Cas9 gRNA(CCR2-CCR4)
CCR2-CCR4- (K/O)
B16-F10 K/O
B16-F10 NTC
CCR2+CCR4+
CRISPR-Cas9 gRNA(null)
CCR2-CCR4- (NTC)
C.
B16-F10 wildtype (WT)
Pre-sorting: 82.56%
24.74%
4.32%
70.27%
0.67%
FSC-A
SSC-A
CCR4-PE
CCR2-APC
B16-F10 sorted for CCR2+/CCR4+
Big: 55.76%
Small: 32.99%
22.70%
2.64%
74.39%
0.26%
10.72%
83.20%
5.85%
0.23%
D.
CCR2+/CCR4+ B16-F10 (CRISPR Cas9 NTC)
Small: 41.34%
2.75%
69.20%
8.04%
20.00%
CCR2+/CCR4+ B16-F10 (CRISPR Cas9 K/O)
Small: 30.80%
2.61%
43.67%
17.29%
36.42%
E.
CCR2
CCR4
NTC
K/O
SM
Clevage efficacy (%)

Supplemental Figure 21. CRISPR/Cas9 CCR2 and CCR4 double experimental design. (A) Following 24 h incubation with murine astrocytes CM (mAstrocytes CM) and recombinant murine MCP-1 (rm MCP-1), CCR2 mRNA expression cells was significantly upregulated in B16-F10 melanoma (4 to 8 fold-change increase) compared to control B16-F10 cells grown in SFM of astrocytes (SFM AM). Mean ± S.D. of three independent experiments. One-way ANOVA statistical analysis was performed. **(B)** Experimental design of CRISPRCas9 K/O generation for CCR2 and CCR4 in B16-F10 melanoma cells, and subsequent injection in recipient mice. Created with BioRender.com. **(C)** B16-F10 from WT cells were analyzed by FACS for basal expression of CCR2 and CCR4, and sequentially sorted for the double positive expression of the two receptors for MCP-1. The population with smaller FSC presented CCR2 and CCR4 expression, whereas high FSC cells were negative for both markers Representative plots of n=3. **(D)** Double positive CCR2/CCR4 sorted cells were treated with CRISPR/Cas9 Ribonucleoproteins (RNP) with either the gRNAs for CCR2 and CCR4 (K/O), or without a gRNA (NTC). Representative plots of n=3. **(E)** T7 Endonuclease 1 assay demonstrating gRNA-dependent Cas9 induced double stranded breaks at the CCR2 and CCR2 loci. DNA cleavage visualized in $CCR2^{+}/CCR4^{+}$ cells treated with CCR2 and CCR4 RNPs (K/O), compared to cells treated with Cas9 but without a gRNA (NTC). n=3 repeats.

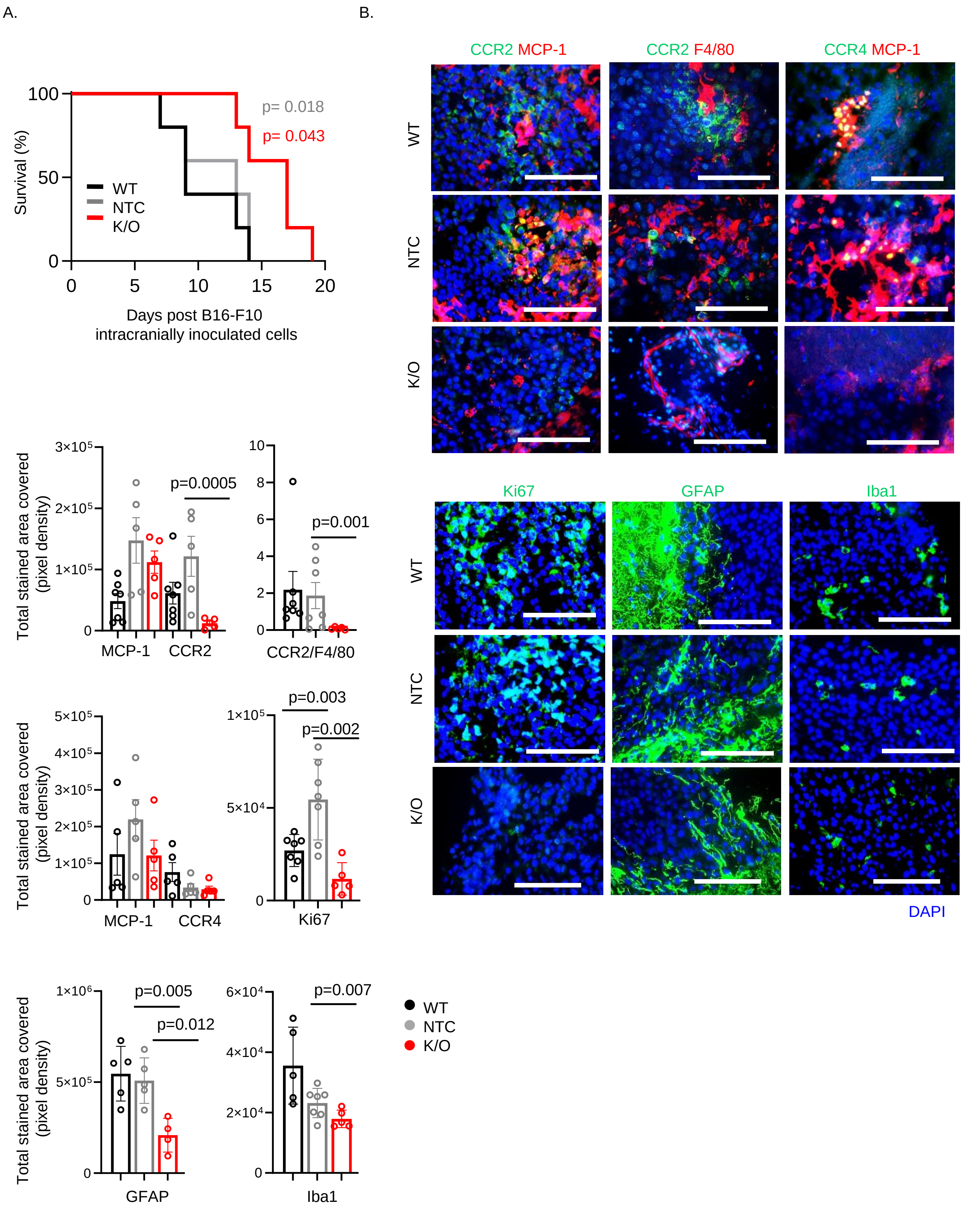

A.
B.
100
50
0
Survival (%)
p= 0.018
p= 0.043
WT
NTC
K/O
0
5
10
15
20
Days post B16-F10
intracranially inoculated cells
CCR2 MCP-1
CCR2 F4/80
CCR4 MCP-1
WT
NTC
K/O
Ki67
GFAP
Iba1
DAPI
Total stained area covered
(pixel density)
3×10⁵
2×10⁵
1×10⁵
p=0.0005
MCP-1
CCR2
10
8
6
4
2
p=0.001
CCR2/F4/80
5×10⁵
4×10⁵
3×10⁵
2×10⁵
1×10⁵
MCP-1
CCR4
1×10⁵
5×10⁴
p=0.003
p=0.002
Ki67
1×10⁶
5×10⁵
p=0.005
p=0.012
GFAP
6×10⁴
4×10⁴
2×10⁴
p=0.007
Iba1
WT
NTC
K/O

Supplemental Figure 22. CCR2/CCR4 CRISPR/Cas9 K/O improves mice survival, CD8 T-cell infiltration and decreases immune co-inhibitory molecules. (A) Kaplan-Meier plot of mice bearing B16-F10 MBM. CCR2-CCR4 K/O tumors (in red) compared to WT and NTC tumor bearing mice (in black and in grey, respectively). n=5 mice per group, two-tailed *P* values from log-rank (Mantel–Cox). **(B)** Histological analysis of MCP-1/CCR2 (red/green), MCP-1/CCR4 (red/green), CCR2 staining associated to F4/80 macrophages (red/green), proliferative rate (ki67 - green), and tumor-infiltrating Iba1 microglia/macrophages (green) in K/O, WT and NTC tumors. Nuclei are shown by DAPI staining (blue). Mean ± S.D. of n=5-7 fields per marker in n=3 mice per group. One-way ANOVA statistical analysis. Scale bar - 100 µm.

Figure 2 E: control (left lane) bindarit-treated (right lane)

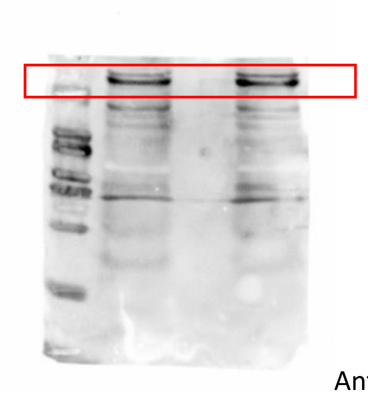

Anti p65 antibody

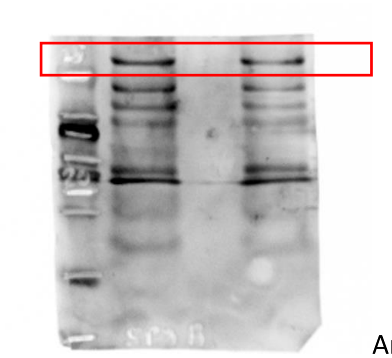

Anti p-p65 antibody

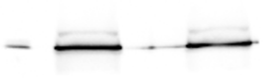

Anti Vinculin antibody
